# Quantitative monitoring of nucleotide sequence data from genetic resources in context of their citation in the scientific literature

**DOI:** 10.1093/gigascience/giab084

**Published:** 2021-12-29

**Authors:** Matthias Lange, Blaise T F Alako, Guy Cochrane, Mehmood Ghaffar, Martin Mascher, Pia-Katharina Habekost, Upneet Hillebrand, Uwe Scholz, Florian Schorch, Jens Freitag, Amber Hartman Scholz

**Affiliations:** Leibniz Institute of Plant Genetics and Crop Plant Research, Department Breeding Research, OT Gatersleben, Corrensstrasse 3, 06466 Seeland, Germany; European Molecular Biology Laboratory, European Bioinformatics Institute (EMBL-EBI), Wellcome Genome Campus, Hinxton, Cambridge, CB10 1SD, UK; European Molecular Biology Laboratory, European Bioinformatics Institute (EMBL-EBI), Wellcome Genome Campus, Hinxton, Cambridge, CB10 1SD, UK; Leibniz Institute of Plant Genetics and Crop Plant Research, Department Breeding Research, OT Gatersleben, Corrensstrasse 3, 06466 Seeland, Germany; Leibniz Institute of Plant Genetics and Crop Plant Research, Department Breeding Research, OT Gatersleben, Corrensstrasse 3, 06466 Seeland, Germany; German Centre for Integrative Biodiversity Research (iDiv) Halle-Jena-Leipzig, Puschstraße 4, 04103 Leipzig, Germany; Leibniz Institute of Plant Genetics and Crop Plant Research, Department Breeding Research, OT Gatersleben, Corrensstrasse 3, 06466 Seeland, Germany; The Harz University of Applied Science, Department of Automation and Computer Science, Friedrichstraße 57, 38855 Wernigerode, Germany; Leibniz Institute DSMZ-German Collection of Microorganisms and Cell Cultures GmbH, Department Research - Microbial Ecology and Diversity, Inhoffenstraße 7B, 38124 Braunschweig, Germany; Leibniz Institute of Plant Genetics and Crop Plant Research, Department Breeding Research, OT Gatersleben, Corrensstrasse 3, 06466 Seeland, Germany; Leibniz Institute of Plant Genetics and Crop Plant Research, Department Breeding Research, OT Gatersleben, Corrensstrasse 3, 06466 Seeland, Germany; The Harz University of Applied Science, Department of Automation and Computer Science, Friedrichstraße 57, 38855 Wernigerode, Germany; Leibniz Institute of Plant Genetics and Crop Plant Research, Department Breeding Research, OT Gatersleben, Corrensstrasse 3, 06466 Seeland, Germany; Leibniz Institute DSMZ-German Collection of Microorganisms and Cell Cultures GmbH, Department Research - Microbial Ecology and Diversity, Inhoffenstraße 7B, 38124 Braunschweig, Germany

**Keywords:** data citation, nucleotide sequence data, Europe PMC, European Nucleotide Archive, text mining, Convention on Biological Diversity, digital sequence information

## Abstract

**Background:**

Linking nucleotide sequence data (NSD) to scientific publication citations can enhance understanding of NSD provenance, scientific use, and reuse in the community. By connecting publications with NSD records, NSD geographical provenance information, and author geographical information, it becomes possible to assess the contribution of NSD to infer trends in scientific knowledge gain at the global level.

**Findings:**

We extracted and linked records from the European Nucleotide Archive to citations in open-access publications aggregated at Europe PubMed Central. A total of 8,464,292 ENA accessions with geographical provenance information were associated with publications. We conducted a data quality review to uncover potential issues in publication citation information extraction and author affiliation tagging and developed and implemented best-practice recommendations for citation extraction. We constructed flat data tables and a data warehouse with an interactive web application to enable ad hoc exploration of NSD use and summary statistics.

**Conclusions:**

The extraction and linking of NSD with associated publication citations enables transparency. The quality review contributes to enhanced text mining methods for identifier extraction and use. Furthermore, the global provision and use of NSD enable scientists worldwide to join literature and sequence databases in a multidimensional fashion. As a concrete use case, we visualized statistics of country clusters concerning NSD access in the context of discussions around digital sequence information under the United Nations Convention on Biological Diversity.

## Data Description

Nucleotide sequence data (NSD) plays a fundamental role in biological research ranging from public health and medical applications to understanding the molecular basis of life and evolution, such as how genes (mis)function in disease mechanisms [1], insights into ecosystem functioning and biodiversity conservation, and assistance in breeding new plant varieties and animal breeds to enable food security and sustainability [2]. Scientifically, NSD plays a significant role in mechanistic modelling of species evolution [3] and in genotype-phenotype correlation [4] to identify and mitigate risks to species, track their illegal trade, identify the geographical origin of products, and plan conservation management strategies [5].

These applications demonstrate the overall value of NSD use and application and have triggered a political debate about benefit sharing from genetic resources (GR). Under the Convention on Biological Diversity (CBD) and the Nagoya Protocol [6], as well as the International Treaty on Plant Genetic Resources for Food and Agriculture (ITPGRFA), the topic of “digital sequence information” (DSI) has garnered immense interest and raised concern across the international scientific community. Discussions have focused on using NSD from GRs because “DSI" is an undefined and non-scientific term. Owing to the exponential growth of public sequence and downstream databases [7], many parties are concerned that insufficient benefit sharing occurs. Datasets such as this one provide an opportunity for evidence-based policymaking to analyze global trends in NSD provision and use as well as other science policy fields, including scientific strategic development and internationalization.

With this in the background, this Data Note subsequently presents the context of the dataset for quantified NSD use. As such, the method for extracting NSD citations from the scientific literature is described, as well as the technical details of constructing the data warehouse. The “Data validation and quality control" section discusses the refinement process of the data extraction pipeline and potential shortcomings arising from the available data quality, the provided APIs, and the suggested possible improvements. Finally, the potential for reuse of the dataset through the WiLDSI web app is presented, as well as the further potential for tracking genetic resource use in the scientific literature and aspects of quantifying DSI use in the context of benefit-sharing discussions under the CBD.

### Context

The FAIR (findable, accessible, interoperable, reusable) data principles defined in 2016 in the FAIR Guiding Principles for scientific data management and stewardship [8] guide the design of open data sharing infrastructures as an enabling technology for economic growth and scientific progress. Data sharing principles were implemented at national and international levels. For example, the German Federal Ministry of Education and Research (BMBF) has funded an interdisciplinary project called “Science-based approaches for Digital Sequence Information” (WiLDSI) [9], which aims to (i) raise awareness and involve the international scientific community in the debate and decision-making process surrounding DSI, (ii) to identify and elaborate scenarios for open access to the NSD, and (iii) to establish fair and sustainable benefit sharing.

In this context, transparent quantitative measures of NSD citation and reuse can inform decision-making processes surrounding the design of data sharing infrastructure, awarding scientific “credit” or political acknowledgement, or addressing the needs of commercial users [10]. In recent years, data citation has received increased attention from publishers, funding agencies, and infrastructure providers [11, 12]. However, best practices for NSD citation are still lacking, and those developed for scientific publications cannot be readily transferred. This is especially true for NSD, hosted by the core data infrastructure, the International Nucleotide Sequence Database Collaboration (INSDC) [13]. The European Nucleotide Archive (ENA) [14] and Europe PubMed Central (ePMC) [15] are, respectively, the European partners in INSDC and a repository of open-access articles. Both have a long tradition of handling open data and document the heterogeneous quality of the author's data citation practices [16]. ePMC listed publications generally use text-embedded ENA identifiers, such as accession numbers, project accessions, or study accessions.

## Methods

**Figure 1.**
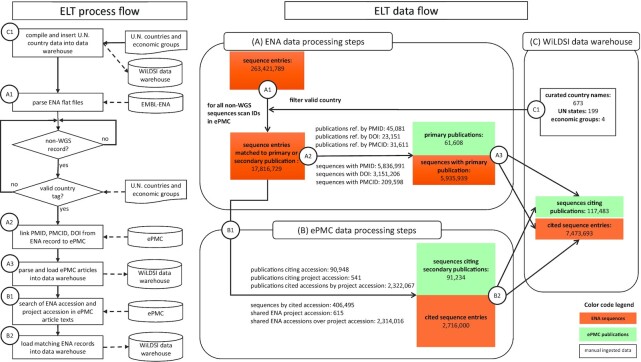
Schematic overview of the data flow of extract-load-transform (ELT) process to build the data warehouse from ENA and ePMC datasets. ENA records are parsed (A1), filtered for valid country tags, and fed into ePMC RESTful API to extract matching secondary publication (B1) by ENA accession or project accession numbers. Primary publications are linked by ENA record (A2) to the DOI, PMCID, or PMID. The resulting datasets are normalized as tables ENA_SEQUENCES, PMC_REFERENCES and loaded into the data warehouse (A3, B2). This is complemented by a manual ingested list of the world's countries and economics groups into the tables COUNTRIES and COUNTRY2GRP, respectively (C1). Finally, SQL queries are applied to generate charts and reports in the Web application.

**Figure 2.**
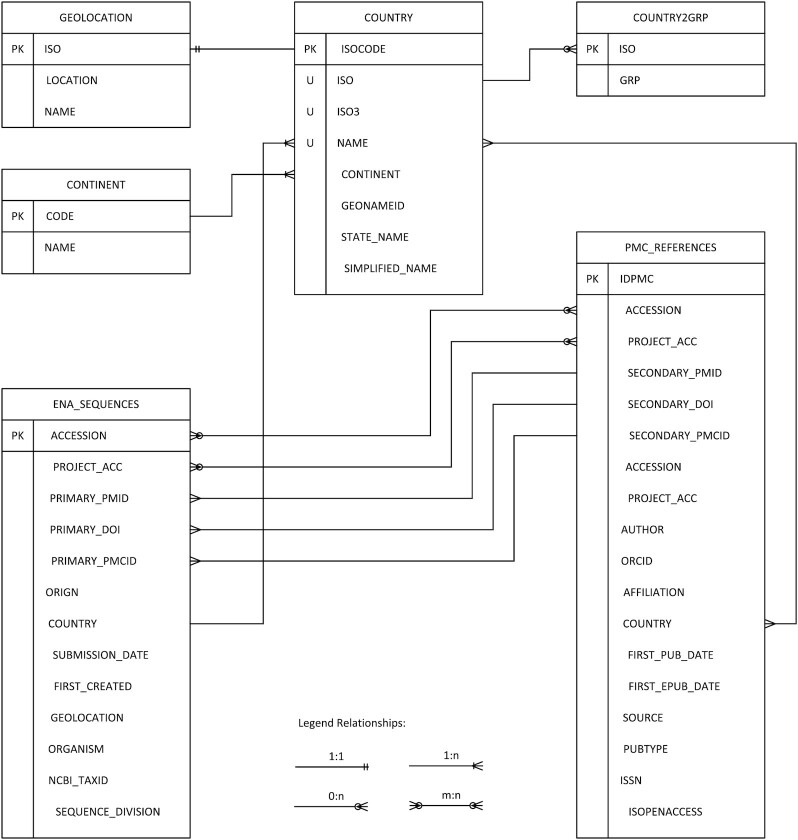
Table schema of the WiLDSI data warehouse. The table ENA_SEQUENCES comprise metadata of a sequence stored in the EBI ENA database. The attributes “accession" and “project accession" are used to join secondary literature that cites sequences. The attribute “country" refers to the country table to resolve and group country-tagged ENA sequences. The table “PMC_REFERENCES" consists of all ePMC published papers referencing an ENA sequence by either accession or project accession and references from ENA records as primary publication by either a DOI, PMID, or PMCID.

Figure 1 shows the extraction-load-transform (ELT) of ENA citations and the resulting data flow. First, ENA accessions and project accession numbers were extracted. Literature citations listed directly in the ENA entry were extracted in parallel and called hereafter “primary publications.” Next, we retrieved scientific articles that referred to these accession IDs via a full-text search using the ePMC REST API [17]. These publications we labelled “secondary publications.” Finally, the extracted references and associated citation information were organized into 6 tables and imported into a data warehouse. As documented in Fig. 2, its schema shows the referential dependencies, foreign keys, and primary key attributes. This is intended to support the advanced use of data exploration functionality in the web application. It is also intended to reduce users' barriers to reusing the raw table dumps for downstream analysis.

Five classes of citation patterns are used for ePMC publication as ENA identifiers where used: single accession number using word separation characters, e.g., hyphens, brackets, quotation marks; range notation of referenced accessions; text-embedded enumeration; lists in supplementary material; or even citations embedded into figure bitmaps. The process of data extraction from EMBL-ENA and ePMC was executed by Perl and Python scripts. The starting point of the data extraction process is the EMBL-ENA flat-file dump of release 143, which was obtained from the EMB-ENA FTP server and comprised 263,421,789 records. Next, all non–whole-genome sequence (WGS) ENA records were parsed to compile a relevant set of attributes for the table “ENA_SEQUENCES.” A total of 17,820,136 ENA accessions with valid country tags (i.e.. the/country field in the ENA entry, comprising 15% of all records) were included. Next, using the ePMC REST API, these ENA accessions were scanned in 36.7 million full-text articles accessible via PubMed. Owing to performance reasons, this text tokenization was executed on-site at EBI in a compute cluster environment. From the resultant publications, those were selected that have valid author country information and that either (i) cite an ENA sequence as a secondary publication (designated as secondary in CBD context) or (ii) are cited by ENA record as a primary publication (designated as primary in CBD context). The publications matching these criteria were compiled into the table “PMC_REFERENCES.” In detail, 5,935,939 sequences cite 61,608 publications and 2,716,000 sequences are cited by 91,234 publications, respectively. All scripts used in our analyses are available in GitHub [18].

The table “COUNTRY” was compiled and curated from UN state membership [19]. It comprises the 3 kinds of ISO-3166–1-codes, the official name (e.g., United Kingdom of Great Britain and Northern Ireland), a short version of the state name (e.g., the United Kingdom), commonly used names (e.g., Great Britain), and continent assignments. This table allows mapping from partly ambiguous country affiliation used in articles to the actual country designations recognized under international law. In particular, provinces or (partially) autonomous areas, such as Taiwan or West Sahara, are mapped to the legally responsible UN state party. Furthermore, several ocean areas, for example, “Bismarck Sea” or “East China Sea,” are grouped under the “Ocean” label along with more standard fields such as “Atlantic Ocean” (It should be noted that “Ocean" is not equivalent to international waters under the United Nations Convention of the Law of the Seas (UNCLOS, where marine genetic resources and benefit-sharing are beeng discussed) but is in this context simply a consolidated term representing sampling in the marine environment). The assignment to economic groups is stored in table “COUNTRY2GRP.” Here a 2-letter ISO code is assigned to rough economic groups OECD (Organization for Economic Cooperation and Development), BRICS (Brazil, Russia, India, China, South Africa), and G77 (representative of developing economies). To visualize countries in a world map, we used the table “GEOLOCATION” comprising the coordinates of the centroid of each country.

The tables are provided for download as CSV files (see section “Data Availability”). A data warehouse was built on the basis of the extracted dataset to support online analytical data processing and convenient data access. To scale appropriately, an ORACLE Enterprise RDBMS was applied. It enables analytical real-time SQL queries over millions of data points using in-memory materialized views, vectorization, and columnar storage. This, in turn, was the basis to guarantee an appropriate user experience for the subsequently presented web application, which provides interactive, online calculation of metrics from NSD citations to various filters and data groupings, to drill down chart and link data to the original records in ENA and ePMC.

### Data validation and quality control

To assess the reliability of the extracted ePMC to ENA references, potential quality issues were evaluated by plausibility scans across data warehouse tables, including inspection of 20 randomly sampled articles performed by domain experts from the sequence submission service team of the Leibniz Institute of Plant Genetics and Crop Plant Research. We also considered review articles on data identifiers in the life science literature [20, 21]. Finally, we applied the Dimensions text-mining tool [22] to cross-check the sensitivity of ePMC API in respect of recall and sensitivity, e.g., to find false-negative hits such as published articles that reference ENA sequences but were not matched by the ePMC REST API.

### Country names

The country name had to match records in the country table. Here we found some obsolete or ambiguous country names, such as Montenegro or West Sahara, and historic country names, such as the Soviet Union, which cannot be assigned uniquely to current UN states. Ambiguous country names were resolved manually and reverted to synonyms in the country table (e.g., Cote d'Ivoire to Ivory Coast, amongst others). ENA or PMC records with obsolete country tags were kept in the dataset but ignored for summary statistics queries and excluded in the below quality check.

### Retrieval of referenced IDs

Next, reference consistency among the extracted ENA and ePMC records was checked by extract-load-transform (ELT) test runs. This resulted in a preliminary data warehouse instance, which allowed SQL-based plausibility checks, such as counting unique article identifiers, ENA accessions, and country tags or counting the number of records in preliminary joining of ENA and ePMC records over a different combination of PMID, PMCID, and DOI. We specifically checked whether ENA records refer to a valid country. For 217,40 ENA records out of 18,034,192, this was not the case, e.g., country tag “Western Sahara” (ENA accession HM034625) or empty country tag (ENA accession KM654101). Those could, in some cases, be resolved by manual addition of synonyms to the country table that reflect the current valid UN-agreed assignments. In the case of empty country tags, we found that some were annotated with geographical location. However, we left them empty to avoid a non-transparent change of primary data.

Furthermore, we checked whether primary papers referenced in an ENA record exist in the ePMC databases. Here, a total of 6,753,891 ENA records refer either by DOI, PMID, or PMCID to 351,119 ePMC records, i.e., some use DOI only, some DOI and PMID, and so forth. Conversely, there are 9,589,900 ENA records without any primary literature reference. Furthermore, we confirmed that ePMC records, which cite secondary ENA accession or project numbers, can be resolved to records in ENA. We found 189,581 ePMC records that reference 2,801,072 ENA records by either accession or project accession number. A potential issue in using an identifier to cite ENA records is that authors sometimes use ENA study identifiers or even BioSample IDs. However, our pipeline considers ENA accession and project accession only.

### Author identification

The combination of first and last names does not constitute a unique identifier for human beings. ORCIDs provide unique identifiers for authors and are on their way to becoming compulsory for publications. Existing articles, however, are only occasionally associated with ORCID. Another potential issue is that it is possible to register multiple ORCIDs for 1 person. Identifying authors as a concatenation of author names and affiliations is error-prone [23, 24]. Therefore, author information was retained in the tables but not used for statistical analysis.

### Range notation

**Figure 3.**
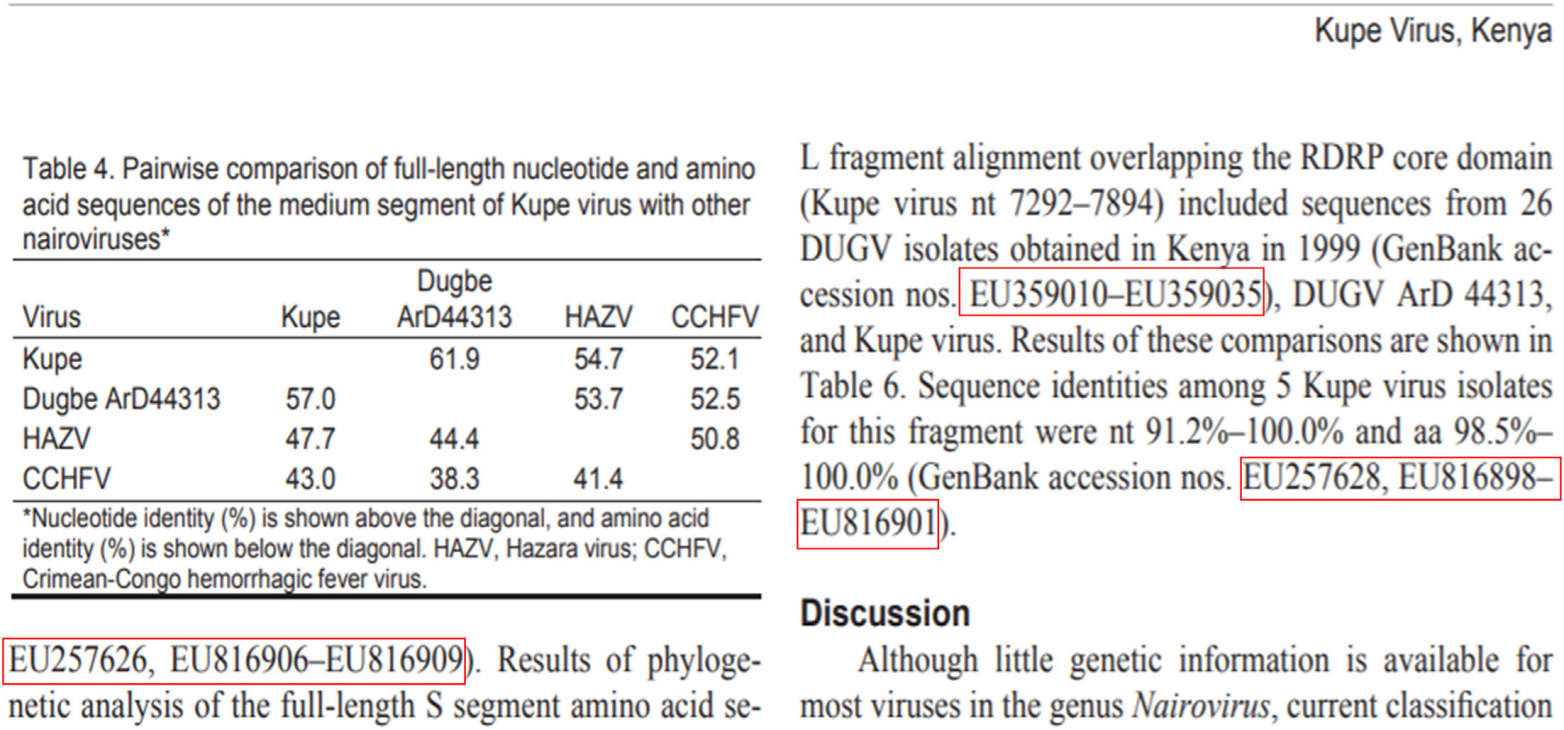
Example of range notation for ENA accession references. Within the selected part of Crabtree et al. [39], the actual number of cited ENA accessions is 35, but ePMC API matched only 8.

Scientific publications may use ambiguous range notation to cite ENA accessions. As illustrated in Fig. 3, hyphens as range notation aggregate a sequence of ENA accessions. Here, the authors assume an ordered sequence of accession numbers, and it is interpreted as such by human readers but is not recognized by programmatic text mining. Thus, in the data extraction used here, a potentially high number of ENA accessions are missed, and the dataset underestimates the number of referenced DSI. This analysis is intended to support future work to address these shortcomings.

### Access restrictions

Only open-access publications were available for high-throughput text mining. To efficiently process 18 million ENA accession numbers, the ePMC REST API at EBI's local compute infrastructure was used. This causes a potential loss of recall compared to broad and integrative use of further state-of-the-art literature-mining services that include articles behind paywalls. To get an estimation of potentially missed DSI citations, we used alternative tools that cover patent and closed-access publications. We applied the commercial “Dimensions” [22] and the free “Lens.org” [25] search tools, which include patent and restricted-access publication, to compare recall performance for 20 randomly selected ENA accessions. This evaluation was performed within 4 weeks of the ePMC-based text-mining run to work with a comparable corpus. The results are compiled in Table 1. Specific hits to 1 of 3 approaches, ePMC, Lens, and Dimensions, were observed. This is likely due to the larger corpora of Dimensions and Lens. For example, ENA accession AB076935 was linked to 3 public and 3 closed-access publications, whereas ePMC did not report any matching publication. Differences in file format may explain some of the differences. There are cases where the PDF-rendered articles differ from ePMC-rendered HTML versions so that the PDF versions can contain more ENA accession numbers than HTML versions in ePMC. We did not aim for an in-depth analysis of literature search tools. Still, our cursory overview supports the notion that a substantial number of publications relevant to NSD may be behind paywalls.Scanning PDF-encoded manuscripts, using sophisticated text-mining methods, and integrating commercial text-mining software could improve the recall and precision of NDS citation in texts as well. However, in the spirit of the project in which this analysis took place, with a heavy emphasis on open access and ENA API, we continued our analyses with the available dataset.

**Table 1.** Comparison of ENA accession number query performance of APIs of EBI ePMC, Dimensions, and Lens

ENA Accession	Hits in ePMC	Hits in Dimensions	Hits in Lens	Overlap Dimensions and ePMC	Overlap Lens and ePMC
AB076935	0	6	0	0	0
AB076941	0	1	0	0	0
EU257628	3	5	0	2	0
AB326609	0	1	0	0	0
AM262332	0	2	0	0	0
EU575854	1	1	0	1	0
CP039348	0	1	0	0	0
DQ410599	1	1	0	1	0
EU293114	12	19	1	6	1
AY924392	10	7	2	6	2
EF607913	0	1	0	0	0
AY768827	0	1	0	0	0

Dimensions queries used the URL pattern https://app.dimensions.ai/discover/publication?search_text=AY924392&search_type=kws&search_field=full_search, whereas Lens queries used the URL pattern https://www.lens.org/lens/scholar/search/results?q=AY924392&preview=true.

## Reuse potential

**Figure 4.**
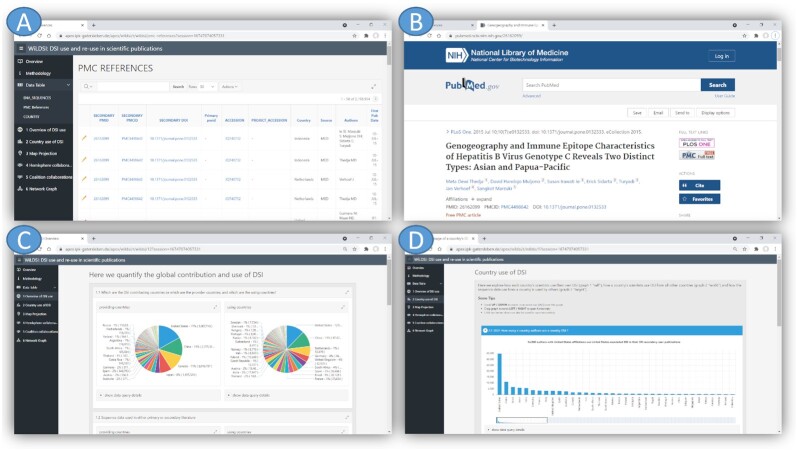
Screenshots of the WiLDSI Web Application. It consists of pages for (A) detailed data reports with integrated (B) drill-down to sources, (C) charts of DSI usage scenarios, (D) per country DSI use and contribution, and so forth.

To further explore the dataset, a web application was developed and is publicly accessible at [26]. We focused mainly on understanding NSD/DSI usage in the context of fair and equal benefit sharing. The web interface illustrated in Fig. 4 enables the interactive exploration of DSI use in science by a features text search, data aggregation across the data warehouse, and cross-linking to the original ENA records and ePMC records. It enables further complex filtering and grouping, as well as visualization as charts, world map projects, and network diagrams. On the basis of the use cases provided in this CBD context, fundamental questions regarding DSI usage are visualized in different relationships to answer questions such as: Which countries use DSI? Which countries (groups) contributed DSI? Are there countries that use DSI but do not contribute DSI? Five classes of use cases implement this: “general overview of DSI," “per country use of DSI," “collaborative use in economic and hemisphere groups," “world map projection," and “DSI citation network."

Another reuse scenario is to document the flow of DSI associated with gene bank resources across the scientific value chain from seed storage to genetic analysis. A prominent example is the role of molecular passport data as an instance of DSI to characterize plant genetic resources (PGR). Gene bank genomics is an emerging research field aiming at using high-throughput sequencing to characterize the genetic diversity in entire gene bank collections [27]. Recently, marker profiles from reduced representation sequencing data were reported for >20,000 accessions of the German Genebank [28]. Whole-genome shotgun sequencing has been used to characterize the genome of 3,000 rice accessions at the International Rice Research Institute [29]. The approach provides a so-called molecular passport that enables tracking the identity of accessions, identifying redundancies, and cross-linking international gene banks [30]. For these reasons, molecular passport data is poised to become an essential component of working with PGR in research and breeding contexts. Documenting the use of DSI associated with PGR would help gene bank managers and administrators of gene bank information systems monitor the use of their accessions in international research efforts and help justify the tremendous effort put into the maintenance and characterization of PGR in global gene banks. Documenting DSI could also help national authorities to enforce access and benefit-sharing schemes of the Nagoya protocol. The present enquiry into the status of DSI in public sequence archives has shown that sequence information of PGR is abundant, but tracing it back to the gene bank holdings it derives from can be challenging. In the coming years, gene bank managers, genome researchers, and bioinformaticians should develop and enshrine standards and protocols for linking DSI in archives such as EMBL-ENA to gene bank information systems and meta-databases such as as EURISCO [31]. Work in this direction is underway in the EU-funded project AGENT [32].

DSI and free access to it are essential for all life sciences, including biodiversity research, food security, human health, biological conservation, and many other disciplines or research areas. Some countries contributing DSI fear that direct access to the increasing amount of freely available sequence information may undermine benefit-sharing schemes for genetic resources. The use of this dataset supports evidence-based decision making in the context of international policy processes and global-scale investigations into scientific use and reuse of NSD datasets and sub-disciplines thereof. Indeed, this article is intended as a companion paper for a timely publication on the policy implications of NSD (re-)use for DSI access and benefit-sharing discussions under the CBD published alongside it in this journal [33].

For future studies, the aforementioned examples could be complemented by more detailed use cases, including finer-grained groupings for data aggregation such as separation of genera, species, and time ranges of publications. In combination with additional text classification techniques [34], it may be possible to cluster by research topics, e.g., considering only citations in the article involving, say, COVID-19 or plant pathogen resistance.

## Availability of Source Code and Requirements

Project name: WiLDSI

Project home page: https://wildsi.ipk-gatersleben.de

Operating systems: LINUX

Programming language: Oracle Application Express, Perl, Python3

Other requirements: HTML5-compatible web browser

License: GNU General Public License v3.0

All scripts used for data extraction are available from GitHub [35].

## Data Availability

The charts, maps, and data tables are available in an interactive web application at [26]. The data tables are published as CSV files in the e! DAL-PGP repository [36] under doi: 10.5447/ipk/2021/8 [37]. The SQL queries implementing the use cases are linked and documented alongside each chart within the web application. An archival copy of the GitHub repository is available in the GigaScience GigaDB repository [38].

## Abbreviations

API: application programming interface; CBD: Convention on Biological Diversity; DSI: digital sequence information; ELT: Extract, Load, Transform; EMBL: European Molecular Biology Laboratory; ENA: European Nucleotide Archive; ePMC: Europe PubMed Central; GR: genetic resources; INSDC: Nucleotide Sequence Database Collaboration; ITPGRFA: International Treaty for Plant Genetic Resources for Food and Agriculture; NSD: nucleotide sequence data; ORCID: Open Researcher and Contributor ID; PGR: plant genetic resources; WiLDSI: German: “wissenschaftsbasierte Lösungsansätze für digitale Sequenzinformation,” English translation: Science-based Approaches for Digital Sequence Information.

## Competing Interests

The authors declare that they have no competing interests.

## Funding

This work was supported by the German Federal Ministry of Education and Research (BMBF) in the frame of the project “WiLDSI: Wissensbasierte Lösungsansätze für Digitale Sequenzinformation” (FKZ 031B0862) and core funding of Leibniz Institute of Crop Plant Research Gatersleben.

## Authors' Contributions

Conceptualization: A.H.S., J.F., G.C., M.L.

Software: M.G., B.T. F.A., M.L., P.H., F.Z.

Data curation: U.H., J.F., M.L.

Investigation: A.H.S., J.F., U.H.

Supervision: M.L., A.H.S., J.F., G.C.

Writing original draft: M.G., M.L., M.M., A.H.S.

Writing review and editing: All authors.

Funding acquisition: A.H.S., U.S.

## Competing Interests

The authors declare that they have no competing interests.

## Supplementary Material

giab084_GIGA-D-21-00130_Original_Submission

giab084_GIGA-D-21-00130_Revision_1

giab084_GIGA-D-21-00130_Revision_2

giab084_Response_to_Reviewer_Comments_Original_Submission

giab084_Response_to_Reviewer_Comments_Revision_1

giab084_Reviewer_1_Report_Original_Submission Gianmaria Silvello, Ph.D. -- 5/10/2021 Reviewed

giab084_Reviewer_1_Report_Revision_1 Gianmaria Silvello, Ph.D. -- 8/31/2021 Reviewed

giab084_Reviewer_2_Report_Original_Submission Michael Fire, Ph.D -- 5/25/2021 Reviewed

giab084_Reviewer_2_Report_Revision_1 Michael Fire, Ph.D -- 9/12/2021 Reviewed

giab084_Reviewer_3_Report_Original_Submission Takeru Nakazato, Ph.D. -- 5/31/2021 Reviewed

giab084_Reviewer_3_Report_Revision_1 Takeru Nakazato, Ph.D. -- 8/24/2021 Reviewed
