# Supplementary material for: Quantitative monitoring of nucleotide sequence data from genetic resources in context of their citation in the scientific literature
Source: Gigascience. 2021 Dec 29;10(12):giab084. doi: 10.1093/gigascience/giab084 (PMC8716361; doi:10.1093/gigascience/giab084)
Supplement: giab084_GIGA-D-21-00130_Revision_1 [file giab084_giga-d-21-00130_revision_1.pdf]

GigaScience

Quantitative monitoring of nucleotide sequence data from genetic resources in context of their citation in the scientific literature

--Manuscript Draft--

**Manuscript Number:**	GIGA-D-21-00130R1
**Full Title:**	Quantitative monitoring of nucleotide sequence data from genetic resources in context of their citation in the scientific literature
**Article Type:**	Data Note
**Funding Information:**	Bundesministerium für Bildung und Forschung (031B0862) — Dr Amber Hartman Scholz
**Abstract:**	Background<br><br>Linking nucleotide sequence data (NSD) to scientific publication citations can enhance understanding of NSDs provenance, scientific use, and re-use in the community. By connecting publications with NSD records, NSD geographical provenance information, and author geographical information, it becomes possible to assess the contribution of NSD to infer trends in scientific knowledge gain at the global level.<br><br>Findings<br><br>For this data note, we extracted and linked records from the European Nucleotide Archive to citations in open-access publications aggregated at Europe PubMed Central. A total of 8,464,292 ENA accessions with geographical provenance information were associated with publications. We conducted a data quality review to uncover potential issues in publication citation information extraction and author affiliation tagging and developed and implemented best-practice recommendations for citation extraction. Flat data tables and an data warehouse with an interactive web application were constructed to enable ad hoc exploration of NSD use and summary statistics.<br><br>Conclusions<br><br>The extraction and linking of NSD with associated publication citations enables transparency. The quality review contributes to enhanced text mining methods for identifier extraction and use. Furthermore, the global provision and use of NSD enables scientists around the world to join literature and sequence databases in a multidimensional fashion. As a concrete use case, statistics of country clusters were visualized with respect to NSD access in the context of discussions around digital sequence information under the United Nations Convention on Biological Diversity.
**Corresponding Author:**	Matthias Lange<br>Institute of Plant Genetics and Crop Plant Research: Leibniz-Institut fur Pflanzengenetik und Kulturpflanzenforschung Gatersleben<br>Seeland, Sachsen-Anhalt GERMANY
**Corresponding Author Secondary Information:**	
**Corresponding Author's Institution:**	Institute of Plant Genetics and Crop Plant Research: Leibniz-Institut fur Pflanzengenetik und Kulturpflanzenforschung Gatersleben
**Corresponding Author's Secondary Institution:**	
**First Author:**	Matthias Lange
**First Author Secondary Information:**	
**Order of Authors:**	Matthias Lange
	Blaise T.F. Alako

Powered by Editorial Manager® and ProduXion Manager® from Aries Systems Corporation

	Guy Cochrane
	Mehmood Ghaffar
	Martin Mascher
	Pia-Katharina Habekost
	Upneet Hillebrand
	Uwe Scholz
	Florian Zunder
	Jens Freitag
	Amber Hartman Scholz
**Order of Authors Secondary Information:**	
**Response to Reviewers:**	Dear editors, dear reviewers,<br><br>We are very appreciative of the review and comments on our article. In the following letter we answer all questions and provide comments on the intended revisions. All revisions in the manuscript are highlighted in red. To address the editor's suggestion to provide more insight into the implications and analysis of the data sets presented, we have submitted a tandem “policy paper” (your submission identifier GIGA-D-21-00228), published a pre-print in BioRxiv (DOI: 10.1101/2021.08.02.454535) and have added further explanatory text on each of the diagrams in the web application.<br><br>The point-by-point response to the reviewers' comments can be found hereafter.<br><br>Answers to comment of reviewer #1<br><br>Comment 1.1:<br>Figure 1 is not readable. The sampling process lowered the quality of the image and made the text not readable. Please, use vectorial images (e.g., PDF or EPS). Anyhow, I could understand the process from the descriptive text.<br><br>Response 1.1:<br>Sorry for the low quality of the rendered PDF. The figures’ resolution was increased to 1200 dpi and the transparent background was replaced by white to avoid potential PDF rendering issues.<br><br>Comment 1.2:<br>Figure 2 is readable, but the quality is relatively low. Nevertheless, I do not think this figure is instrumental; it is a simple logical schema of a relational database. Uploading the SQL dump or the SQL schema in an external repository and reference it in the paper would be enough.<br><br>Response 1.2:<br>The image resolution was increased. The detailed referential relationships between the data entities at the attribute level are visualised in the ER diagram. These relationships are important for readers who will go on to use the data set in two ways: first, deeper functionality in the Web app. requires a knowledge of the underlying schema; second, the immediacy of the visualization in the paper will reduce the barrier for users to re-use the raw table dumps for downstream analysis. This intention is now better expressed in the manuscript in the methods section on page 5:<br>“Its schema, as documented in Figure 2, shows the referential dependencies, foreign keys, and primary key attributes. This is intended to support advanced use of data exploration functionality in the web application. It is also intended to reduce the barrier for users to reuse the raw table dumps for downstream analysis.“<br><br>Comment 1.3:<br>The sentence "we imported an ORACLE SQL data warehouse that employs state-of-the-art database technologies" is not very clear. What do you mean by "imported a data warehouse"? Could you provide more details about the DBMS you used? To my understanding, you designed a relational model. You then implemented it in SQL using

an Oracle DBMS (MySQL? or the native Oracle DBMS?) to store and query the data. Check page 9 description and add some details to avoid confusion. This is not a key passage though, I am sure that you handled the data somehow, and the paper's focus is not on this.

Response 1.3:
Thanks for this useful hint to consider more technical details of our database infrastructure. We revised the mentioned sentence in the manuscript to:

“To support on-line analytical data processing and convenient data access, a data warehouse was built based on the extracted data set. To scale appropriately, an ORACLE Enterprise RDBMS was applied. It enables analytical real-time SQL queries over millions of data points by means of in-memory materialized views, vectorization and columnar storage. This in turn was the basis to guarantee an appropriate user experience for the subsequently presented web application, which provides interactive, on-line calculation of metrics from NSD citations to various filters and data groupings, to drill-down chart and link data to the original records in ENA and ePMC.”

Comment 1.4:
"reference integrity between the tables was checked" -> This is a "weird" statement. Reference integrity is a constraint to guarantee the consistency of data. You "check the integrity" when you store the data in the DB, and if it is not validated, the data cannot be stored in the DB. So, I do not understand this sentence that is not explained anymore. Indeed, the paragraph continues by talking about the SQL queries to count the paper identifiers (this is not directly linked to reference integrity, or at least you should explain what you mean).

Recent analysis about issues related to ORCID ids and duplication of ids can be found here: http://ceur-ws.org/Vol-2816/paper10.pdf

Response 1.4:
We agree with the reviewer that reference integrity is a constraint to guarantee the consistency of data. In the manuscript we were unclear about the level of its use. In contrast to an OLTP centered database implementation of consistency checks by check constraints, such as foreign keys, we applied the reference integrity check in the ETL process to uncover potential reference integrity issues in the data set that results from the data extraction process. Indeed, we used a preliminary data warehouse instance without any formal SQL-DDL foreign key constraints, but SQL-based plausibility checks. E.g. by counting unique paper identifiers, ENA accessions, and country tags and checked number of joinable ENA and ePMC records. This lead to improvements of country references by the add country names synonyms to the country table or the use of a logical OR operator to join the ENA primary publication to ePMC by PMID, DOI or PMCID. A further example was the revision of the first approach to extract ENA accession number as reference to ENA from ePMC records. Because of obvious mismatch of expected counts to actual extracted ones for some exemplar papers, like genome assemblies of plants, we concluded that project accession numbers should also be considered as ENA citation too and revised and expanded the data mining task respectively.

In this context we thank the reviewer for this comment and improved the mentioned paragraph “Retrieval of referenced IDs”:

“Next, reference consistency among the extracted ENA and ePMC records were checked by ETL test runs. This resulted in a preliminary data warehouse instance, which allowed SQL-based plausibility checks. Such as the count unique paper identifiers, ENA accessions, and country tags or count the number of records in preliminary joining ENA and ePMC records over different combination of PMID, PMCID and DOI. We specifically checked whether ENA records refer to a valid country. For 217.40 ENA records out of 18.034.192 was it not the case, e.g. country tag “Western Sahara” (ENA accession HM034625) or empty country tag (ENA accession KM654101). Those could in some cases resolved by manual addition of synonyms to the country table that reflects the current valid UN agreed assignments. In case of empty country tags, we found some were annotated with geographical location. However, we left them empty to not intransparently change primary data.

Powered by Editorial Manager® and ProduXion Manager® from Aries Systems Corporation

Further we checked, if primary papers referenced in an ENA record exist in the ePMC databases. Here, a total of 6,753,891 ENA records refer either by DOI, PMID, or PMCID to 351,119 ePMC records, i.e. some use DOI only, some DOI and PMID etc.”

In respect to the comment “Recent analysis about issues related to ORCID ids and duplication of ids can be found here: http://ceur-ws.org/Vol-2816/paper10.pdf” we added this reference to the manuscript as new citation [24]:

“Baglioni, M., Mannocci, A., Manghi, P., Atzori, C., Bardi, A., and Bruzzo, S. L. Reflections on the misuses of ORCID ids. In CEUR Workshop Proceedings of the 17th Italian Research Conference on Digital Libraries, http://CEUR-WS.org. pp. 117–125, 2021.”

Comment 1.5:
Table 1 is not that useful; it can be described in the text that you did the experiment and verified the discrepancies between open access publications and paywalled papers. It is a well-known problem, and it is not analyzed in-depth here. I think you can get rid of it without affecting the quality of the paper.

Response 1.5:
We agree that Table 1 reflects the issues formulated already in the text. A further intention was to show particular cases, where the suggested improvements were beneficial. Following the intention of the GigaScience Data Note policy, the intention here is to give additional interested parties, like software developers, publishers, or data curators some examples for further investigation. Thus, we would like to keep Table 1.

Comment 1.6:
Figure 4, like all the other images, is not readable. I directly accessed the Webapp, which works fine.

Response 1.6:
The quality was improved by increasing the resolution to 1200 dpi. We are sure that the rendering process at the editorial office may benefit and the resulting PDF will have a better readability.

Comment 1.7:
The paper is well-written, and the data collection is fine. Nevertheless, the article is a bit anti-climatic because there, not many provided insights. You discuss what we can do with the data, but little analysis of the data themselves. We could use some more in-depth analysis and a few insights about the achievable outcomes we can get using the collected data. Also, more about the best practices that should be defined in the field would be a nice addition.

Response 1.7:
This observation is particularly true and result of the authors’ decision to separate the findings of the underlying study into a technical dataset paper and a policy-focused paper about the analysis, interpretation and conclusions. This paper was announced to the editorial board within the submission process and was submitted to GigaScience (submission ID GIGA-D-21-00228). In order to keep a reasonable timeline for publication, we would like to follow the editor’s suggestion and publish a pre-print of a policy analysis paper (DOI: 10.1101/2021.08.02.454535). Furthermore, we will give more background in the WebApp in the details panel below every chart. This will provide users of the data more insight as well.
In respect to the suggested addition to best practice we are note sure about the reviewers intension. Regarding a technical aspect concerning the citation practice of NSD in scientific literature, conclusions can be drawn from the methods section that authors should reference the used sequences as explicitly as possible via a list of individual accession numbers or project accessions according to the citation policy of the respective journals. This aspect was indicated in the section "Data validation and quality control".
Another aspect would be the consistent labeling of the origin of biological material. As noted in section Methods, 15% of the ENA sequences are country tagged. This implies that appropriate regulation on the part of the data repositories could be debated. Although the discussion of this aspect is not in the scope of this data note, the

Powered by Editorial Manager® and ProduXion Manager® from Aries Systems Corporation

publication of this manuscript could certainly support a discourse.
A best practice in terms of implications for the use and provision of DSI in the context of the CBD negotiations is a comprehensive discourse to which this data note contributes a factual basis. The resulting possible interpretations and implications will be explained in a companion paper as mentioned before.

Answers to comment of reviewer #2
The idea of curating this dataset is both important, and can contribute to the scientific community. Additionally, in most parts, the paper is well written.
However, the manuscript has some major issue that needs to solve before it would be ready for publication.

The Good:
- The dataset presented in the paper can be very useful to the scientific community
- The authors invested many efforts in making the paper reproducible. Both the project's code and dataset are open
- The project has a friendly and helpful web interface.

Things that need to improve:

Comment 2.1:
Although this paper is not a standard research paper, the article is missing more context to other works.
I believe the context of the manuscript will be more explicit by adding a Related Work section that provides an overview of other papers that generated similar datasets.

Response 2.1:
Please see our responses to similar comments by reviewer 1 in response 1.7.

Comment 2.2:
-Most of the analysis is based on the PubMed datasets, which is a relatively small dataset.
There are other open datasets that I think it is important to use to get a fuller picture, such as Microsoft Academic, AMiner, Semantic Scholar, bioXiv, and arXiv.
I understand that performing a full-text search on these datasets can be challenging. However, the paper's results need to be validated by using some of these datasets.

Response 2.2:
The goal of the study, in the first place, was not to extract all links, however. Rather, the data integration pipeline presented should achieve a good balance between recall and precision with respect to the data integration scenario at hand. The partner in the underlying research project is the EBI. The EBI databases were used with the goal of evaluating possible approaches to quantify DSI usage. In addition, the use case for the analysis was to estimate DSI use in science for policy makers. We also recognize a focus on open access and the value that these open datasets provide. ePMC-API was therefore accepted as a reasonable trade-off between the computational burden of screening more than 18 million ENA accessions and a potential loss of sensitivity. However, we agree with this comment and will continue to develop the identifier text extraction pipeline in a follow-up project that has already been approved. Some possible improvements have been highlighted in the Data Validation and Quality Control section of the Access Constraints section.
This rationale is now reflected by a text improvement in the "Access Restrictions" section on page 11.

“..., scanning PDF encoded manuscripts, the use of sophisticated text mining methods and integration of commercial text mining software could improve the recall and precision of NDS citation in texts as well. However, as the spirit of the project in which this analysis took place, with a heavy emphasis on open access and ENA API …”

Comment 2.3:
The manuscript's quality needs to be improved (text, figures' resolutions, etc.).

Powered by Editorial Manager® and ProduXion Manager® from Aries Systems Corporation

Response 2.3:
As reported in response 1.1 to a similar feedback of reviewer 1, the figures' resolution was increased to support a suitable PDF rendering in the GigaScience manuscript processing pipeline.

Comment 2.4:
In my opinion, the overall structure of the paper can be improved.

Response 2.4:
Thanks for this comment. The structure of the paper followed the GigaScience authors instruction for data notes. In order to meet this hint, we included before the first subsection "Context" at page 3 an overview of the paper content:
"With this in the background, this Data Note subsequently presents the context of the dataset for quantified NSD use. As such, the method for extracting NSD citations from the scientific literature is described, as well as the technical details of constructing the data warehouse. The Data Validation and Quality Control section discusses the refinement process of the data extraction pipeline and potential shortcomings arising from the available data quality, the provided APIs and the suggested potential improvements. Finally, the potential for reuse of the dataset through the WiLDSI web app is presented, as well as further potential for tracking genetic resource use in the scientific literature and aspects of quantifying DSI use in the context of benefit-sharing discussions under the CBD."

Comment 2.5:
There is no need to explain the FAIR data principle

Response 2.5:
In the context of the CBD negotiations, the role of NSD/DSI garnered immense interest and raised concern across the international scientific community. This was the background for this data note. The intention of the mentioned sentence with reference to FAIR was meant to be a transition to the global benefit of free and open use of DSI as demonstrated particularly in science. We refrained from introducing the FAIR criteria. Rather we intended to illustrate the emerging tension field of open science and FAIR as synonym and benefit sharing discussion.

Comment 2.6:
Using Microsoft Academic dataset can assist in mapping between author to a unique id

Response 2.6:
Thanks for the important suggestion. The ambiguous identification of authors and their affiliation is of importance. Thus, we included this aspect in the quality discussion of the manuscript. As follow-up to response 2.2 and extension to response 1.1 for reviewer 1, we agree that Microsoft Academic is a powerful service, that could assist in mapping between author to a unique ID, like ORCID. Nevertheless, we would have needed to purchase a commercial license to use the service in a programmatic way in the data extraction pipeline and to publish the results. As noted above, the aim of the manuscript was to apply the open public tools available from ePMC and ENA to ensure a broad reproducibility of the presented DSI quantification pipeline in context of the policy discussions.

Comment 2.7:
Mapping between an institute or location to a country can be more accurately done by utilizing geolocation code packages, such as geopy

Response 2.7:
Thanks to refeer by this comment to the importance of having consistently geolocated authors and NSD material orign. In fact 15% of all ENA sequences comprise a country tag as provenance in terms of international law and in context of the data notes objective. In respect to the potential envoronmental context of the "pick-up" location of bio material, 23% of them annotated by specific geo-coordinates, which could be used to validate the material provenance of each ENA deposited sequence data. But in respect to the background of this paper it seamed to be sufficient to considered the country tag only.
In case of scientific literature, the use of geolocation to validate or curate authors

affiliation, if unique institutes identifiers, for example by ROR identifier, would be broadly available. An indirect geo location of authors affiliation by ROR use in authors ORCID record would be a further source for the near future. But currently, a minor fraction of all authors/paper pairs are annotated by an ORCID. Furthermore, how much of these ORCIDs are maintained consistently with authors affiliation history is unknown.

Answers to comment of reviewer #3
I have reviewed this manuscript with integrity, but I'm a little confused about it because I usually use NCBI PubMed/GenBank data. If my points are off the mark, please point them out.

Comment 3.1:
In NCBI PubMed, the nucleotide sequence entries referenced in the article are listed in PubMed data as external DB links (although not perfect), and by extracting these, the relationship between the PubMed and Nucleotide entries can be extracted. The NCBI website also provides these links from Nucleotide in the Related information section (e.g. https://pubmed.ncbi.nlm.nih.gov/19193256/). I found that the ePMC website also has a link in the Data section for nucleotide sequence entries referenced in the paper (e.g., https://europepmc.org/article/MED/19193256). Do you use any of these external links in ePMC data in this work? I think it is very difficult to extract nucleotide IDs by text mining, especially since Nucleotide sequence IDs are not in a fixed format. I think these links will be a great help in doing text mining.

Response 3.1:
Thanks for the comment. Indeed, we also use the one extended version of the pipeline used in ePMC to identify ENA accessions, rather than the pipeline from NCBI Genbank. However, the goal of the study was not so primary as to extract all links. Rather, the data integration pipeline presented was intended to achieve a good balance between recall and precision with respect to the data integration scenario at hand. This is because the underlying literature as well as the sequence data are subject to dynamics and the citation quality of the NSD are very heterogeneous, as discussed in the "Data Validation" section. Therefore, the initial focus for this dataset was on a quantitative assessment of DSI use under manual quality review.
This resulted in this first integrated quality-checked dataset, which is intended to be published in conjunction with a web application for its exploratory evaluation.
However, we agree with the reviewer, have the potential for further work already in the manuscript, and will take up these points in a follow-up project that has already been approved.

Comment 3.2:
2. In NCBI PubMed, MeSH keywords are assigned to each article for indexing the literature. MeSH keywords also include country keywords (e.g. https://pubmed.ncbi.nlm.nih.gov/19193256/). In ePMC Is it possible to use keywords like MeSH in ePMC? Do you have any opinions about using such country keywords?

Response 3.2:
Thanks for pointing this out. Indeed, some primary publications on NSD annotate the material or origin as a keyword in the corresponding publication in addition to the Country tag in ENA for the deposited sequences. In this particular case, Kenya is also consistently annotated to the 92 ENA sequences linked to this primary publication. However, a consistency analysis between keywords in primary publications, and the country tag in the original ENA was not the objective of this work.

Comment 3.3:
I found some great statistics and visualizations of this data on the site the authors provide about it. I would be happy to show these in this manuscript as a result of this work, but please follow the journal's policies and precedents.

Response 3.3:
Thanks for this. Nevertheless, we announced to the editorial board a companion paper that will more deeply elaborate and interpret the charts. This will be linked to this data note. Furthermore, the charts in the Web application have been updated and include

Powered by Editorial Manager® and ProduXion Manager® from Aries Systems Corporation

	more detail to the visualised quantification in respect to the DSI discussions under the Convention on Biological Diversity. In concrete the charts were consolidated and a scatter plot in panel 6 - "Network Graph" was added that visualise details of the global relationship between providing and using of DSI. The charts in panel 2 - "country use of DSI" and panel 3 - "World Maps" are updated and count all publications instead of only secondary publications. We updated the charts in both panels because it's the same data but visualised in different types of graphs. Moreover, a new graph was added in panel 5 - "coalition collaboration" which displays the DSI use in all publications from the different economic groups. This is now the first graph on this page. We also moved chart 5.6 - "BRICS-G77-OECD consumption/contribution" to the fourth position. In panel 4 - "hemisphere collaboration" graph 4.4 - "Distribution of publications using DSI with authors of n-different countries" was deleted.<br><br>Comment 3.4:<br>Do the authors think that users should reuse the created data for this product? Or is it recommended that users create their own data using the creation program? If the former, what is your plan for the frequency of updating the data?<br><br>Response 3.4:<br>We hope that readers will do both: some will explore the data using the web app and others will re-run the analysis on future or otherwise expanded datasets. Regarding data updates, please refer to response 3.1. The aforementioned follow-up project will work on a data update pipeline, with the goal of updating the data every six months.<br><br>Comment 3.5:<br>In Figure 1, I felt that it would be easier for the reader to understand if I emphasized (by changing the line or fill of the box) whether the data in each step is Nucleotide data, literature data, or ID pairs extracted from those data.<br><br>Response 3.5:<br>Indeed, the layout of Figure 1 aimed to illustrate the complex data extraction and integration workflow in a sketchy manner. The current highlighting and sectioning into a, b and c intended to reflect and logically group the processing steps. The suggestion to emphasize the data domain would surely improve illustrative quality. Thanks for this hint, which we followed and reshaped the Figure 1 towards a more visually clear tagging of nucleotide and literature data.
**Additional Information:**	
**Question**	**Response**
Are you submitting this manuscript to a special series or article collection?	No
**Experimental design and statistics**<br><br>Full details of the experimental design and statistical methods used should be given in the Methods section, as detailed in our Minimum Standards Reporting Checklist. Information essential to interpreting the data presented should be made available in the figure legends.<br><br>Have you included all the information requested in your manuscript?	Yes
**Resources**	Yes

A description of all resources used, including antibodies, cell lines, animals and software tools, with enough information to allow them to be uniquely identified, should be included in the Methods section. Authors are strongly encouraged to cite Research Resource Identifiers (RRIDs) for antibodies, model organisms and tools, where possible.<br><br>Have you included the information requested as detailed in our Minimum Standards Reporting Checklist?	
**Availability of data and materials**<br><br>All datasets and code on which the conclusions of the paper rely must be either included in your submission or deposited in publicly available repositories (where available and ethically appropriate), referencing such data using a unique identifier in the references and in the "Availability of Data and Materials" section of your manuscript.<br><br>Have you have met the above requirement as detailed in our Minimum Standards Reporting Checklist?	Yes

Powered by Editorial Manager® and ProduXion Manager® from Aries Systems Corporation

Manuscript
Click here to access/download;Manuscript;Manuscript_revised.docx

Quantitative monitoring of nucleotide sequence data from genetic resources in context of their citation in the scientific literature

Matthias Lange[1] [0000-0002-4316-078X], Blaise T.F. Alako[2] [0000-0001-6859-4421], Guy Cochrane[2] [0000-0001-7954-7057], Mehmood Ghaffar[1] [0000-0001-5062-5429], Martin Mascher[1,3] [0000-0001-6373-6013], Pia-Katharina Habekost[1,4] [0000-0002-3461-0881], Upneet Hillebrand[5] [0000-0002-3312-4013], Uwe Scholz[1] [0000-0001-6113-3518], Florian Zunder[1,4] [0000-0001-6495-8611], Jens Freitag[1] [0000-0001-6905-5497], Amber Hartman Scholz[5] [0000-0002-3461-0881]

[1]Leibniz Institute of Plant Genetics and Crop Plant Research, Germany

[2]European Molecular Biology Laboratory, European Bioinformatics Institute, Wellcome Genome Campus, Hinxton, United Kingdom

[3]German Centre for Integrative Biodiversity Research (iDiv) Halle-Jena-Leipzig, Leipzig, Germany

[4]Harz University of Applied Sciences, Germany

[5]Leibniz Institute DSMZ-German Collection of Microorganisms and Cell Cultures, Germany

Abstract

Background

Linking nucleotide sequence data (NSD) to scientific publication citations can enhance understanding of NSDs provenance, scientific use, and re-use in the community. By connecting publications with NSD records, NSD geographical provenance information, and author geographical information, it becomes possible to assess the contribution of NSD to infer trends in scientific knowledge gain at the global level.

Findings

For this data note, we extracted and linked records from the European Nucleotide Archive to citations in open-access publications aggregated at Europe PubMed Central. A total of 8,464,292 ENA accessions with geographical provenance information were associated with publications. We conducted a data quality review to uncover potential issues in publication citation information extraction and author affiliation tagging and developed and implemented best-practice recommendations for citation extraction. Flat data tables and an data warehouse with an interactive web application were constructed to enable ad hoc exploration of NSD use and summary statistics.

Conclusions

The extraction and linking of NSD with associated publication citations enables transparency. The quality review contributes to enhanced text mining methods for identifier extraction and use. Furthermore, the global provision and use of NSD enables scientists around the world to join literature and sequence databases in a multidimensional fashion. As a concrete use case, statistics of country clusters were visualized with respect to NSD access in the context of discussions around digital sequence information under the United Nations Convention on Biological Diversity.

Keywords

data citation, nucleotide sequence data, Europe PMC, European Nucleotide Archive, text mining, Convention on Biological Diversity, digital sequence information

Data Description

Nucleotide Sequence Data (NSD) plays a fundamental role in biological research ranging from public health and medical applications to understanding the molecular basis of life and evolution, such as how genes (mis)function in disease mechanisms [1], insights into ecosystem functioning and biodiversity conservation, and to assist in breeding new plant variety and animal breeds enabling food security and sustainability [2]. Scientifically, NSD plays a major role for mechanistic modeling of species evolution [3], genotype – phenotype correlation [4], to identify and mitigate risks to species, track their illegal trade, identify the geographical origin of products, and plan conservation management strategies [5].

These applications demonstrate the wide value of NSD use and application and have triggered political debate about benefit sharing from genetic resources (GR). Under the Convention on Biological Diversity (CBD) and the Nagoya Protocol [6] as well as the International Treaty on Plant Genetic Resources for Food and Agriculture (ITPGRFA) the topic of "digital sequence information" (DSI) has garnered immense interest and raised concern across the international scientific community. Discussions have focused on the use of NSD from GRs, as DSI is an undefined and non-scientific term. Due to the exponential growth of public sequence and downstream databases [7] many parties are concerned that insufficient benefit-sharing is taking place. Datasets such as this one provides an opportunity for evidence-based policymaking to analyze global trends in NSD provision and use as well as other science policy fields, including scientific strategic development and internationalization.

With this in the background, this Data Note subsequently presents the context of the dataset for quantified NSD use. As such, the method for extracting NSD citations from the scientific literature is described, as well as the technical details of constructing the data warehouse. The Data Validation and Quality Control section discusses the refinement process of the data

extraction pipeline and potential shortcomings arising from the available data quality, the provided APIs and the suggested potential improvements. Finally, the potential for reuse of the dataset through the WiLDSI web app is presented, as well as further potential for tracking genetic resource use in the scientific literature and aspects of quantifying DSI use in the context of benefit-sharing discussions under the CBD.

Context

FAIR (findable, accessible, interoperable, re-usable) data principles as defined in 2016 in the FAIR Guiding Principles for scientific data management and stewardship [8] guide the design of open data sharing infrastructures as an enabling technology for economic and scientific progress. Data sharing principles have been implemented at national and international levels. For example, the German Federal Ministry of Education and Research (BMBF) has funded an interdisciplinary project called "Science-based approaches for Digital Sequence Information" (WiLDSI) [9] which aims to (i) raise awareness and involve the international scientific community into the debate and decision making process surrounding DSI, (ii) to identify and elaborate scenarios for open access to the NSD and (iii) to establish fair and sustainable benefit sharing.

In this context, transparent quantitative measures of NSD citation and re-use can inform decision-making processes surrounding the design of data sharing infrastructure, awarding scientific "credit" or political acknowledgment, or addressing needs of commercial users [10]. Data citation has received increased attention from publishers, funding agencies and infrastructure providers [11] [12] in recent years. However, best practices for NSD citation are still lacking and those developed for scientific publications cannot be readily transferred. This

is true especially for NSD, which is hosted by the core data infrastructure, the International Nucleotide Sequence Database Collaboration (INSDC) [13]. The European Nucleotide Archive (ENA) [14] and Europe PubMed Central (ePMC) [15] are, respectively, the European partners in INSDC and a repository of open access articles. Both have a long tradition of handling open data and document the heterogeneous quality of author's data citation practices [16]. ePMC listed publications generally employ text-embedded ENA identifiers, like accession numbers, project accessions, or study accessions.

Methods

Figure 1 shows the extraction of ENA citations performed in three phases. First, ENA accessions and project accession numbers were extracted. Literature citations listed directly in the ENA entry were extracted in parallel and are called herd "primary publications". Next, we retrieved scientific papers that referred to these accession IDs via a full text search using the ePMC REST API [17]. These publications we labelled "secondary publications". Finally, the extracted references and associated citation information were organized into six tables ~~(Figure 2)~~ and imported into a data warehouse. Its schema, as documented in Figure 2, shows the referential dependencies, foreign keys, and primary key attributes. This is intended to support advanced use of data exploration functionality in the web application. It is also intended to reduce the barrier for users to reuse the raw table dumps for downstream analysis.

<Figure 1 (replaced in manuscript revision)>

Figure 1. Visualization of data processing performed to extract, filter and join ENA and ePMC data sets. ENA records are parsed (A1) and filtered for valid country tag and fed into ePMC RestFul API to extract matching secondary publication (B1) by ENA accession or project accession numbers. Primary publications are linked by ENA record (A2) to the DOI, PMCID or PMID. The resulting data sets are normalized as tables ENA_SEQUENCES, PMC_REFERENCES and loaded into the data warehouse (A3, B2) alongside a curated list of world's countries in table COUNTRIES and economics groups in table COUNTRY2GRP (C1). SQL queries (C2) are applied to generate charts and reports in the Web application.

Five classes of citation pattern are used for ePMC publication as ENA identifiers where used: single accession number using word separation characters, e.g. hyphens, brackets, quotation marks; range notation of referenced accessions, text embedded enumeration, lists in supplement material or even embedded into figure bitmaps. The data extraction process from EMBL-ENA and ePMC was executed by Perl and Python scripts. The starting point of the data extraction process is the EMBL-ENA flat file dump of release 143, which was obtained from the EMB-ENA FTP server and comprised 263,421,789 records. Next, all non-WGS ENA records were parsed to compile a relevant set of attributes for the table "ENA_SEQUENCES". A total of 17,820,136 ENA accessions with valid country tags (i.e., the /country field in the ENA entry, comprising 15% of all records) were included. Next, using the ePMC REST API, these ENA accessions were scanned in 36.7 million full text articles accessible via PubMed. Due to performance reasons, this text tokenization was executed on site at EBI in a compute cluster environment. From the resultant publications those were select that have valid author

country information and that either (b) cite an ENA sequence as a secondary publication[1] or (b) that are cited by ENA record as primary publication[2]. The publications matching these criteria were compiled into the table “PMC_REFERENCES”. In detail, 5,935,939 sequences cite 61,608 publications and 2,716,000 sequences are cited by 91,234 publications respectively. All scripts used in our analyses are available in GitHub [18].

<Figure 2>

Figure 2. Table schema of the WiLDSI data warehouse - The table ENA_SEQUENCES comprise metadata of a sequence stored in EBI ENA database. The attributes accession and project accession are used to join secondary literature that cite sequences. The attribute country refers to the country table to resolve and group country tagged ENA sequences. The table PMC_REFERENCES consists of all ePMC published papers either referencing a ENA sequence by accession or project accession and references from ENA records as primary publication by either a DOI, PMID or PMCID.

The table "COUNTRY" was compiled and curated from UN state membership [19]. It comprises the three kinds of ISO-3166-1-codes, the official name (e.g. United Kingdom of Great Britain and Northern Ireland), a short version of the state name (e.g United Kingdom), commonly used names (e.g. Great Britain), and continent assignments. This table allows mapping from partly ambiguous country affiliation used in papers to the actual country designations recognized under international law. In particular, provinces or (partially) autonomous areas, such as Taiwan or West Sahara, are mapped to the legally responsible UN

[1] designated as secondary in CBD context
[2] designated as primary in CBD context

state party. Furthermore, several Ocean areas, for example “Bismarck Sea” or “East China Sea” are grouped together under the “Ocean” label along with more standard fields such as “Atlantic Ocean”[3]. The assignment to economic groups is stored in table “COUNTRY2GRP”. Here a 2-letter ISO code is assigned to rough economic groups OECD (Organization for Economic Cooperation and Development), BRICS (Brazil, Russia, India, China, South Africa), and G77 (representative of developing economies). In order to visualize countries in a world map, we used the table “GEOLOCATION” comprising the coordinates of the centroid of each country.

The tables are provided for download as CSV files (see section “Data Availability”). To support on-line analytical data processing and convenient data access, a data warehouse was built based on the extracted data set. To scale appropriately, an ORACLE Enterprise RDBMS was applied. It enables analytical real-time SQL queries over millions of data points by means of in-memory materialized views, vectorization and columnar storage. This in turn was the basis to guarantee an appropriate user experience for the subsequently presented web application, which provides interactive, on-line calculation of metrics from NSD citations to various filters and data groupings, to drill-down chart and link data to the original records in ENA and ePMC.

[3] “Ocean” is not equivalent to international waters under the United Nations Convention of the Law of the Seas (UNCLOS, where marine genetic resources and benefit-sharing are beeng discussed) but is in this context simply a consolidated term representing sampling in the marine environment.

Data validation and quality control

In order to assess the reliability of the extracted ePMC to ENA references, potential quality issues were evaluated by plausibility scans across data warehouse tables, including inspection of 20 randomly sampled papers performed by domain experts from IPK's sequence submission service team. We also took into account review articles on the use of data identifiers in life science literature [20] [21]. Finally, we applied the Dimensions text mining tool [22] to cross-check the sensitivity of ePMC API in respect of recall and sensitivity, e.g., to find false negative hits such as published articles that reference ENA sequences but which were not found by the ePMC REST API.

Country names

Country name had to match records in the country table. Here we found some obsolete or ambiguous country names, like Montenegro or West Sahara and historic country names, like Soviet Union, which cannot be assigned uniquely to current UN states. Ambiguous country names were resolved manually and reverted to synonyms in the country table (e.g. Cote d'Ivoire to Ivory Coast amongst others). ENA or PMC records with obsolete country tags were kept in the data set but ignored for summary statistics queries and excluded in below quality check.

Retrieval of referenced IDs

Next, reference consistency among the extracted ENA and ePMC records were checked by ETL test runs. This resulted in a preliminary data warehouse instance, which allowed SQL-based plausibility checks. Such as the count unique paper identifiers, ENA accessions, and country tags or count the number of records in preliminary joining ENA and ePMC records over different combination of PMID, PMCID and DOI. We specifically checked whether ENA

records refer to a valid country. For 217.40 ENA records out of 18.034.192 was it not the case, e.g. country tag “Western Sahara” (ENA accession HM034625) or empty country tag (ENA accession KM654101). Those could in some cases resolved by manual addition of synonyms to the country table that reflects the current valid UN agreed assignments. In case of empty country tags, we found some were annotated with geographical location. However, we left them empty to not intransparently change primary data.

Further we checked, if primary papers referenced in an ENA record exist in the ePMC databases. Here, a total of 6,753,891 ENA records refer either by DOI, PMID, or PMCID to 351,119 ePMC records, i.e. some use DOI only, some DOI and PMID etc. Conversely, there are 9,589,900 ENA records without any primary literature reference. Furthermore, we confirmed that ePMC records, which cite secondary ENA accession or project numbers can be resolved to records in ENA. We found 189,581 ePMC records that reference 2,801,072 ENA records by either accession or project accession number. A potential issue in the context of using an identifier to cite ENA records is that authors sometimes use ENA study identifiers or even BioSample IDs. However, our pipeline considers ENA accession and project accession only.

Author identification

The combination of first and last names are not unique identifiers for human beings. ORCIDs provide unique identifiers for authors and are on their way to becoming compulsory for publications. Existing articles, however, are only occasionally associated with ORCID. Another potential issue is that it is possible to register multiple ORCID for one person. Identifying authors as a concatenation of author name and affiliations is error prone [23, 24]. Therefore, author information was retained in the tables but not used for statistical analysis.

Range notation

Scientific publication may use ambiguous range notation to cite ENA accessions. As illustrated in Figure 3, hyphen as range notation aggregate a sequence of ENA accessions. Here, the authors assume an ordered sequence of accession numbers and it is interpreted as such by human readers, but is not recognized by programmatic text mining. Thus, in the data extraction used here, a potentially high number of ENA accessions are missed and the dataset is an underestimate of the number of referenced DSI. This analysis is intended to support future work to address these shortcomings.

<Figure 3>

Figure 3: Example of range notation for ENA accession references. Within the selected part of publication with DOI: 10.3201/eid1502.080851 the actual number of cited ENA accessions is 35, but ePMC API matched 8 only.

Access restrictions

Only open access publications were available for high-throughput text mining. In order to efficiently process 18 million ENA accession numbers the ePMC REST API at EBI's local compute infrastructure was used. This causes a potential loss of recall in comparison to a broad and integrative use of further state-of-the-art literature mining services that include articles behind paywalls. In order to get an estimation of potential missed DSI citation, we used alternative tools that cover patent and closed access publications. We applied the commercial "Dimensions" [22] and the free "Lens.org" [25] search tools, which include patent and restricted-access publication, to compare recall performance for 20 randomly selected ENA accessions. To work with a comparable corpus, this evaluation was performed within 4 weeks of the ePMC based text mining run. The results are compiled in Table 1. Specific hits to one of three approaches, ePMC, Lens, dimensions were observed. This is likely due to the

larger corpora of Dimensions and Lens. For example, ENA accession AB076935 was linked to 3 public and 3 closed access publications by Dimensions, whereas ePMC did not report any matching publication. Differences in file format may explain some of the differences. There are cases where the PDF rendered articles differ from ePMC rendered HTML versions, so that the PDF versions can contain more ENA accession numbers than HTML versions in ePMC. We did not aim for an in-depth analysis for literature search tools, but our cursory overview supports the notion that a substantial number of publications relevant to NSD may be behind paywalls, scanning PDF encoded manuscripts, the use sophisticated text mining methods and integration of commercial text mining software could improve the recall and precision of NDS citation in texts as well. However, as the spirit of the project in which this analysis took place, with a heavy emphasis on open access and ENA API, we continued our analyses with the open dataset.

ENA ACCESSION	hits in in ePMC	hits in Dimensions	hits in Lens	overlap Dimensions and ePMC	overlap Lens and ePMC
AB076935	0	6	0	0	0
AB076941	0	1	0	0	0
EU257628	3	5	0	2	0
AB326609	0	1	0	0	0
AM262332	0	2	0	0	0
EU575854	1	1	0	1	0
CP039348	0	1	0	0	0
DQ410599	1	1	0	1	0
EU293114	12	19	1	6	1
AY924392	10	7	2	6	2

EF607913	0	1	0	0	0
AY768827	0	1	0	0	0

Table 1. Comparison of ENA accession number query APIs performance of APIs of EBI ePMC, Dimensions[4] and Lens[5].

Re-use potential

To enable the further exploration of the data set, a web application was developed and is publicly accessible at https://wildsi.ipk-gatersleben.de. We focused especially on understanding NSD/DSI usage in the context of fair and equal benefit sharing. More generally, the web interface illustrated in Figure 4 enables the interactive exploration of DSI use in science by a features text search, data aggregation across the data warehouse and crosslinking to the original ENA records and ePMC records. It enables further complex filtering, grouping as well as visualition as charts, world map projects, and network diagrams. Based on the use cases provided in this CBD context, basic questions regarding DSI usage are visualized in different relationships to answer questions such as: Which countries use DSI? Which countries (groups) contributed DSI? Are there countries that use DSI but do not contribute DSI? This is implemented by four classes of use cases: *general overview of DSI, per country use of DSI, collaborative use in economic and hemisphere groups, world map projection, DSI citation network*.

<Figure 4>

[4] Our queries used this URL pattern: https://app.dimensions.ai/discover/publication?search_text=AY924392&search_type=kws&search_field=full_search

[5] Our queries used this URL pattern: https://www.lens.org/lens/scholar/search/results?q=AY924392&preview=true

Figure 4. Screenshots of the WiLDSI Web Application. It consists of pages for (A) detailed data reports with integrated (B) drill down to original sources, (C) charts of DSI usage scenarios, (D) per country DSI use and contribution etc.

Another re-use scenario is to document the flow of DSI associated with genebank resources across the scientific value chain from seed storage to genetic analysis. A prominent example is the role of molecular passport data as an instance of DSI to characterize plant genetic resources (PGR). Genebank genomics is an emerging research field aiming at using high-throughput sequencing to characterize the genetic diversity in entire genebank collections [26]. Recently, marker profiles from reduced representation sequencing data were reported for more than 20,000 accessions of the German Genebank [27]. Whole-genome shotgun sequencing has been used to characterize the genome of 3,000 rice accessions at the International Rice Research Institute [28]. The approach provides a so-called molecular passport that enables tracking the identity of accessions, identifying redundancies and cross-link international genebanks [29]. For these reasons, molecular passport data is poised to become an essential component of working with PGR in research and breeding contexts. Documenting the use of DSI associated with PGR would help genebank managers and administrators of genebank information systems monitor the use of their accessions in international research efforts and help justify the tremendous effort put into the maintenance and characterization of PGR in global genebanks. Documenting DSI could also help national authorities to enforce access and benefit-sharing schemes of the Nagoya protocol. The present enquiry into the status of DSI in public sequence archives has shown that sequence information of PGR is abundant, but tracing it back to the gene bank holdings it derives from, can be challenging. In the coming years, gene bank managers, genome researchers and bioinformaticians should develop and enshrine standards and protocols for

linking DSI in archives such as EMBL-ENA to gene bank information systems and meta-databases such as EURISCO [30]. Work in this direction is underway in the EU-funded project AGENT [31].

DSI and their free accessibility are essential for all areas of the life sciences, including biodiversity research, food security, human health, biological conservation and many other disciplines or research areas. Some countries contributing DSI fear that direct access to the increasing amount of freely available sequence information may undermine benefit sharing schemes for genetic resources. A use of this data set supports evidence-based decision making in the context of international policy processes as well as global scale investigations into scientific use and re-use of NSD datasets and sub-disciplines thereof. Indeed, this article is intended as a companion paper for a timely publication on the policy implications of NSD (re-)use for DSI access and benefit-sharing discussions under the CBD in this issue [32].

For future studies, the examples above could be complemented by more detailed use cases including finer-grained groupings for data aggregation such as separation of genera, species and time ranges of publications. In combination with additional text classification techniques [33], it may be possible to cluster by research topics, e.g. considering only citations in paper involving, say, COVID-19 or plant pathogen resistance.

Availability of source code and requirements

Project name: WiLDSI

Project home page: https://wildsi.ipk-gatersleben.de

Operating system(s): LINUX

Programming language: Oracle Application Express, Perl, Python3

Other requirements: HTML5 compatible web browser

License: GNU General Public License v3.0

All scripts used for data extraction are available from GitHub https://github.com/alakob/sequence-literature.

Data Availability

The charts, maps, and data tables are available in an interactive web application at http://wildsi.ipk-gatersleben.de. The data tables are published as CSV files in the e!DAL-PGP repository [34] under the DOI 10.5447/ipk/2021/8. The SQL queries implementing the use cases are linked and documented alongside each chart within the web application.

List of abbreviations

CBD: Convention on Biological Diversity; ITPGRFA, International Treaty for Plant Genetic Resources for Food and Agriculture DOI: Document Object Identifier; EMBL: European Molecular Biology Laboratory; ENA: European Nucleotide Archive; ePMC: Europe PubMed Central; DSI: Digital Sequence Information - synonym for nucleotide sequence data in international policy circles; GR: Genetic Resources; INSDC: Nucleotide Sequence Database Collaboration; NSD: Nucleotide Sequence Data - synonym to DSI in a technical and database context; ORCID: Open Researcher and Contributor ID; PGR: Plant Genetic Resources; WiLDSI: German: "wissenschaftsbasierte Lösungsansätze für digitale Sequenzinformation", English translation: Science-based Approaches for Digital Sequence Information

Ethics approval and consent to participate

Not applicable.

Consent for publication

Not applicable.

Competing interests

The author(s) declare that they have no competing interests.

Funding

This work was supported by the German Federal Ministry of Education and Research (BMBF) in the frame of the project “WiLDSI: Wissensbasierte Lösungsansätze für Digitale Sequenzinformation” (FKZ 031B0862) and IPK Gatersleben core funding.

Authors' contributions

Conceptualization: A.S., J.F., G.C., M.L.

Software: M.G., B.A., M.L., P.H., F. Z.

Data curation: U.H., J.F., M.L.

Investigation: A.S., J.F., U.H.

Supervision: M.L., A.S., J.F., G.C.

Writing original draft: M.G., M.L., M.M., A.S.

Writing review and editing: All authors

Funding acquisition: A.S., U.S.

Acknowledgements

We thank H. Miehe, M. Oppermann and T. Münch for technical support and hosting the web application. We also thank the thousands of authors that generated the data and publications

analyzed here especially to those committed to open access which enables this global overview.

References

[1] E. R. Mardis, ‘A decade’s perspective on DNA sequencing technology’, *Nature*, vol. 470, no. 7333, pp. 198–203, Feb. 2011, doi: 10.1038/nature09796.

[2] ‘Digital sequence information | Commission on Genetic Resources for Food and Agriculture | Food and Agriculture Organization of the United Nations’. http://www.fao.org/cgrfa/topics/digital-sequence-information/en.

[3] P. A. Romero and F. H. Arnold, ‘Exploring protein fitness landscapes by directed evolution’, *Nat. Rev. Mol. Cell Biol.*, vol. 10, no. 12, pp. 866–876, Dec. 2009, doi: 10.1038/nrm2805.

[4] M. D. Ritchie, E. R. Holzinger, R. Li, S. A. Pendergrass, and D. Kim, ‘Methods of integrating data to uncover genotype–phenotype interactions’, *Nat. Rev. Genet.*, vol. 16, no. 2, pp. 85–97, Feb. 2015, doi: 10.1038/nrg3868.

[5] F. W. Allendorf, P. A. Hohenlohe, and G. Luikart, ‘Genomics and the future of conservation genetics’, *Nat. Rev. Genet.*, vol. 11, no. 10, pp. 697–709, Oct. 2010, doi: 10.1038/nrg2844.

[6] ‘About the Nagoya Protocol’. https://www.cbd.int/abs/about/.

[7] A. Szalay and J. Gray, ‘Science in an exponential world’, *Nature*, vol. 440, no. 7083, pp. 413–414, Mar. 2006, doi: 10.1038/440413a.

[8] M. D. Wilkinson *et al.*, ‘The FAIR Guiding Principles for scientific data management and stewardship’, *Sci. Data*, vol. 3, no. 1, p. 160018, Dec. 2016, doi: 10.1038/sdata.2016.18.

[9] 'German Collection of Microorganisms and Cell Cultures GmbH: Digital Sequence Information'. https://www.dsmz.de/collection/nagoya-protocol/digital-sequence-information (accessed Mar. 31, 2021).

[10] N. Robinson-García, E. Jiménez-Contreras, and D. Torres-Salinas, 'Analyzing data citation practices using the data citation index', *J. Assoc. Inf. Sci. Technol.*, vol. 67, no. 12, pp. 2964–2975, Dec. 2016, doi: 10.1002/asi.23529.

[11] G. Silvello, 'Theory and practice of data citation', *J. Assoc. Inf. Sci. Technol.*, vol. 69, no. 1, pp. 6–20, Jan. 2018, doi: 10.1002/asi.23917.

[12] H. Cousijn *et al.*, 'A data citation roadmap for scientific publishers', *Sci. Data*, vol. 5, no. 1, p. 180259, Dec. 2018, doi: 10.1038/sdata.2018.259.

[13] M. Arita, I. Karsch-Mizrachi, and G. Cochrane, 'The international nucleotide sequence database collaboration', *Nucleic Acids Res.*, vol. 49, no. D1, pp. D121–D124, Jan. 2021, doi: 10.1093/nar/gkaa967.

[14] P. W. Harrison *et al.*, 'The European Nucleotide Archive in 2020', *Nucleic Acids Res.*, vol. 49, no. D1, pp. D82–D85, Jan. 2021, doi: 10.1093/nar/gkaa1028.

[15] M. Levchenko *et al.*, 'Europe PMC in 2017', *Nucleic Acids Res.*, vol. 46, no. D1, pp. D1254–D1260, Jan. 2018, doi: 10.1093/nar/gkx1005.

[16] Ş. Kafkas, J.-H. Kim, and J. R. McEntyre, 'Database Citation in Full Text Biomedical Articles', *PLoS ONE*, vol. 8, no. 5, p. e63184, May 2013, doi: 10.1371/journal.pone.0063184.

[17] 'Europe PMC - Developer resources', *Europe PMC*, Feb. 08, 2021. https://europepmc.org/developers.

[18] Alko, Blaise, *Exploratory Analysis and Predictive Modelling of sequences citation in the literature.* https://github.com/alakob/sequence-literature.

[19] U. Nations, ‘UN Member States’, *United Nations Member States*. https://www.un.org/en/about-us/member-states (accessed Mar. 31, 2021).

[20] Ş. Kafkas, J.-H. Kim, X. Pi, and J. R. McEntyre, ‘Database citation in supplementary data linked to Europe PubMed Central full text biomedical articles’, *J. Biomed. Semant.*, vol. 6, no. 1, p. 1, 2015, doi: 10.1186/2041-1480-6-1.

[21] J. A. McMurry *et al.*, ‘Identifiers for the 21st century: How to design, provision, and reuse persistent identifiers to maximize utility and impact of life science data’, *PLOS Biol.*, vol. 15, no. 6, p. e2001414, Jun. 2017, doi: 10.1371/journal.pbio.2001414.

[22] D. W. Hook, S. J. Porter, and C. Herzog, ‘Dimensions: Building Context for Search and Evaluation’, *Front. Res. Metr. Anal.*, vol. 3, p. 23, Aug. 2018, doi: 10.3389/frma.2018.00023.

[23] P. Sebo, S. de Lucia, and N. Vernaz, ‘Accuracy of PubMed-based author lists of publications and use of author identifiers to address author name ambiguity: a cross-sectional study’, *Scientometrics*, Mar. 2021, doi: 10.1007/s11192-020-03845-3.

[24] Baglioni, M., Mannocci, A., Manghi, P., Atzori, C., Bardi, A., and Bruzzo, S. L. Reflections on the misuses of ORCID ids. In CEUR Workshop Proceedings of the 17th Italian Research Conference on Digital Libraries, http://CEUR-WS.org. pp. 117–125, 2021.

[25] ‘Results The Lens - Free & Open Patent and Scholarly Search’, *The Lens - Free & Open Patent and Scholarly Search*. https://www.lens.org/lens (accessed Apr. 01, 2021).

[26] M. Mascher, M. Schreiber, U. Scholz, A. Graner, J. C. Reif, and N. Stein, ‘Genebank genomics bridges the gap between the conservation of crop diversity and plant breeding’, *Nat. Genet.*, vol. 51, no. 7, pp. 1076–1081, Jul. 2019, doi: 10.1038/s41588-019-0443-6.

[27] S. G. Milner *et al.*, ‘Genebank genomics highlights the diversity of a global barley collection’, *Nat. Genet.*, Nov. 2018, doi: 10.1038/s41588-018-0266-x.

[28] W. Wang *et al.*, 'Genomic variation in 3,010 diverse accessions of Asian cultivated rice', *Nature*, vol. 557, no. 7703, pp. 43–49, May 2018, doi: 10.1038/s41586-018-0063-9.

[29] X. Yu *et al.*, 'Genomic prediction contributing to a promising global strategy to turbocharge gene banks', *Nat. Plants*, vol. 2, no. 10, p. 16150, Oct. 2016, doi: 10.1038/nplants.2016.150.

[30] S. Weise, M. Oppermann, L. Maggioni, T. van Hintum, and H. Knüpffer, 'EURISCO: The European search catalogue for plant genetic resources', *Nucleic Acids Res.*, vol. 45, no. D1, pp. D1003–D1008, Jan. 2017, doi: 10.1093/nar/gkw755.

[31] 'Activated GEnebank NeTwork (AGENT)', *Global Access to Plant Genetic Resources*. https://www.agent-project.eu (accessed Feb. 23, 2021).

[32] A. Hartman Scholz, M. Lange, P. Habekost, P. Oldham, I. Cancio; G. Cochrane; J. Freitag, 'Myth-busting the provider-user relationship for digital sequence information', GigaScience, 2021, submitted (preprint DOI: 10.1101/2021.08.02.454535), 2021.

[33] J. Chen, U. Scholz, R. Zhou, and M. Lange, 'LAILAPS-QSM: A RESTful API and JAVA library for semantic query suggestions', *PLOS Comput. Biol.*, vol. 14, no. 3, p. e1006058, Mar. 2018, doi: 10.1371/journal.pcbi.1006058.

[34] D. Arend, A. Junker, U. Scholz, D. Schüler, J. Wylie, and M. Lange, 'PGP repository: a plant phenomics and genomics data publication infrastructure', *Database*, vol. 2016, p. baw033, 2016, doi: 10.1093/database/baw033.

Figure 1 (revised)

Click here to access/download;Figure;Figure_1.pdf

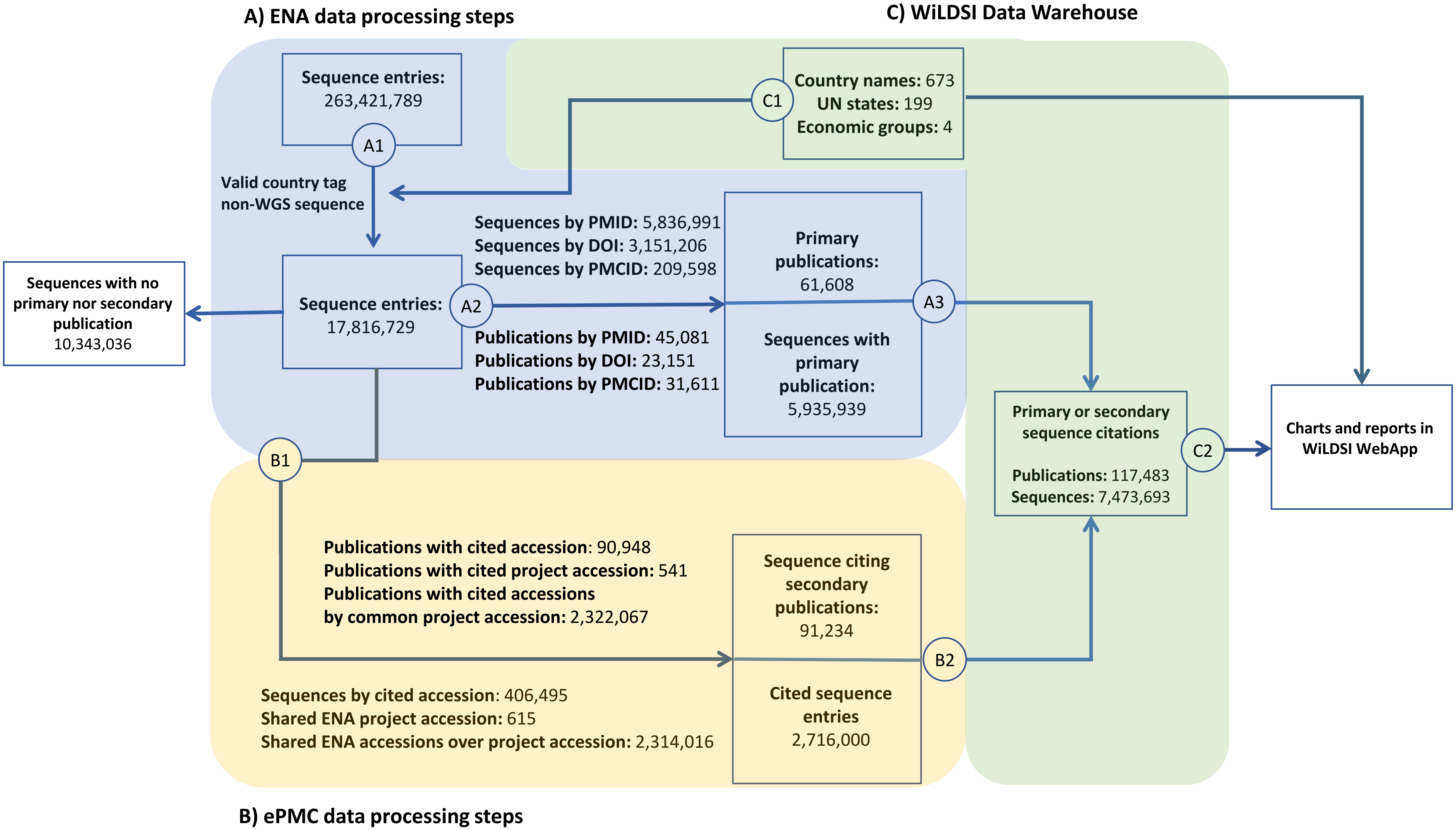

Figure 2 (revised)

Click here to access/download;Figure;Figure_2.png

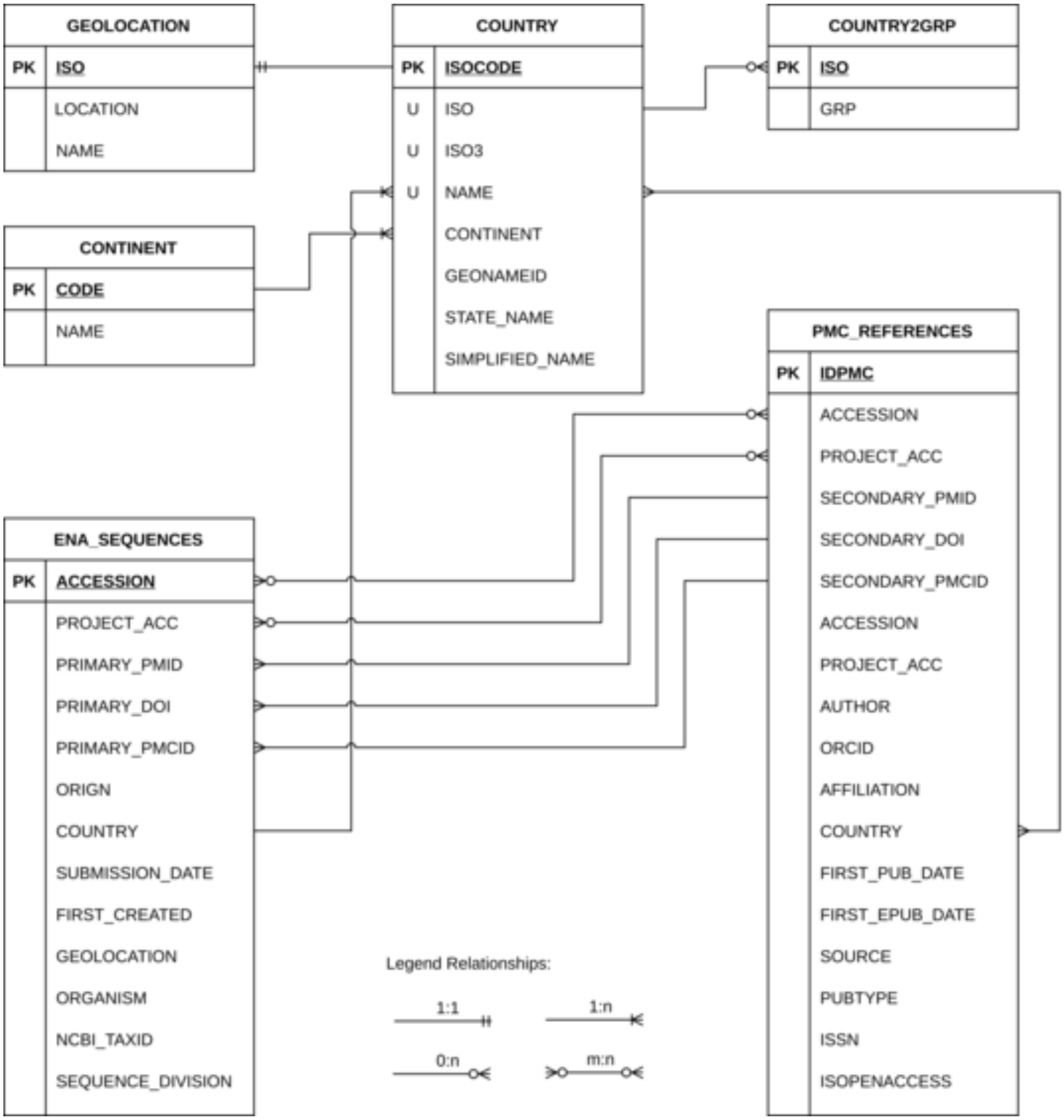

 Click here to access/download;Figure;Figure_3.png

Table 4. Pairwise comparison of full-length nucleotide and amino acid sequences of the medium segment of Kupe virus with other nairoviruses*

Virus	Kupe	Dugbe ArD44313	HAZV	CCHFV
Kupe		61.9	54.7	52.1
Dugbe ArD44313	57.0		53.7	52.5
HAZV	47.7	44.4		50.8
CCHFV	43.0	38.3	41.4	

*Nucleotide identity (%) is shown above the diagonal, and amino acid identity (%) is shown below the diagonal. HAZV, Hazara virus; CCHFV, Crimean-Congo hemorrhagic fever virus.

EU257626, EU816906–EU816909). Results of phylogenetic analysis of the full-length S segment amino acid se-

L fragment alignment overlapping the RDRP core domain (Kupe virus nt 7292–7894) included sequences from 26 DUGV isolates obtained in Kenya in 1999 (GenBank accession nos. EU359010–EU359035), DUGV ArD 44313, and Kupe virus. Results of these comparisons are shown in Table 6. Sequence identities among 5 Kupe virus isolates for this fragment were nt 91.2%–100.0% and aa 98.5%–100.0% (GenBank accession nos. EU257628, EU816898–EU816901).

Discussion

Although little genetic information is available for most viruses in the genus *Nairovirus*, current classification

Figure 4 (revised)

Click here to access/download;Figure;Figure_4.png

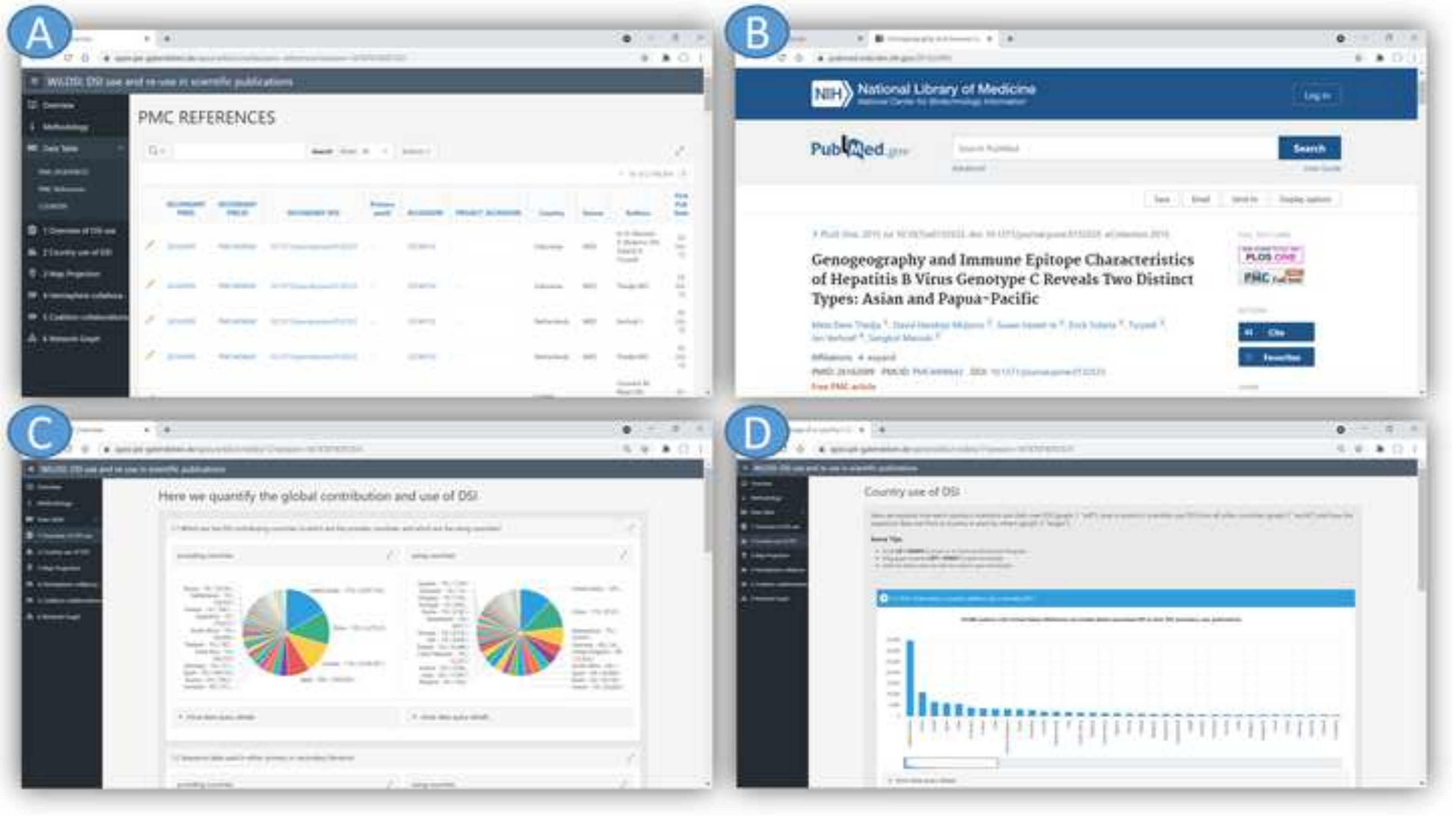

 Click here to access/download;Personal Cover;Cover_letter_and_response_to_reviewer.docx

Dear editors, dear reviewers,

We are very appreciative of the review and comments on our article. In the following letter we answer all questions and provide comments on the intended revisions. All revisions in the manuscript are highlighted in red. To address the editor's suggestion to provide more insight into the implications and analysis of the data sets presented, we have submitted a tandem “policy paper” (your submission identifier GIGA-D-21-00228), published a pre-print in BioRxiv (DOI: 10.1101/2021.08.02.454535) and have added further explanatory text on each of the diagrams in the web application.

The point-by-point response to the reviewers' comments can be found hereafter.

Answers to comment of reviewer #1

Comment 1.1:

Figure 1 is not readable. The sampling process lowered the quality of the image and made the text not readable. Please, use vectorial images (e.g., PDF or EPS). Anyhow, I could understand the process from the descriptive text.

Response 1.1:

Sorry for the low quality of the rendered PDF. The figures’ resolution was increased to 1200 dpi and the transparent background was replaced by white to avoid potential PDF rendering issues.

Comment 1.2:

Figure 2 is readable, but the quality is relatively low. Nevertheless, I do not think this figure is instrumental; it is a simple logical schema of a relational database. Uploading the SQL dump or the SQL schema in an external repository and reference it in the paper would be enough.

Response 1.2:

The image resolution was increased. The detailed referential relationships between the data entities at the attribute level are visualised in the ER diagram. These relationships are important for readers who will go on to use the data set in two ways: first, deeper functionality in the Web app. requires a knowledge of the underlying schema; second, the immediacy of the visualization in the paper will reduce the barrier for users to re-use the raw table dumps for downstream analysis. This intention is now better expressed in the manuscript in the methods section on page 5:

“Its schema, as documented in Figure 2, shows the referential dependencies, foreign keys, and primary key attributes. This is intended to support advanced use of data exploration functionality in the web application. It is also intended to reduce the barrier for users to reuse the raw table dumps for downstream analysis.“

Comment 1.3:

The sentence "we imported an ORACLE SQL data warehouse that employs state-of-the-art database technologies" is not very clear. What do you mean by "imported a data warehouse"? Could you provide more details about the DBMS you used? To my understanding, you designed a relational model. You then implemented it in SQL using an Oracle DBMS (MySQL? or the native Oracle DBMS?) to store and query the data. Check page 9 description and add some details to avoid confusion. This is not a key passage though, I am sure that you handled the data somehow, and the paper's focus is not on this.

Response 1.3:

Thanks for this useful hint to consider more technical details of our database infrastructure. We revised the mentioned sentence in the manuscript to:

“To support on-line analytical data processing and convenient data access, a data warehouse was built based on the extracted data set. To scale appropriately, an ORACLE Enterprise RDBMS was applied. It enables analytical real-time SQL queries over millions of data points by means of in-memory materialized views, vectorization and columnar storage. This in turn was the basis to guarantee an appropriate user experience for the subsequently presented web application, which provides interactive, on-line calculation of metrics from NSD citations to various filters and data groupings, to drill-down chart and link data to the original records in ENA and ePMC.”

Comment 1.4:

"reference integrity between the tables was checked" -> This is a "weird" statement. Reference integrity is a constraint to guarantee the consistency of data. You "check the integrity" when you store the data in the DB, and if it is not validated, the data cannot be stored in the DB. So, I do not understand this sentence that is not explained anymore. Indeed, the paragraph continues by talking about the SQL queries to count the paper identifiers (this is not directly linked to reference integrity, or at least you should explain what you mean).

Recent analysis about issues related to ORCID ids and duplication of ids can be found here: http://ceur-ws.org/Vol-2816/paper10.pdf

Response 1.4:

We agree with the reviewer that reference integrity is a constraint to guarantee the consistency of data. In the manuscript we were unclear about the level of its use. In contrast to an OLTP centered database implementation of consistency checks by check constraints, such as foreign keys, we applied the reference integrity check in the ETL process to uncover potential reference integrity issues in the data set that results from the data extraction process. Indeed, we used a preliminary data warehouse instance without any formal SQL-DDL foreign key constraints, but SQL-based plausibility checks. E.g. by counting unique paper identifiers, ENA accessions, and country tags and checked number of joinable ENA and ePMC records. This lead to improvements of country references by the add country names synonyms to the country table or the use of a logical OR operator to join the ENA primary publication to ePMC by PMID, DOI or PMCID. A further example was the revision of the first approach to extract ENA accession number as reference to ENA from ePMC records. Because of obvious mismatch of expected counts to actual extracted ones for some exemplar papers, like genome assemblies of plants, we concluded that project accession numbers should also be considered as ENA citation too and revised and expanded the data mining task respectively.

In this context we thank the reviewer for this comment and improved the mentioned paragraph *"Retrieval of referenced IDs"*:

"Next, reference consistency among the extracted ENA and ePMC records were checked by ETL test runs. This resulted in a preliminary data warehouse instance, which allowed SQL-based plausibility checks. Such as the count unique paper identifiers, ENA accessions, and country tags or count the number of records in preliminary joining ENA and ePMC records over different combination of PMID, PMCID and DOI. We specifically checked whether ENA records refer to a valid country. For 217.40 ENA records out of 18.034.192 was it not the case, e.g. country tag "Western Sahara" (ENA accession HM034625) or empty country tag (ENA accession KM654101). Those could in some cases resolved by manual addition of synonyms to the country table that reflects the current valid UN agreed assignments. In case of empty country tags, we found some were annotated with geographical location. However, we left them empty to not intransparently change primary data.
Further we checked, if primary papers referenced in an ENA record exist in the ePMC databases. Here, a total of 6,753,891 ENA records refer either by DOI, PMID, or PMCID to 351,119 ePMC records, i.e. some use DOI only, some DOI and PMID etc."

In respect to the comment "Recent analysis about issues related to ORCID ids and duplication of ids can be found here: http://ceur-ws.org/Vol-2816/paper10.pdf" we added this reference to the manuscript as new citation [24]:

“Baglioni, M., Mannocci, A., Manghi, P., Atzori, C., Bardi, A., and Bruzzo, S. L. Reflections on the misuses of ORCID ids. In CEUR Workshop Proceedings of the 17th Italian Research Conference on Digital Libraries, http://CEUR-WS.org. pp. 117–125, 2021.”

Comment 1.5:

Table 1 is not that useful; it can be described in the text that you did the experiment and verified the discrepancies between open access publications and paywalled papers. It is a well-known problem, and it is not analyzed in-depth here. I think you can get rid of it without affecting the quality of the paper.

Response 1.5:

We agree that Table 1 reflects the issues formulated already in the text. A further intention was to show particular cases, where the suggested improvements were beneficial. Following the intention of the GigaScience Data Note policy, the intention here is to give additional interested parties, like software developers, publishers, or data curators some examples for further investigation. Thus, we would like to keep Table 1.

Comment 1.6:

Figure 4, like all the other images, is not readable. I directly accessed the Webapp, which works fine.

Response 1.6:

The quality was improved by increasing the resolution to 1200 dpi. We are sure that the rendering process at the editorial office may benefit and the resulting PDF will have a better readability.

Comment 1.7:

The paper is well-written, and the data collection is fine. Nevertheless, the article is a bit anti-climatic because there, not many provided insights. You discuss what we can do with the data, but little analysis of the data themselves. We could use some more in-depth analysis and a few insights about the achievable outcomes we can get using the collected data. Also, more about the best practices that should be defined in the field would be a nice addition.

Response 1.7:

This observation is particularly true and result of the authors' decision to separate the findings of the underlying study into a technical dataset paper and a policy-focused paper about the analysis, interpretation and conclusions. This paper was announced to the editorial board within the submission process and was submitted to GigaScience (submission ID GIGA-D-21-00228). In order to keep a reasonable timeline for publication, we would like to follow the editor's suggestion and publish a pre-print of a policy analysis paper (DOI: 10.1101/2021.08.02.454535). Furthermore, we will give more background in the WebApp in the details panel below every chart. This will provide users of the data more insight as well.

In respect to the suggested addition to best practice we are note sure about the reviewers intension. Regarding a technical aspect concerning the citation practice of NSD in scientific literature, conclusions can be drawn from the methods section that authors should reference the used sequences as explicitly as possible via a list of individual accession numbers or project accessions according to the citation policy of the respective journals. This aspect was indicated in the section "Data validation and quality control".

Another aspect would be the consistent labeling of the origin of biological material. As noted in section Methods, 15% of the ENA sequences are country tagged. This implies that appropriate regulation on the part of the data repositories could be debated. Although the discussion of this aspect is not in the scope of this data note, the publication of this manuscript could certainly support a discourse.

A best practice in terms of implications for the use and provision of DSI in the context of the CBD negotiations is a comprehensive discourse to which this data note contributes a factual basis. The resulting possible interpretations and implications will be explained in a companion paper as mentioned before.

Answers to comment of reviewer #2

The idea of curating this dataset is both important, and can contribute to the scientific community. Additionally, in most parts, the paper is well written.
However, the manuscript has some major issue that needs to solve before it would be ready for publication.

The Good:
- The dataset presented in the paper can be very useful to the scientific community
- The authors invested many efforts in making the paper reproducible. Both the project's code and dataset are open
- The project has a friendly and helpful web interface.

Things that need to improve:

Comment 2.1:

Although this paper is not a standard research paper, the article is missing more context to other works.
I believe the context of the manuscript will be more explicit by adding a Related Work section that provides an overview of other papers that generated similar datasets.

Response 2.1:

Please see our responses to similar comments by reviewer 1 in response 1.7.

Comment 2.2:

-Most of the analysis is based on the PubMed datasets, which is a relatively small dataset.
There are other open datasets that I think it is important to use to get a fuller picture, such as Microsoft Academic, AMiner, Semantic Scholar, bioXiv, and arXiv.
I understand that performing a full-text search on these datasets can be challenging. However, the paper's results need to be validated by using some of these datasets.

Response 2.2:

The goal of the study, in the first place, was not to extract all links, however. Rather, the data integration pipeline presented should achieve a good balance between recall and precision with respect to the data integration scenario at hand. The partner in the underlying research project is the EBI. The EBI databases were used with the

goal of evaluating possible approaches to quantify DSI usage. In addition, the use case for the analysis was to estimate DSI use in science for policy makers. We also recognize a focus on open access and the value that these open datasets provide. ePMC-API was therefore accepted as a reasonable trade-off between the computational burden of screening more than 18 million ENA accessions and a potential loss of sensitivity.
However, we agree with this comment and will continue to develop the identifier text extraction pipeline in a follow-up project that has already been approved. Some possible improvements have been highlighted in the Data Validation and Quality Control section of the Access Constraints section.
This rationale is now reflected by a text improvement in the "Access Restrictions" section on page 11.

"..., scanning PDF encoded manuscripts, the use of sophisticated text mining methods and integration of commercial text mining software could improve the recall and precision of NDS citation in texts as well. However, as the spirit of the project in which this analysis took place, with a heavy emphasis on open access and ENA API ..."

Comment 2.3:

The manuscript's quality needs to be improved (text, figures' resolutions, etc.).

Response 2.3:

As reported in response 1.1 to a similar feedback of reviewer 1, the figures' resolution was increased to support a suitable PDF rendering in the GigaScience manuscript processing pipeline.

Comment 2.4:

In my opinion, the overall structure of the paper can be improved.

Response 2.4:

Thanks for this comment. The structure of the paper followed the GigaScience authors instruction for data notes. In order to meet this hint, we included before the first subsection "Context" at page 3 an overview of the paper content:

"With this in the background, this Data Note subsequently presents the context of the dataset for quantified NSD use. As such, the method for extracting NSD citations from the scientific literature is described, as well as the technical details of constructing the data warehouse. The Data Validation and Quality Control section discusses the refinement

process of the data extraction pipeline and potential shortcomings arising from the available data quality, the provided APIs and the suggested potential improvements. Finally, the potential for reuse of the dataset through the WiLDSI web app is presented, as well as further potential for tracking genetic resource use in the scientific literature and aspects of quantifying DSI use in the context of benefit-sharing discussions under the CBD."

Comment 2.5:

There is no need to explain the FAIR data principle

Response 2.5:

In the context of the CBD negotiations, the role of NSD/DSI garnered immense interest and raised concern across the international scientific community. This was the background for this data note. The intention of the mentioned sentence with reference to FAIR was meant to be a transition to the global benefit of free and open use of DSI as demonstrated particularly in science. We refrained from introducing the FAIR criteria. Rather we intended to illustrate the emerging tension field of open science and FAIR as synonym and benefit sharing discussion.

Comment 2.6:

Using Microsoft Academic dataset can assist in mapping between author to a unique id

Response 2.6:

Thanks for the important suggestion. The ambiguous identification of authors and their affiliation is of importance. Thus, we included this aspect in the quality discussion of the manuscript. As follow-up to response 2.2 and extension to response 1.1 for reviewer 1, we agree that Microsoft Academic is a powerful service, that could assist in mapping between author to a unique ID, like ORCID. Nevertheless, we would have needed to purchase a commercial license to use the service in a programmatic way in the data extraction pipeline and to publish the results. As noted above, the aim of the manuscript was to apply the open public tools available from ePMC and ENA to ensure a broad reproducibility of the presented DSI quantification pipeline in context of the policy discussions.

Comment 2.7:

Mapping between an institute or location to a country can be more accurately done by utilizing geolocation code packages, such as geopy

Response 2.7:

Thanks to refeer by this comment to the importance of having consistently geolocated authors and NSD material orign. In fact 15% of all ENA sequences comprise a country tag as provenance in terms of international law and in context of the data notes objective. In respect to the potential envoronmental context of the "pick-up" location of bio material, 23% of them annotated by specific geo-coordinates, which could be used to validate the material provenance of each ENA deposited sequence data. But in respect to the background of this paper it seamed to be sufficient to considered the country tag only.
In case of scientific literature, the use of geolocation to validate or curate authors affiliation, if unique institutes identifiers, for example by ROR identifier, would be broadly available. An indirect geo location of authors affiliation by ROR use in authors ORCID record would be a further source for the near future. But currently, a minor fraction of all authors/paper pairs are annotated by an ORCID. Furthermore, how much of these ORCIDs are maintained consistently with authors affiliation history is unknown.

Answers to comment of reviewer #3

I have reviewed this manuscript with integrity, but I'm a little confused about it because I usually use NCBI PubMed/GenBank data. If my points are off the mark, please point them out.

Comment 3.1:

In NCBI PubMed, the nucleotide sequence entries referenced in the article are listed in PubMed data as external DB links (although not perfect), and by extracting these, the relationship between the PubMed and Nucleotide entries can be extracted. The NCBI website also provides these links from Nucleotide in the Related information section (e.g. https://pubmed.ncbi.nlm.nih.gov/19193256/). I found that the ePMC website also has a link in the Data section for nucleotide sequence entries referenced in the paper (e.g., https://europepmc.org/article/MED/19193256). Do you use any of these external links in ePMC data in this work? I think it is very difficult to extract nucleotide IDs by text mining, especially since Nucleotide sequence IDs are not in a fixed format. I think these links will be a great help in doing text mining.

Response 3.1:

Thanks for the comment. Indeed, we also use the one extended version of the pipeline used in ePMC to identify ENA accessions, rather than the pipeline from NCBI Genbank. However, the goal of the study was not so primary as to extract all links. Rather, the data integration pipeline presented was intended to achieve a good balance between recall and precision with respect to the data integration scenario at

hand. This is because the underlying literature as well as the sequence data are subject to dynamics and the citation quality of the NSD are very heterogeneous, as discussed in the "Data Validation" section. Therefore, the initial focus for this dataset was on a quantitative assessment of DSI use under manual quality review.
This resulted in this first integrated quality-checked dataset, which is intended to be published in conjunction with a web application for its exploratory evaluation. However, we agree with the reviewer, have the potential for further work already in the manuscript, and will take up these points in a follow-up project that has already been approved.

Comment 3.2:

2. In NCBI PubMed, MeSH keywords are assigned to each article for indexing the literature. MeSH keywords also include country keywords (e.g. https://pubmed.ncbi.nlm.nih.gov/19193256/). In ePMC Is it possible to use keywords like MeSH in ePMC? Do you have any opinions about using such country keywords?

Response 3.2:

Thanks for pointing this out. Indeed, some primary publications on NSD annotate the material or origin as a keyword in the corresponding publication in addition to the Country tag in ENA for the deposited sequences. In this particular case, Kenya is also consistently annotated to the 92 ENA sequences linked to this primary publication. However, a consistency analysis between keywords in primary publications, and the country tag in the original ENA was not the objective of this work.

Comment 3.3:

I found some great statistics and visualizations of this data on the site the authors provide about it. I would be happy to show these in this manuscript as a result of this work, but please follow the journal's policies and precedents.

Response 3.3:

Thanks for this. Nevertheless, we announced to the editorial board a companion paper that will more deeply elaborate and interpret the charts. This will be linked to this data note. Furthermore, the charts in the Web application have been updated and include more detail to the visualised quantification in respect to the DSI discussions under the Convention on Biological Diversity. In concrete the charts were consolidated and a scatter plot in panel 6 - “Network Graph“ was added that visualise details of the global relationship between providing and using of DSI. The charts in panel 2 - “country use of DSI” and panel 3 - “World Maps” are updated and

count all publications instead of only secondary publications. We updated the charts in both panels because it’s the same data but visualised in different types of graphs. Moreover, a new graph was added in panel 5 - “coalition collaboration” which displays the DSI use in all publications from the different economic groups. This is now the first graph on this page. We also moved chart 5.6 - “BRICS-G77-OECD consumption/contribution“ to the fourth position. In panel 4 - “hemisphere collaboration” graph 4.4 - “Distribution of publications using DSI with authors of n-different countries” was deleted.

Comment 3.4:

Do the authors think that users should reuse the created data for this product? Or is it recommended that users create their own data using the creation program? If the former, what is your plan for the frequency of updating the data?

Response 3.4:

We hope that readers will do both: some will explore the data using the web app and others will re-run the analysis on future or otherwise expanded datasets. Regarding data updates, please refer to response 3.1. The aforementioned follow-up project will work on a data update pipeline, with the goal of updating the data every six months.

Comment 3.5:

In Figure 1, I felt that it would be easier for the reader to understand if I emphasized (by changing the line or fill of the box) whether the data in each step is Nucleotide data, literature data, or ID pairs extracted from those data.

Response 3.5:

Indeed, the layout of Figure 1 aimed to illustrate the complex data extraction and integration workflow in a sketchy manner. The current highlighting and sectioning into a, b and c intended to reflect and logically group the processing steps. The suggestion to emphasize the data domain would surely improve illustrative quality. Thanks for this hint, which we followed and reshaped the Figure 1 towards a more visually clear tagging of nucleotide and literature data.

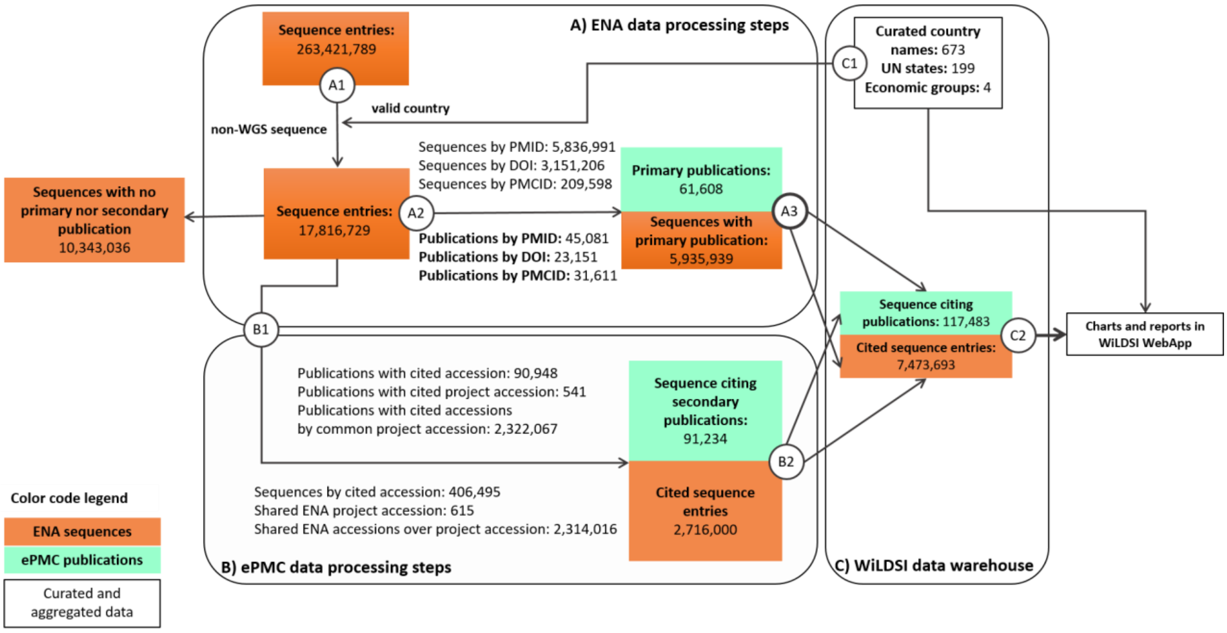

Sequence entries: 263,421,789
A) ENA data processing steps
A1
valid country
non-WGS sequence
C1
Curated country names: 673
UN states: 199
Economic groups: 4
Sequences by PMID: 5,836,991
Sequences by DOI: 3,151,206
Sequences by PMCID: 209,598
Primary publications: 61,608
Sequences with no primary nor secondary publication 10,343,036
Sequence entries: 17,816,729
A2
A3
Sequences with primary publication: 5,935,939
Publications by PMID: 45,081
Publications by DOI: 23,151
Publications by PMCID: 31,611
Sequence citing publications: 117,483
Cited sequence entries: 7,473,693
C2
Charts and reports in WiLDSI WebApp
B1
Publications with cited accession: 90,948
Publications with cited project accession: 541
Publications with cited accessions by common project accession: 2,322,067
Sequence citing secondary publications: 91,234
B2
Color code legend
ENA sequences
ePMC publications
Curated and aggregated data
Sequences by cited accession: 406,495
Shared ENA project accession: 615
Shared ENA accessions over project accession: 2,314,016
Cited sequence entries 2,716,000
B) ePMC data processing steps
C) WiLDSI data warehouse
