# Supplementary material for: Quantitative monitoring of nucleotide sequence data from genetic resources in context of their citation in the scientific literature
Source: Gigascience. 2021 Dec 29;10(12):giab084. doi: 10.1093/gigascience/giab084 (PMC8716361; doi:10.1093/gigascience/giab084)
Supplement: giab084_GIGA-D-21-00130_Revision_2 [file giab084_giga-d-21-00130_revision_2.pdf]

GigaScience

Quantitative monitoring of nucleotide sequence data from genetic resources in context of their citation in the scientific literature

--Manuscript Draft--

Manuscript Number:	GIGA-D-21-00130R2
Full Title:	Quantitative monitoring of nucleotide sequence data from genetic resources in context of their citation in the scientific literature
Article Type:	Data Note
Funding Information:	Bundesministerium für Bildung und Forschung (031B0862) — Dr Amber Hartman Scholz
Abstract:	Background<br><br>Linking nucleotide sequence data (NSD) to scientific publication citations can enhance understanding of NSDs provenance, scientific use, and reuse in the community. By connecting publications with NSD records, NSD geographical provenance information, and author geographical information, it becomes possible to assess the contribution of NSD to infer trends in scientific knowledge gain at the global level.<br><br>Findings<br><br>We extracted and linked records from the European Nucleotide Archive to citations in open-access publications aggregated at Europe PubMed Central for this data note. A total of 8,464,292 ENA accessions with geographical provenance information were associated with publications. We conducted a data quality review to uncover potential issues in publication citation information extraction and author affiliation tagging and developed and implemented best-practice recommendations for citation extraction. We constructed flat data tables and a data warehouse with an interactive web application to enable ad hoc exploration of NSD use and summary statistics.<br><br>Conclusions<br><br>The extraction and linking of NSD with associated publication citations enables transparency. The quality review contributes to enhanced text mining methods for identifier extraction and use. Furthermore, the global provision and use of NSD enable scientists worldwide to join literature and sequence databases in a multidimensional fashion. As a concrete use case, we visualized statistics of country clusters concerning NSD access in the context of discussions around digital sequence information under the United Nations Convention on Biological Diversity.
Corresponding Author:	Matthias Lange<br>Institute of Plant Genetics and Crop Plant Research: Leibniz-Institut fur Pflanzengenetik und Kulturpflanzenforschung Gatersleben<br>Seeland, Sachsen-Anhalt GERMANY
Corresponding Author Secondary Information:	
Corresponding Author's Institution:	Institute of Plant Genetics and Crop Plant Research: Leibniz-Institut fur Pflanzengenetik und Kulturpflanzenforschung Gatersleben
Corresponding Author's Secondary Institution:	
First Author:	Matthias Lange
First Author Secondary Information:	
Order of Authors:	Matthias Lange
	Blaise T.F. Alako
	Guy Cochrane

Powered by Editorial Manager® and ProduXion Manager® from Aries Systems Corporation

	Mehmood Ghaffar
	Martin Mascher
	Pia-Katharina Habekost
	Upneet Hillebrand
	Uwe Scholz
	Florian Zunder
	Jens Freitag
	Amber Hartman Scholz
**Order of Authors Secondary Information:**	

Response to Reviewers:

Dear editors, dear reviewers,

We are very appreciative of the review and further comments on our article. In the following letter, we answer all questions and provide comments on the intended revisions. All changes in the manuscript for this second revision are highlighted in green. The changes of first revision are highlighted still in yellow.
Please note that the co-author “Florian Zunder” provided his ORCID (0000-0001-6495-8611).
The point-by-point response to the reviewers' comments can be found hereafter.

Answers to comment of reviewer #1

Comment 1.1:
The authors comprehensively answered to this reviewer comments. The quality of the paper is improved and the modifications are in line with what was expected.
I have no further observations.

Response 1.1:
Thank you very much for the review.

Answers to comment of reviewer #2

Comment 2.1:
After reading the submitted "policy paper," the goal and contributions of this study and dataset became clear.
I believe this dataset and data visualization interface can be beneficial for the academic community.

Response 2.1:
Thanks for the reviewer’s support and comments to improve the manuscript.

Comment 2.2:
It is very challenging to understand Figure 1. I recommend adding additional figures that better explains how each part of the system work with more details.

Response 2.2:

Thanks for the hint to improve the Figure 1. Indeed, a visualization of a complex data flow process is challenging task. The image objective is to give reasonable abstraction to the core components of the data flow and the statistics of processed data. To visualize the data sets, data domains and processing step, we sub-divided logical connected sub-processes as boxes, color-coded the two data domains, numbered each ELT data processing step accordingly and described the step in the figure caption. Furthermore, we improved the layout of the data flow visualization.
To direct address the reviewer hint, we add an ELT process flow diagram as sub-figure that shows the sequence and dependencies within the ELT process.

Comment 2.3:
Even though the quality of the figures was improved, they are still of low quality, and it is hard to read the figures, especially Figure 4.

Powered by Editorial Manager® and ProduXion Manager® from Aries Systems Corporation

	Response 2.3:<br>The resolution of the Figures is 1200 dpi. We will crosscheck the rendering quality of the proofread within the journal production team carefully.<br><br>Comment 2.3:<br>The paper needs to be carefully proofread for punctuation mistakes.<br><br>Response 2.3:<br>Thanks for the hint. The manuscript undergoes an additional proof read to correct the punctuation and further grammatical and spelling mistakes. The changes where highlighted accordingly.<br><br>Answers to comment of reviewer #3<br><br>Comment 3.1:<br>I was convinced by the author's response to my previous comment.<br><br>Response 3.1:<br><br>Thank you very much for the review.
**Additional Information:**	
**Question**	**Response**
Are you submitting this manuscript to a special series or article collection?	No
**Experimental design and statistics**<br><br><br>Full details of the experimental design and statistical methods used should be given in the Methods section, as detailed in our Minimum Standards Reporting Checklist. Information essential to interpreting the data presented should be made available in the figure legends.<br><br><br>Have you included all the information requested in your manuscript?	Yes
**Resources**<br><br><br>A description of all resources used, including antibodies, cell lines, animals and software tools, with enough information to allow them to be uniquely identified, should be included in the Methods section. Authors are strongly encouraged to cite Research Resource Identifiers (RRIDs) for antibodies, model organisms and tools, where possible.	Yes

Have you included the information requested as detailed in our Minimum Standards Reporting Checklist?	
**Availability of data and materials**<br><br>All datasets and code on which the conclusions of the paper rely must be either included in your submission or deposited in publicly available repositories (where available and ethically appropriate), referencing such data using a unique identifier in the references and in the “Availability of Data and Materials” section of your manuscript.<br><br>Have you have met the above requirement as detailed in our Minimum Standards Reporting Checklist?	Yes

Powered by Editorial Manager® and ProduXion Manager® from Aries Systems Corporation

Manuscript
Click here to access/download;Manuscript;2021-09-22_Manuscript_revised

Quantitative monitoring of nucleotide sequence data from genetic resources in context of their citation in the scientific literature

Matthias Lange[1] [0000-0002-4316-078X], Blaise T.F. Alako[2] [0000-0001-6859-4421], Guy Cochrane[2] [0000-0001-7954-7057], Mehmood Ghaffar[1] [0000-0001-5062-5429], Martin Mascher[1,3] [0000-0001-6373-6013], Pia-Katharina Habekost[1,4] [0000-0002-3461-0881], Upneet Hillebrand[5] [0000-0002-3312-4013], Uwe Scholz[1] [0000-0001-6113-3518], Florian Zunder[1,4] [0000-0001-6495-8611], Jens Freitag[1] [0000-0001-6905-5497], Amber Hartman Scholz[5] [0000-0002-3461-0881]

[1]Leibniz Institute of Plant Genetics and Crop Plant Research, Germany

[2]European Molecular Biology Laboratory, European Bioinformatics Institute, Wellcome Genome Campus, Hinxton, United Kingdom

[3]German Centre for Integrative Biodiversity Research (iDiv) Halle-Jena-Leipzig, Leipzig, Germany

[4]Harz University of Applied Sciences, Germany

[5]Leibniz Institute DSMZ-German Collection of Microorganisms and Cell Cultures, Germany

Abstract

Background

Linking nucleotide sequence data (NSD) to scientific publication citations can enhance understanding of NSDs provenance, scientific use, and reuse in the community. By connecting publications with NSD records, NSD geographical provenance information, and author geographical information, it becomes possible to assess the contribution of NSD to infer trends in scientific knowledge gain at the global level.

Findings

We extracted and linked records from the European Nucleotide Archive to citations in open-access publications aggregated at Europe PubMed Central for this Data Note. A total of 8,464,292 ENA accessions with geographical provenance information were associated with publications. We conducted a data quality review to uncover potential issues in publication citation information extraction and author affiliation tagging and developed and implemented best-practice recommendations for citation extraction. We constructed flat data tables and a data warehouse with an interactive web application to enable *ad hoc* exploration of NSD use and summary statistics.

Conclusions

The extraction and linking of NSD with associated publication citations enables transparency. The quality review contributes to enhanced text mining methods for identifier extraction and use. Furthermore, the global provision and use of NSD enable scientists worldwide to join literature and sequence databases in a multidimensional fashion. As a concrete use case, we visualized statistics of country clusters concerning NSD access in the context of discussions around digital sequence information under the United Nations Convention on Biological Diversity.

Keywords

data citation, nucleotide sequence data, Europe PMC, European Nucleotide Archive, text mining, Convention on Biological Diversity, digital sequence information

Data Description

Nucleotide Sequence Data (NSD) plays a fundamental role in biological research ranging from public health and medical applications to understanding the molecular basis of life and evolution, such as how genes (mis)function in disease mechanisms [1], insights into ecosystem functioning and biodiversity conservation, and to assist in breeding new plant variety and animal breeds enabling food security and sustainability [2]. Scientifically, NSD plays a significant role in mechanistic modelling of species evolution [3], genotype-phenotype correlation [4] to identify and mitigate risks to species, track their illegal trade, identify the geographical origin of products, and plan conservation management strategies [5].

These applications demonstrate the overall value of NSD use and application and have triggered a political debate about benefit-sharing from genetic resources (G.R.). Under the Convention on Biological Diversity (CBD) and the Nagoya Protocol [6] as well as the International Treaty on Plant Genetic Resources for Food and Agriculture (ITPGRFA), the topic of "digital sequence information" (DSI) has garnered immense interest and raised concern across the international scientific community. Discussions have focused on using NSD from G.R.s, as DSI is an undefined and non-scientific term. Due to the exponential growth of public sequence and downstream databases [7], many parties are concerned that insufficient benefit-sharing occurs. Datasets such as this one provide an opportunity for evidence-based policymaking to analyze global trends in NSD provision and use as well as other science policy fields, including scientific strategic development and internationalization.

With this in the background, this Data Note subsequently presents the context of the dataset for quantified NSD use. As such, the method for extracting NSD citations from the scientific literature is described and the technical details of constructing the data warehouse. The Data Validation and Quality Control section discuss the refinement process of the data extraction

pipeline and potential shortcomings arising from the available data quality, the provided APIs and the suggested possible improvements. Finally, the potential for reuse of the dataset through the WiLDSI web app is presented, as well as the further potential for tracking genetic resource use in the scientific literature and aspects of quantifying DSI use in the context of benefit-sharing discussions under the CBD.

Context

FAIR (findable, accessible, interoperable, re-usable) data principles defined in 2016 in the FAIR Guiding Principles for scientific data management and stewardship [8] guide the design of open data sharing infrastructures as an enabling technology for economic growth and scientific progress. Data sharing principles were implemented at national and international levels. For example, the German Federal Ministry of Education and Research (BMBF) has funded an interdisciplinary project called "Science-based approaches for Digital Sequence Information" (WiLDSI) [9], which aims to (i) raise awareness and involve the international scientific community in the debate and decision-making process surrounding DSI, (ii) to identify and elaborate scenarios for open access to the NSD and (iii) to establish fair and sustainable benefit sharing.

In this context, transparent quantitative measures of NSD citation and reuse can inform decision-making processes surrounding the design of data sharing infrastructure, awarding scientific "credit" or political acknowledgement, or addressing the needs of commercial users [10]. In recent years, data citation has received increased attention from publishers, funding agencies, and infrastructure providers [11] [12]. However, best practices for NSD citation are still lacking, and those developed for scientific publications cannot be readily transferred.

Especially it is true for NSD, hosted by the core data infrastructure, the International Nucleotide Sequence Database Collaboration (INSDC) [13]. The European Nucleotide Archive (ENA) [14] and Europe PubMed Central (ePMC) [15] are, respectively, the European partners in INSDC and a repository of open access articles. Both have a long tradition of handling open data and document the heterogeneous quality of the author's data citation practices [16]. ePMC listed publications generally employ text-embedded ENA identifiers, like accession numbers, project accessions, or study accessions.

Methods

Figure 1 shows the extraction-load-transform (ELT) of ENA citations and the resulting data flow. First, ENA accessions and project accession numbers were extracted. Literature citations listed directly in the ENA entry were extracted parallel and called herd "primary publications". Next, we retrieved scientific papers that referred to these accession IDs via a full-text search using the ePMC REST API [17]. These publications we labelled "secondary publications". Finally, the extracted references and associated citation information were organized into six tables and imported into a data warehouse. As documented in Figure 2, its schema shows the referential dependencies, foreign keys, and primary key attributes. This is intended to support the advanced use of data exploration functionality in the web application. It is also intended to reduce users' barriers to reusing the raw table dumps for downstream analysis.

Figure 1. Schematic overview of the data flow of extract-load-transform (ELT) process to build the data warehouse from ENA and ePMC datasets. ENA records are parsed (A1),

filtered for valid country tags, and fed into ePMC RestFul API to extract matching secondary publication (B1) by ENA accession or project accession numbers. Primary publications are linked by ENA record (A2) to the DOI, PMCID or PMID. The resulting data sets are normalized as tables ENA_SEQUENCES, PMC_REFERENCES and loaded into the data warehouse (A3, B2). This is complemented by a manual ingested list of the world's countries and economics groups into the tables COUNTRIES and COUNTRY2GRP respectively (C1). Finally, SQL queries are applied to generate charts and reports in the Web application.

Five classes of citation patterns are used for ePMC publication as ENA identifiers where used: single accession number using word separation characters, e.g. hyphens, brackets, quotation marks; range notation of referenced accessions, text embedded enumeration, lists in supplement material or even embedded into figure bitmaps. The data extraction process from EMBL-ENA and ePMC was executed by Perl and Python scripts. The starting point of the data extraction process is the EMBL-ENA flat-file dump of release 143, which was obtained from the EMB-ENA FTP server and comprised 263,421,789 records. Next, all non-WGS ENA records were parsed to compile a relevant set of attributes for the table "ENA_SEQUENCES". A total of 17,820,136 ENA accessions with valid country tags (i.e., the /country field in the ENA entry, comprising 15% of all records) were included. Next, using the ePMC REST API, these ENA accessions were scanned in 36.7 million full-text articles accessible via PubMed. Due to performance reasons, this text tokenization was executed on-site at EBI in a compute cluster environment. From the resultant publications, those were select that have valid author country information and that either (b) cite an ENA sequence as a secondary publication[1] or

[1] designated as secondary in CBD context

(b) that are cited by ENA record as a primary publication[2]. The publications matching these criteria were compiled into the table "PMC_REFERENCES". In detail, 5,935,939 sequences cite 61,608 publications and 2,716,000 sequences are cited by 91,234 publications respectively. All scripts used in our analyses are available in GitHub [18].

<Figure 2>

Figure 2. Table schema of the WiLDSI data warehouse - The table ENA_SEQUENCES comprise metadata of a sequence stored in the EBI ENA database. The attributes accession and project accession are used to join secondary literature that cites sequences. The attribute country refers to the country table to resolve and group country tagged ENA sequences. The table PMC_REFERENCES consists of all ePMC published papers either referencing an ENA sequence by accession or project accession and references from ENA records as primary publication by either a DOI, PMID or PMCID.

The table "COUNTRY" was compiled and curated from U.N. state membership [19]. It comprises the three kinds of ISO-3166-1-codes, the official name (e.g. United Kingdom of Great Britain and Northern Ireland), a short version of the state name (e.g. the United Kingdom), commonly used names (e.g. Great Britain), and continent assignments. This table allows mapping from partly ambiguous country affiliation used in papers to the actual country designations recognized under international law. In particular, provinces or (partially) autonomous areas, such as Taiwan or West Sahara, are mapped to the legally responsible U.N. state party. Furthermore, several Ocean areas, for example, "Bismarck Sea" or "East China Sea", are grouped under the "Ocean" label along with more standard fields such as

[2] designated as primary in CBD context

"Atlantic Ocean"[3]. The assignment to economic groups is stored in table "COUNTRY2GRP". Here a 2-letter ISO code is assigned to rough economic groups OECD (Organization for Economic Cooperation and Development), BRICS (Brazil, Russia, India, China, South Africa), and G77 (representative of developing economies). To visualize countries in a world map, we used the table "GEOLOCATION" comprising the coordinates of the centroid of each country.

The tables are provided for download as CSV files (see section "Data Availability"). A data warehouse was built based on the extracted data set to support online analytical data processing and convenient data access. To scale appropriately, an ORACLE Enterprise RDBMS was applied. It enables analytical real-time SQL queries over millions of data points using in-memory materialized views, vectorization and columnar storage. This, in turn, was the basis to guarantee an appropriate user experience for the subsequently presented web application, which provides interactive, online calculation of metrics from NSD citations to various filters and data groupings, to drill-down chart and link data to the original records in ENA and ePMC.

Data validation and quality control

To assess the reliability of the extracted ePMC to ENA references, potential quality issues were evaluated by plausibility scans across data warehouse tables, including inspection of 20 randomly sampled papers performed by domain experts from IPK's sequence submission service team. We also considered review articles on data identifiers in life science literature

[3] "Ocean" is not equivalent to international waters under the United Nations Convention of the Law of the Seas (UNCLOS, where marine genetic resources and benefit-sharing are beeng discussed) but is in this context simply a consolidated term representing sampling in the marine environment.

[20] [21]. Finally, we applied the Dimensions text mining tool [22] to cross-check the sensitivity of ePMC API in respect of recall and sensitivity, e.g., to find false-negative hits such as published articles that reference ENA sequences but which were not matched by the ePMC REST API.

Country names

The country name had to match records in the country table. Here we found some obsolete or ambiguous country names, like Montenegro or West Sahara and historic country names, like the Soviet Union, which cannot be assigned uniquely to current U.N. states. Ambiguous country names were resolved manually and reverted to synonyms in the country table (e.g. Cote d'Ivoire to Ivory Coast, amongst others). ENA or PMC records with obsolete country tags were kept in the data set but ignored for summary statistics queries and excluded in the below quality check.

Retrieval of referenced IDs

Next, reference consistency among the extracted ENA and ePMC records was checked by extract-load-transform (ELT) test runs. This resulted in a preliminary data warehouse instance, which allowed SQL-based plausibility checks. Such as the count of unique paper identifiers, ENA accessions, and country tags or count the number of records in preliminary joining ENA and ePMC records over a different combination of PMID, PMCID and DOI. We specifically checked whether ENA records refer to a valid country. For 217.40 ENA records out of 18.034.192, was it not the case, e.g. country tag "Western Sahara" (ENA accession HM034625) or empty country tag (ENA accession KM654101). Those could, in some cases resolved by manual addition of synonyms to the country table that reflects the current valid U.N. agreed assignments. In the case of empty country tags, we found some were annotated

with geographical location. However, we left them empty to avoid a non-transparent change of primary data.

Further, we checked if primary papers referenced in an ENA record exist in the ePMC databases. Here, a total of 6,753,891 ENA records refers either by DOI, PMID, or PMCID to 351,119 ePMC records, i.e. some use DOI only, some DOI and PMID etc. Conversely, there are 9,589,900 ENA records without any primary literature reference. Furthermore, we confirmed that ePMC records, which cite secondary ENA accession or project numbers, can be resolved to records in ENA. We found 189,581 ePMC records that reference 2,801,072 ENA records by either accession or project accession number. A potential issue in using an identifier to cite ENA records is that authors sometimes use ENA study identifiers or even BioSample IDs. However, our pipeline considers ENA accession and project accession only.

Author identification

The combination of first and last names are not unique identifiers for human beings. ORCIDs provide unique identifiers for authors and are on their way to becoming compulsory for publications. Existing articles, however, are only occasionally associated with ORCID. Another potential issue is that it is possible to register multiple ORCID for one person. Identifying authors as a concatenation of author names and affiliations is error-prone [23, 24]. Therefore, author information was retained in the tables but not used for statistical analysis.

Range notation

Scientific publications may use ambiguous range notation to cite ENA accessions. As illustrated in Figure 3, hyphen as range notation aggregate a sequence of ENA accessions. Here, the authors assume an ordered sequence of accession numbers, and it is interpreted as such by human readers but is not recognized by programmatic text mining. Thus, in the data extraction used here, a potentially high number of ENA accessions are missed, and the dataset

underestimates the number of referenced DSI. This analysis is intended to support future work to address these shortcomings.

<Figure 3>

Figure 3: Example of range notation for ENA accession references. Within the selected part of publication with DOI: 10.3201/eid1502.080851, the actual number of cited ENA accessions is 35, but ePMC API matched eight only.

Access restrictions

Only open access publications were available for high-throughput text mining. To efficiently process 18 million ENA accession numbers, the ePMC REST API at EBI's local compute infrastructure was used. This causes a potential loss of recall compared to broad and integrative use of further state-of-the-art literature mining services that include articles behind paywalls. To get an estimation of potentially missed DSI citations, we used alternative tools that cover patent and closed access publications. We applied the commercial "Dimensions" [22] and the free "Lens.org" [25] search tools, which include patent and restricted-access publication, to compare recall performance for 20 randomly selected ENA accessions. This evaluation was performed within four weeks of the ePMC based text mining run to work with a comparable corpus. The results are compiled in Table 1. Specific hits to one of three approaches, ePMC, Lens, dimensions were observed. This is likely due to the larger corpora of Dimensions and Lens. For example, ENA accession AB076935 was linked to three public and three closed access publications, whereas ePMC did not report any matching publication. Differences in file format may explain some of the differences. There are cases where the PDF rendered articles differ from ePMC rendered HTML versions so that the PDF versions can contain more ENA accession numbers than HTML versions in ePMC. We did not

aim for an in-depth analysis of literature search tools. Still, our cursory overview supports the notion that a substantial number of publications relevant to NSD may be behind paywalls, scanning PDF encoded manuscripts, using sophisticated text mining methods, and integrating commercial text mining software could improve the recall and precision of NDS citation in texts as well. However, as the spirit of the project in which this analysis took place, with a heavy emphasis on open access and ENA API, we continued our analyses with the available dataset.

ENA ACCESSION	hits in ePMC	hits in Dimensions	hits in Lens	overlap Dimensions and ePMC	overlap Lens and ePMC
AB076935	0	6	0	0	0
AB076941	0	1	0	0	0
EU257628	3	5	0	2	0
AB326609	0	1	0	0	0
AM262332	0	2	0	0	0
EU575854	1	1	0	1	0
CP039348	0	1	0	0	0
DQ410599	1	1	0	1	0
EU293114	12	19	1	6	1
AY924392	10	7	2	6	2
EF607913	0	1	0	0	0
AY768827	0	1	0	0	0

Table 1. Comparison of ENA accession number query performance of APIs of EBI ePMC, Dimensions[4] and Lens[5].

Reuse potential

To further explore the data set, a web application was developed and is publicly accessible at https://wildsi.ipk-gatersleben.de. We focused mainly on understanding NSD/DSI usage in the context of fair and equal benefit sharing. The web interface illustrated in Figure 4 enables the interactive exploration of DSI use in science by a features text search, data aggregation across the data warehouse and cross-linking to the original ENA records and ePMC records. It enables further complex filtering, grouping as well as visualization as charts, world map projects, and network diagrams. Based on the use cases provided in this CBD context, fundamental questions regarding DSI usage are visualized in different relationships to answer questions such as: Which countries use DSI? Which countries (groups) contributed DSI? Are there countries that use DSI but do not contribute DSI? Four classes of use cases implement this: *general overview of DSI, per country use of DSI, collaborative use in economic and hemisphere groups, world map projection, DSI citation network*.

<Figure 4>

Figure 4. Screenshots of the WiLDSI Web Application. It consists of pages for (A) detailed data reports with integrated (B) drill down to sources, (C) charts of DSI usage scenarios, (D) per country DSI use and contribution etc.

---

4 Our queries used this URL pattern: https://app.dimensions.ai/discover/publication?search_text=AY924392&search_type=kws&search_field=full_search

5 Our queries used this URL pattern: https://www.lens.org/lens/scholar/search/results?q=AY924392&preview=true

Another reuse scenario is to document the flow of DSI associated with genebank resources across the scientific value chain from seed storage to genetic analysis. A prominent example is the role of molecular passport data as an instance of DSI to characterize plant genetic resources (PGR). Gene bank genomics is an emerging research field aiming at using high-throughput sequencing to characterize the genetic diversity in entire gene bank collections [26]. Recently, marker profiles from reduced representation sequencing data were reported for more than 20,000 accessions of the German Genebank [27]. Whole-genome shotgun sequencing has been used to characterize the genome of 3,000 rice accessions at the International Rice Research Institute [28]. The approach provides a so-called molecular passport that enables tracking the identity of accessions, identifying redundancies and cross-link international gene banks [29]. For these reasons, molecular passport data is poised to become an essential component of working with PGR in research and breeding contexts. Documenting the use of DSI associated with PGR would help genebank managers and administrators of genebank information systems monitor the use of their accessions in international research efforts and help justify the tremendous effort put into the maintenance and characterization of PGR in global genebanks. Documenting DSI could also help national authorities to enforce access and benefit-sharing schemes of the Nagoya protocol. The present enquiry into the status of DSI in public sequence archives has shown that sequence information of PGR is abundant, but tracing it back to the gene bank holdings it derives from can be challenging. In the coming years, gene bank managers, genome researchers and bioinformaticians should develop and enshrine standards and protocols for linking DSI in archives such as EMBL-ENA to gene bank information systems and meta-

databases as EURISCO [30]. Work in this direction is underway in the EU-funded project AGENT [31].

DSI and their free accessibility are essential for all life sciences, including biodiversity research, food security, human health, biological conservation and many other disciplines or research areas. Some countries contributing DSI fear that direct access to the increasing amount of freely available sequence information may undermine benefit-sharing schemes for genetic resources. The use of this data set supports evidence-based decision making in the context of international policy processes and global scale investigations into scientific use and reuse of NSD datasets and sub-disciplines thereof. Indeed, this article is intended as a companion paper for a timely publication on the policy implications of NSD (re-)use for DSI access and benefit-sharing discussions under the CBD published alongside it at *GigaScience* [32].

For future studies, the examples above could be complemented by more detailed use cases, including finer-grained groupings for data aggregation such as separation of genera, species and time ranges of publications. In combination with additional text classification techniques [33], it may be possible to cluster by research topics, e.g. considering only citations in the paper involving, say, COVID-19 or plant pathogen resistance.

Availability of source code and requirements

Project name: WiLDSI

Project home page: https://wildsi.ipk-gatersleben.de

Operating system(s): LINUX

Programming language: Oracle Application Express, Perl, Python3

Other requirements: HTML5 compatible web browser

License: GNU General Public License v3.0

All scripts used for data extraction are available from GitHub https://github.com/alakob/sequence-literature.

Data Availability

The charts, maps, and data tables are available in an interactive web application at http://wildsi.ipk-gatersleben.de. The data tables are published as CSV files in the e!DAL-PGP repository [34] under the DOI 10.5447/ipk/2021/8 [35]. The SQL queries implementing the use cases are linked and documented alongside each chart within the web application. An archival copy of the GitHub repository is available in the GigaScience GigaDB repository [36].

List of abbreviations

CBD: Convention on Biological Diversity; ITPGRFA, International Treaty for Plant Genetic Resources for Food and Agriculture DOI: Document Object Identifier; ELT: Extract, Load, Transform; EMBL: European Molecular Biology Laboratory; ENA: European Nucleotide Archive; ePMC: Europe PubMed Central; DSI: Digital Sequence Information - a synonym for nucleotide sequence data in international policy circles; GR: Genetic Resources; INSDC: Nucleotide Sequence Database Collaboration; NSD: Nucleotide Sequence Data - synonym to DSI in a technical and database context; ORCID: Open Researcher and Contributor ID; PGR: Plant Genetic Resources; WiLDSI: German: "wissenschaftsbasierte Lösungsansätze für digitale Sequenzinformation", English translation: Science-based Approaches for Digital Sequence Information

Ethics approval and consent to participate

Not applicable.

Consent for publication

Not applicable.

Competing interests

The author(s) declare that they have no competing interests.

Funding

This work was supported by the German Federal Ministry of Education and Research (BMBF) in the frame of the project “WiLDSI: Wissensbasierte Lösungsansätze für Digitale Sequenzinformation” (FKZ 031B0862) and IPK Gatersleben core funding.

Authors' contributions

Conceptualization: A.S., J.F., G.C., M.L.

Software: M.G., B.A., M.L., P.H., F. Z.

Data curation: U.H., J.F., M.L.

Investigation: A.S., J.F., U.H.

Supervision: M.L., A.S., J.F., G.C.

Writing original draft: M.G., M.L., M.M., A.S.

Writing review and editing: All authors

Funding acquisition: A.S., U.S.

Acknowledgements

We thank H. Miehe, M. Oppermann and T. Münch for technical support and hosting the web application. We also thank the thousands of authors that generated the data and publications

analyzed here, especially to those committed to open access which enables this global overview.

References

[1] E. R. Mardis, 'A decade's perspective on DNA sequencing technology', *Nature*, vol. 470, no. 7333, pp. 198–203, Feb. 2011, doi: 10.1038/nature09796.

[2] 'Digital sequence information | Commission on Genetic Resources for Food and Agriculture | Food and Agriculture Organization of the United Nations'. http://www.fao.org/cgrfa/topics/digital-sequence-information/en.

[3] P. A. Romero and F. H. Arnold, 'Exploring protein fitness landscapes by directed evolution', *Nat. Rev. Mol. Cell Biol.*, vol. 10, no. 12, pp. 866–876, Dec. 2009, doi: 10.1038/nrm2805.

[4] M. D. Ritchie, E. R. Holzinger, R. Li, S. A. Pendergrass, and D. Kim, 'Methods of integrating data to uncover genotype–phenotype interactions', *Nat. Rev. Genet.*, vol. 16, no. 2, pp. 85–97, Feb. 2015, doi: 10.1038/nrg3868.

[5] F. W. Allendorf, P. A. Hohenlohe, and G. Luikart, 'Genomics and the future of conservation genetics', *Nat. Rev. Genet.*, vol. 11, no. 10, pp. 697–709, Oct. 2010, doi: 10.1038/nrg2844.

[6] 'About the Nagoya Protocol'. https://www.cbd.int/abs/about/.

[7] A. Szalay and J. Gray, 'Science in an exponential world', *Nature*, vol. 440, no. 7083, pp. 413–414, Mar. 2006, doi: 10.1038/440413a.

[8] M. D. Wilkinson *et al.*, 'The FAIR Guiding Principles for scientific data management and stewardship', *Sci. Data*, vol. 3, no. 1, p. 160018, Dec. 2016, doi: 10.1038/sdata.2016.18.

[9] 'German Collection of Microorganisms and Cell Cultures GmbH: Digital Sequence Information'. https://www.dsmz.de/collection/nagoya-protocol/digital-sequence-information (accessed Mar. 31, 2021).

[10] N. Robinson-García, E. Jiménez-Contreras, and D. Torres-Salinas, 'Analyzing data citation practices using the data citation index', *J. Assoc. Inf. Sci. Technol.*, vol. 67, no. 12, pp. 2964–2975, Dec. 2016, doi: 10.1002/asi.23529.

[11] G. Silvello, 'Theory and practice of data citation', *J. Assoc. Inf. Sci. Technol.*, vol. 69, no. 1, pp. 6–20, Jan. 2018, doi: 10.1002/asi.23917.

[12] H. Cousijn *et al.*, 'A data citation roadmap for scientific publishers', *Sci. Data*, vol. 5, no. 1, p. 180259, Dec. 2018, doi: 10.1038/sdata.2018.259.

[13] M. Arita, I. Karsch-Mizrachi, and G. Cochrane, 'The international nucleotide sequence database collaboration', *Nucleic Acids Res.*, vol. 49, no. D1, pp. D121–D124, Jan. 2021, doi: 10.1093/nar/gkaa967.

[14] P. W. Harrison *et al.*, 'The European Nucleotide Archive in 2020', *Nucleic Acids Res.*, vol. 49, no. D1, pp. D82–D85, Jan. 2021, doi: 10.1093/nar/gkaa1028.

[15] M. Levchenko *et al.*, 'Europe PMC in 2017', *Nucleic Acids Res.*, vol. 46, no. D1, pp. D1254–D1260, Jan. 2018, doi: 10.1093/nar/gkx1005.

[16] Ş. Kafkas, J.-H. Kim, and J. R. McEntyre, 'Database Citation in Full Text Biomedical Articles', *PLoS ONE*, vol. 8, no. 5, p. e63184, May 2013, doi: 10.1371/journal.pone.0063184.

[17] 'Europe PMC - Developer resources', *Europe PMC*, Feb. 08, 2021. https://europepmc.org/developers.

[18] Alko, Blaise, *Exploratory Analysis and Predictive Modelling of sequences citation in the literature.* https://github.com/alakob/sequence-literature.

[19] *United Nations Member States*. https://www.un.org/en/about-us/member-states (accessed Mar. 31, 2021).

[20] Ş. Kafkas, J.-H. Kim, X. Pi, and J. R. McEntyre, 'Database citation in supplementary data linked to Europe PubMed Central full text biomedical articles', *J. Biomed. Semant.*, vol. 6, no. 1, p. 1, 2015, doi: 10.1186/2041-1480-6-1.

[21] J. A. McMurry *et al.*, 'Identifiers for the 21st century: How to design, provision, and reuse persistent identifiers to maximize utility and impact of life science data', *PLOS Biol.*, vol. 15, no. 6, p. e2001414, Jun. 2017, doi: 10.1371/journal.pbio.2001414.

[22] D. W. Hook, S. J. Porter, and C. Herzog, 'Dimensions: Building Context for Search and Evaluation', *Front. Res. Metr. Anal.*, vol. 3, p. 23, Aug. 2018, doi: 10.3389/frma.2018.00023.

[23] P. Sebo, S. de Lucia, and N. Vernaz, 'Accuracy of PubMed-based author lists of publications and use of author identifiers to address author name ambiguity: a cross-sectional study', *Scientometrics*, Mar. 2021, doi: 10.1007/s11192-020-03845-3.

[24] Baglioni, M., Mannocci, A., Manghi, P., Atzori, C., Bardi, A., and Bruzzo, S. L. Reflections on the misuses of ORCID ids. In CEUR Workshop Proceedings of the 17th Italian Research Conference on Digital Libraries, http://CEUR-WS.org. pp. 117–125, 2021.

[25] 'Results The Lens - Free & Open Patent and Scholarly Search', *The Lens - Free & Open Patent and Scholarly Search*. https://www.lens.org/lens (accessed Apr. 01, 2021).

[26] M. Mascher, M. Schreiber, U. Scholz, A. Graner, J. C. Reif, and N. Stein, 'Genebank genomics bridges the gap between the conservation of crop diversity and plant breeding', *Nat. Genet.*, vol. 51, no. 7, pp. 1076–1081, Jul. 2019, doi: 10.1038/s41588-019-0443-6.

[27] S. G. Milner *et al.*, 'Genebank genomics highlights the diversity of a global barley collection', *Nat. Genet.*, Nov. 2018, doi: 10.1038/s41588-018-0266-x.

[28] W. Wang *et al.*, 'Genomic variation in 3,010 diverse accessions of Asian cultivated rice', *Nature*, vol. 557, no. 7703, pp. 43–49, May 2018, doi: 10.1038/s41586-018-0063-9.

[29] X. Yu *et al.*, 'Genomic prediction contributing to a promising global strategy to turbocharge gene banks', *Nat. Plants*, vol. 2, no. 10, p. 16150, Oct. 2016, doi: 10.1038/nplants.2016.150.

[30] S. Weise, M. Oppermann, L. Maggioni, T. van Hintum, and H. Knüpffer, 'EURISCO: The European search catalogue for plant genetic resources', *Nucleic Acids Res.*, vol. 45, no. D1, pp. D1003–D1008, Jan. 2017, doi: 10.1093/nar/gkw755.

[31] 'Activated GEnebank NeTwork (AGENT)', *Global Access to Plant Genetic Resources*. https://www.agent-project.eu (accessed Feb. 23, 2021).

[32] A. Hartman Scholz, M. Lange, P. Habekost, P. Oldham, I. Cancio; G. Cochrane; J. Freitag, 'Myth-busting the provider-user relationship for digital sequence information', GigaScience, 2021, DOI:10.1093/gigascience/giaxxx

[33] J. Chen, U. Scholz, R. Zhou, and M. Lange, 'LAILAPS-QSM: A RESTful API and JAVA library for semantic query suggestions', *PLOS Comput. Biol.*, vol. 14, no. 3, p. e1006058, Mar. 2018, doi: 10.1371/journal.pcbi.1006058.

[34] D. Arend, A. Junker, U. Scholz, D. Schüler, J. Wylie, and M. Lange, 'PGP repository: a plant phenomics and genomics data publication infrastructure', *Database*, vol. 2016, p. baw033, 2016, doi: 10.1093/database/baw033.

[35] G. Cochrane et al. (2021-04-12): Quantitative monitoring of nucleotide information from genetic resources in context of their citation in the scientific literature. doi:10.5447/ipk/2021/8

[36] Lange M; Alako BTF; Cochrane G; Ghaffar M; Mascher M; Habekost P; Hillebrand U; Scholz U; Zunder F; Freitag J; Scholz AH (2021): Supporting data for "Quantitative monitoring of

nucleotide sequence data from genetic resources in context of their citation in the scientific literature" GigaScience Database. http://dx.doi.org/10.5524/100947

Figure 1 (2nd revision)
Click here to access/download;Figure;Figure_1.pdf

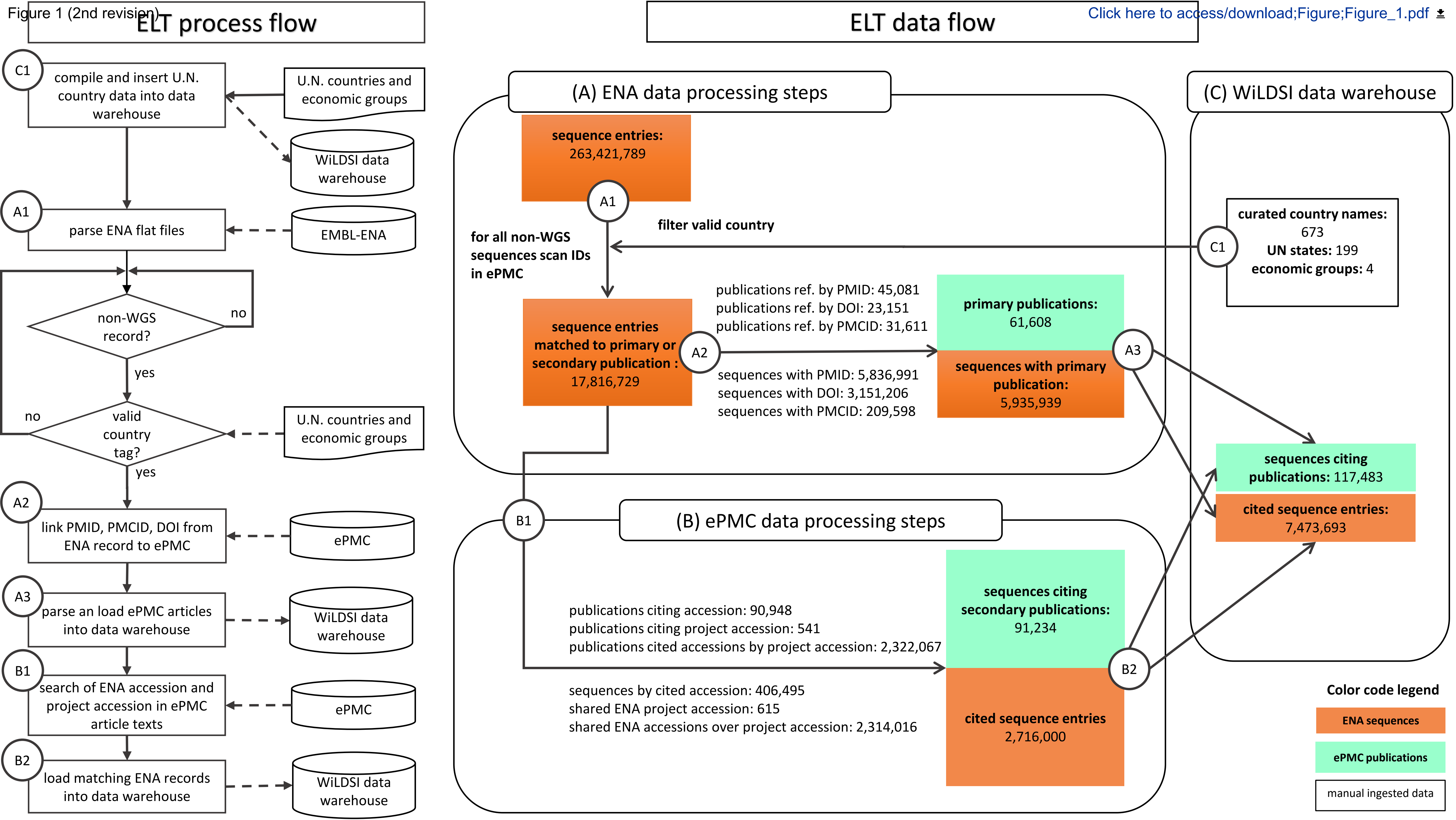

Figure 2 (revised)

Click here to access/download;Figure;Figure_2.pdf

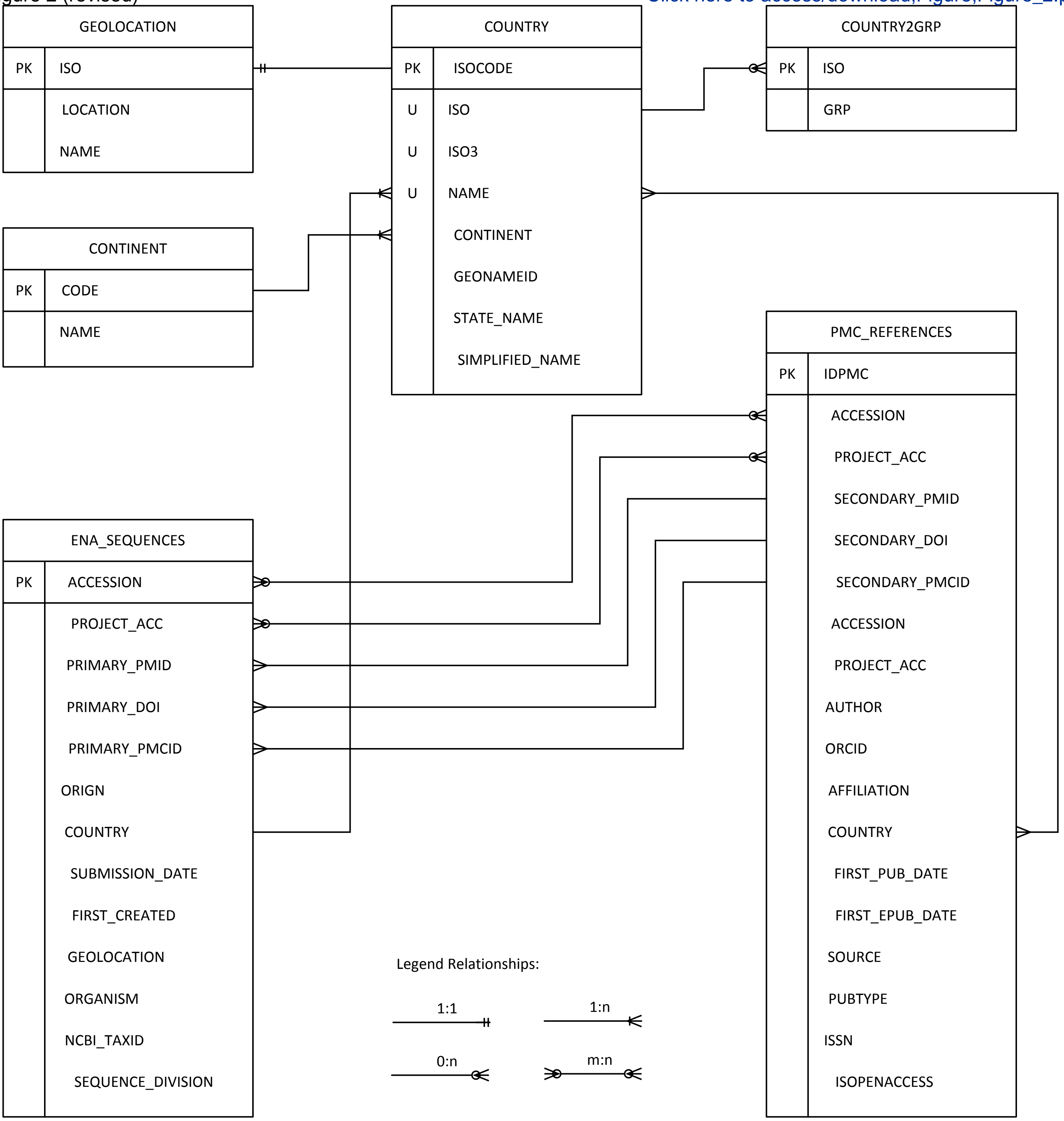

Figure 3 (revised) Click here to access/download;Figure;Figure_3.png

Table 4. Pairwise comparison of full-length nucleotide and amino acid sequences of the medium segment of Kupe virus with other nairoviruses*

Virus	Kupe	Dugbe ArD44313	HAZV	CCHFV
Kupe		61.9	54.7	52.1
Dugbe ArD44313	57.0		53.7	52.5
HAZV	47.7	44.4		50.8
CCHFV	43.0	38.3	41.4	

*Nucleotide identity (%) is shown above the diagonal, and amino acid identity (%) is shown below the diagonal. HAZV, Hazara virus; CCHFV, Crimean-Congo hemorrhagic fever virus.

EU257626, EU816906–EU816909). Results of phylogenetic analysis of the full-length S segment amino acid se-

L fragment alignment overlapping the RDRP core domain (Kupe virus nt 7292–7894) included sequences from 26 DUGV isolates obtained in Kenya in 1999 (GenBank accession nos. EU359010–EU359035), DUGV ArD 44313, and Kupe virus. Results of these comparisons are shown in Table 6. Sequence identities among 5 Kupe virus isolates for this fragment were nt 91.2%–100.0% and aa 98.5%–100.0% (GenBank accession nos. EU257628, EU816898–EU816901).

Discussion

Although little genetic information is available for most viruses in the genus *Nairovirus*, current classification

Figure 4 (revised)

Click here to access/download;Figure;Figure_4.png

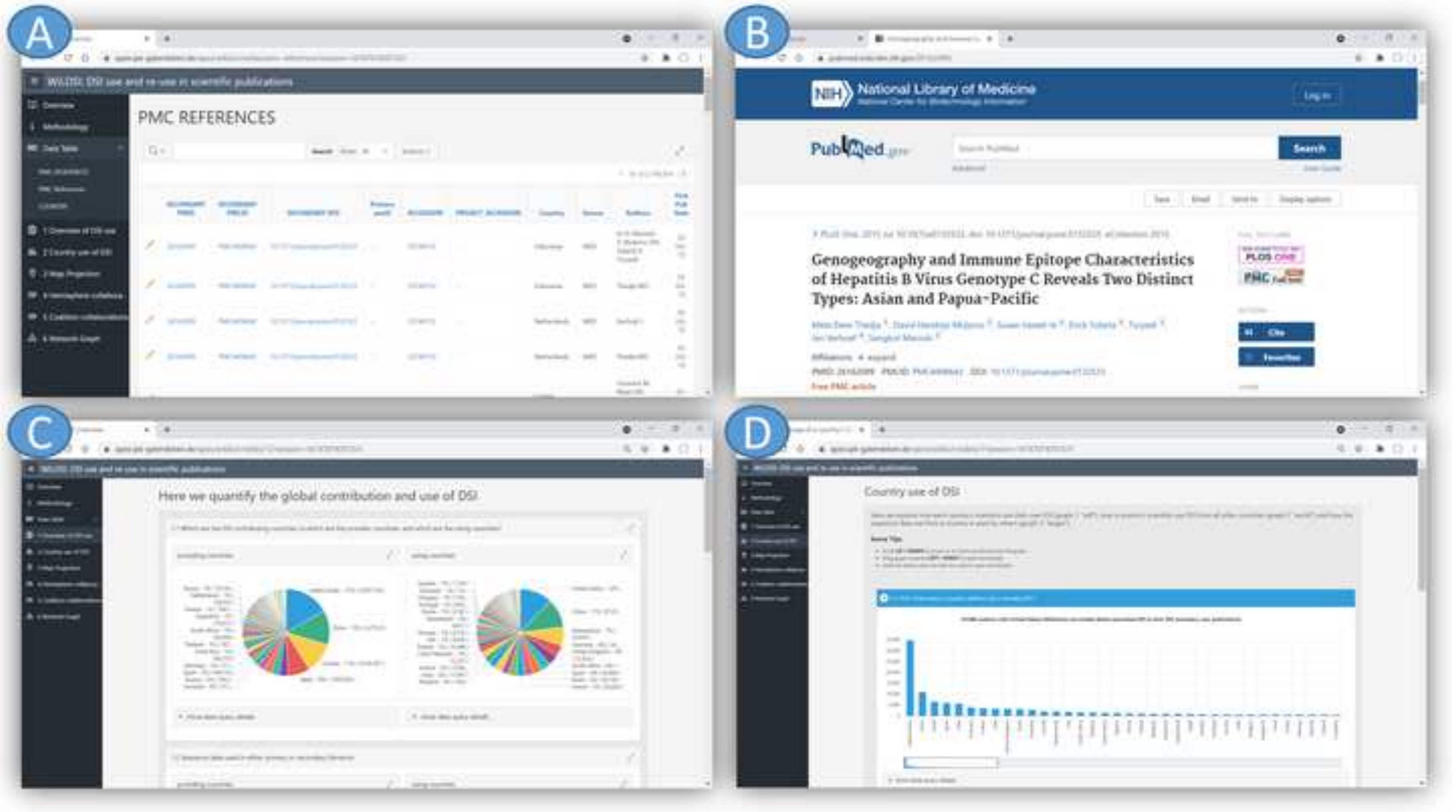

Click here to access/download;Personal Cover;Cover_letter_and_response_to_reviewer.docx

Dear editors, dear reviewers,

We are very appreciative of the review and further comments on our article. In the following letter, we answer all questions and provide comments on the intended revisions. All changes in the manuscript for this second revision are highlighted in green. The changes of first revision are highlighted still in yellow.
Please note that the co-author “Florian Zunder” provided his ORCID (0000-0001-6495-8611).
The point-by-point response to the reviewers' comments can be found hereafter.

Answers to comment of reviewer #1

Comment 1.1:

The authors comprehensively answered to this reviewer comments. The quality of the paper is improved and the modifications are in line with what was expected.

I have no further observations.

Response 1.1:

Thank you very much for the review.

Answers to comment of reviewer #2

Comment 2.1:

After reading the submitted "policy paper," the goal and contributions of this study and dataset became clear.
I believe this dataset and data visualization interface can be beneficial for the academic community.

Response 2.1:

Thanks for the reviewer’s support and comments to improve the manuscript.

Comment 2.2:

It is very challenging to understand Figure 1. I recommend adding additional figures that better explains how each part of the system work with more details.

Response 2.2:

Thanks for the hint to improve the Figure 1. Indeed, a visualization of a complex data flow process is challenging task. The image objective is to give reasonable abstraction to the core components of the data flow and the statistics of processed data. To visualize the data sets, data domains and processing step, we sub-divided logical connected sub-processes as boxes, color-coded the two data domains, numbered each ELT data processing step accordingly and described the step in the figure caption. Furthermore, we improved the layout of the data flow visualization. To direct address the reviewer hint, we add an ELT process flow diagram as sub-figure that shows the sequence and dependencies within the ELT process.

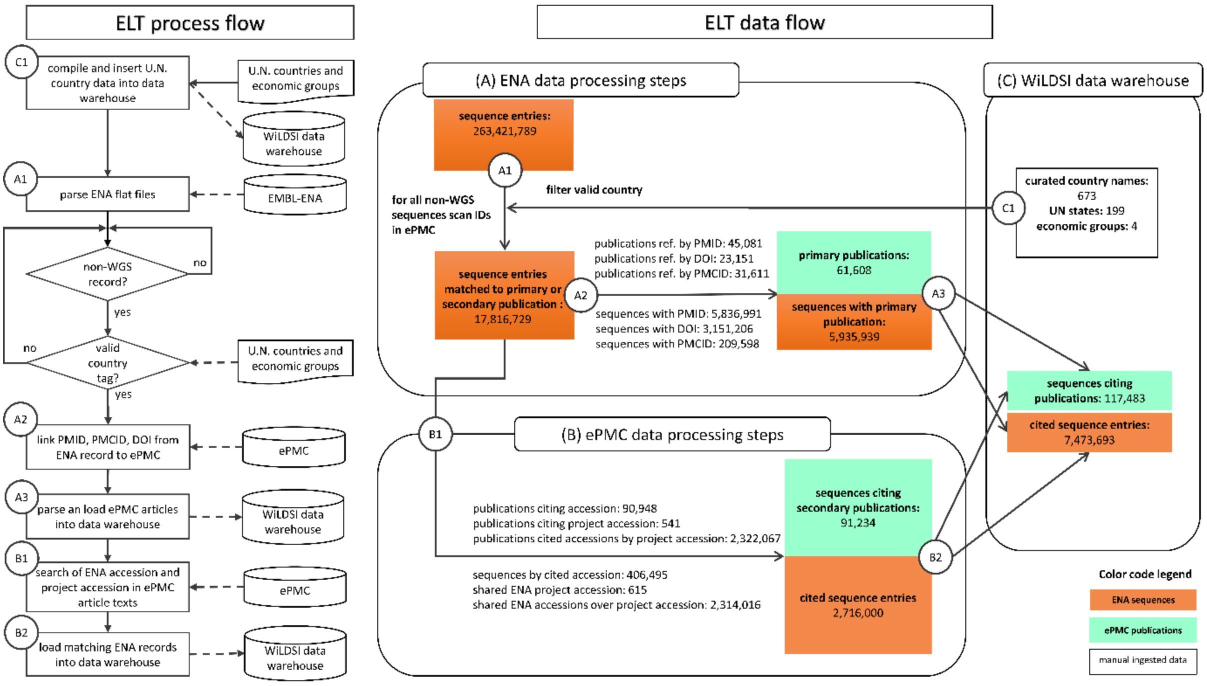

Comment 2.3:

Even though the quality of the figures was improved, they are still of low quality, and it is hard to read the figures, especially Figure 4.

Response 2.3:

The resolution of the Figures is 1200 dpi. We will crosscheck the rendering quality of the proofread within the journal production team carefully.

Comment 2.3:

The paper needs to be carefully proofread for punctuation mistakes.

Response 2.3:

Thanks for the hint. The manuscript undergoes an additional proof read to correct the punctuation and further grammatical and spelling mistakes. The changes where highlighted accordingly.

Answers to comment of reviewer #3

Comment 3.1:

I was convinced by the author's response to my previous comment.

Response 3.1:

Thank you very much for the review.
