# Supplementary material for: Quantitative monitoring of nucleotide sequence data from genetic resources in context of their citation in the scientific literature
Source: Gigascience. 2021 Dec 29;10(12):giab084. doi: 10.1093/gigascience/giab084 (PMC8716361; doi:10.1093/gigascience/giab084)
Supplement: giab084_Response_to_Reviewer_Comments_Original_Submission [file giab084_response_to_reviewer_comments_original_submission.pdf]

Author's Response To Reviewer Comments

Close

Dear editors, dear reviewers,

We are very appreciative of the review and comments on our article. In the following letter we answer all questions and provide comments on the intended revisions. All revisions in the manuscript are highlighted in red. To address the editor's suggestion to provide more insight into the implications and analysis of the data sets presented, we have submitted a tandem “policy paper” (your submission identifier GIGA-D-21-00228), published a pre-print in BioRxiv (DOI: 10.1101/2021.08.02.454535) and have added further explanatory text on each of the diagrams in the web application.

The point-by-point response to the reviewers' comments can be found hereafter.

Answers to comment of reviewer #1

Comment 1.1:
Figure 1 is not readable. The sampling process lowered the quality of the image and made the text not readable. Please, use vectorial images (e.g., PDF or EPS). Anyhow, I could understand the process from the descriptive text.

Response 1.1:
Sorry for the low quality of the rendered PDF. The figures’ resolution was increased to 1200 dpi and the transparent background was replaced by white to avoid potential PDF rendering issues.

Comment 1.2:
Figure 2 is readable, but the quality is relatively low. Nevertheless, I do not think this figure is instrumental; it is a simple logical schema of a relational database. Uploading the SQL dump or the SQL schema in an external repository and reference it in the paper would be enough.

Response 1.2:
The image resolution was increased. The detailed referential relationships between the data entities at the attribute level are visualised in the ER diagram. These relationships are important for readers who will go on to use the data set in two ways: first, deeper functionality in the Web app. requires a knowledge of the underlying schema; second, the immediacy of the visualization in the paper will reduce the barrier for users to re-use the raw table dumps for downstream analysis. This intention is now better expressed in the manuscript in the methods section on page 5:
“Its schema, as documented in Figure 2, shows the referential dependencies, foreign keys, and primary key attributes. This is intended to support advanced use of data exploration functionality in the web application. It is also intended to reduce the barrier for users to reuse the raw table dumps for downstream analysis.“

Comment 1.3:
The sentence "we imported an ORACLE SQL data warehouse that employs state-of-the-art database technologies" is not very clear. What do you mean by "imported a data warehouse"? Could you provide more details about the DBMS you used? To my understanding, you designed a relational model. You then implemented it in SQL using an Oracle DBMS (MySQL’ or the native Oracle DBMS?) to store and query the data. Check page 9 description and add some details to avoid confusion. This is not a key passage though, I am sure that you handled the data somehow, and the paper's focus is not on this.

Response 1.3:
Thanks for this useful hint to consider more technical details of our database infrastructure. We revised the mentioned sentence in the manuscript to:

“To support on-line analytical data processing and convenient data access, a data warehouse was built based on the extracted data set. To scale appropriately, an ORACLE Enterprise RDBMS was applied. It

enables analytical real-time SQL queries over millions of data points by means of in-memory materialized views, vectorization and columnar storage. This in turn was the basis to guarantee an appropriate user experience for the subsequently presented web application, which provides interactive, on-line calculation of metrics from NSD citations to various filters and data groupings, to drill-down chart and link data to the original records in ENA and ePMC."

Comment 1.4:
"reference integrity between the tables was checked" -> This is a "weird" statement. Reference integrity is a constraint to guarantee the consistency of data. You "check the integrity" when you store the data in the DB, and if it is not validated, the data cannot be stored in the DB. So, I do not understand this sentence that is not explained anymore. Indeed, the paragraph continues by talking about the SQL queries to count the paper identifiers (this is not directly linked to reference integrity, or at least you should explain what you mean).

Recent analysis about issues related to ORCID ids and duplication of ids can be found here: http://ceur-ws.org/Vol-2816/paper10.pdf

Response 1.4:
We agree with the reviewer that reference integrity is a constraint to guarantee the consistency of data. In the manuscript we were unclear about the level of its use. In contrast to an OLTP centered database implementation of consistency checks by check constraints, such as foreign keys, we applied the reference integrity check in the ETL process to uncover potential reference integrity issues in the data set that results from the data extraction process. Indeed, we used a preliminary data warehouse instance without any formal SQL-DDL foreign key constraints, but SQL-based plausibility checks. E.g. by counting unique paper identifiers, ENA accessions, and country tags and checked number of joinable ENA and ePMC records. This lead to improvements of country references by the add country names synonyms to the country table or the use of a logical OR operator to join the ENA primary publication to ePMC by PMID, DOI or PMCID. A further example was the revision of the first approach to extract ENA accession number as reference to ENA from ePMC records. Because of obvious mismatch of expected counts to actual extracted ones for some exemplar papers, like genome assemblies of plants, we concluded that project accession numbers should also be considered as ENA citation too and revised and expanded the data mining task respectively.

In this context we thank the reviewer for this comment and improved the mentioned paragraph "Retrieval of referenced IDs":

"Next, reference consistency among the extracted ENA and ePMC records were checked by ETL test runs. This resulted in a preliminary data warehouse instance, which allowed SQL-based plausibility checks. Such as the count unique paper identifiers, ENA accessions, and country tags or count the number of records in preliminary joining ENA and ePMC records over different combination of PMID, PMCID and DOI. We specifically checked whether ENA records refer to a valid country. For 217.40 ENA records out of 18.034.192 was it not the case, e.g. country tag "Western Sahara" (ENA accession HM034625) or empty country tag (ENA accession KM654101). Those could in some cases resolved by manual addition of synonyms to the country table that reflects the current valid UN agreed assignments. In case of empty country tags, we found some were annotated with geographical location. However, we left them empty to not intransparently change primary data.
Further we checked, if primary papers referenced in an ENA record exist in the ePMC databases. Here, a total of 6,753,891 ENA records refer either by DOI, PMID, or PMCID to 351,119 ePMC records, i.e. some use DOI only, some DOI and PMID etc."

In respect to the comment "Recent analysis about issues related to ORCID ids and duplication of ids can be found here: http://ceur-ws.org/Vol-2816/paper10.pdf" we added this reference to the manuscript as new citation [24]:

"Baglioni, M., Mannocci, A., Manghi, P., Atzori, C., Bardi, A., and Bruzzo, S. L. Reflections on the misuses of ORCID ids. In CEUR Workshop Proceedings of the 17th Italian Research Conference on Digital Libraries, http://CEUR-WS.org. pp. 117–125, 2021."
Comment 1.5:
Table 1 is not that useful; it can be described in the text that you did the experiment and verified the discrepancies between open access publications and paywalled papers. It is a well-known problem, and it is not analyzed in-depth here. I think you can get rid of it without affecting the quality of the paper.

Response 1.5:
We agree that Table 1 reflects the issues formulated already in the text. A further intention was to show particular cases, where the suggested improvements were beneficial. Following the intention of the GigaScience Data Note policy, the intention here is to give additional interested parties, like software developers, publishers, or data curators some examples for further investigation. Thus, we would like to keep Table 1.

Comment 1.6:
Figure 4, like all the other images, is not readable. I directly accessed the Webapp, which works fine.

Response 1.6:
The quality was improved by increasing the resolution to 1200 dpi. We are sure that the rendering process at the editorial office may benefit and the resulting PDF will have a better readability.

Comment 1.7:
The paper is well-written, and the data collection is fine. Nevertheless, the article is a bit anti-climatic because there, not many provided insights. You discuss what we can do with the data, but little analysis of the data themselves. We could use some more in-depth analysis and a few insights about the achievable outcomes we can get using the collected data. Also, more about the best practices that should be defined in the field would be a nice addition.

Response 1.7:
This observation is particularly true and result of the authors' decision to separate the findings of the underlying study into a technical dataset paper and a policy-focused paper about the analysis, interpretation and conclusions. This paper was announced to the editorial board within the submission process and was submitted to GigaScience (submission ID GIGA-D-21-00228). In order to keep a reasonable timeline for publication, we would like to follow the editor's suggestion and publish a pre-print of a policy analysis paper (DOI: 10.1101/2021.08.02.454535). Furthermore, we will give more background in the WebApp in the details panel below every chart. This will provide users of the data more insight as well.
In respect to the suggested addition to best practice we are note sure about the reviewers intension. Regarding a technical aspect concerning the citation practice of NSD in scientific literature, conclusions can be drawn from the methods section that authors should reference the used sequences as explicitly as possible via a list of individual accession numbers or project accessions according to the citation policy of the respective journals. This aspect was indicated in the section "Data validation and quality control".
Another aspect would be the consistent labeling of the origin of biological material. As noted in section Methods, 15% of the ENA sequences are country tagged. This implies that appropriate regulation on the part of the data repositories could be debated. Although the discussion of this aspect is not in the scope of this data note, the publication of this manuscript could certainly support a discourse.
A best practice in terms of implications for the use and provision of DSI in the context of the CBD negotiations is a comprehensive discourse to which this data note contributes a factual basis. The resulting possible interpretations and implications will be explained in a companion paper as mentioned before.

Answers to comment of reviewer #2
The idea of curating this dataset is both important, and can contribute to the scientific community. Additionally, in most parts, the paper is well written.
However, the manuscript has some major issue that needs to solve before it would be ready for publication.

The Good:
- The dataset presented in the paper can be very useful to the scientific community
- The authors invested many efforts in making the paper reproducible. Both the project's code and dataset are open
- The project has a friendly and helpful web interface.

Things that need to improve:

Comment 2.1:
Although this paper is not a standard research paper, the article is missing more context to other works.

I believe the context of the manuscript will be more explicit by adding a Related Work section that provides an overview of other papers that generated similar datasets.

Response 2.1:
Please see our responses to similar comments by reviewer 1 in response 1.7.

Comment 2.2:
-Most of the analysis is based on the PubMed datasets, which is a relatively small dataset.
There are other open datasets that I think it is important to use to get a fuller picture, such as Microsoft Academic, AMiner, Semantic Scholar, bioXiv, and arXiv.
I understand that performing a full-text search on these datasets can be challenging.
However, the paper's results need to be validated by using some of these datasets.

Response 2.2:
The goal of the study, in the first place, was not to extract all links, however. Rather, the data integration pipeline presented should achieve a good balance between recall and precision with respect to the data integration scenario at hand. The partner in the underlying research project is the EBI. The EBI databases were used with the goal of evaluating possible approaches to quantify DSI usage. In addition, the use case for the analysis was to estimate DSI use in science for policy makers. We also recognize a focus on open access and the value that these open datasets provide. ePMC-API was therefore accepted as a reasonable trade-off between the computational burden of screening more than 18 million ENA accessions and a potential loss of sensitivity.
However, we agree with this comment and will continue to develop the identifier text extraction pipeline in a follow-up project that has already been approved. Some possible improvements have been highlighted in the Data Validation and Quality Control section of the Access Constraints section.
This rationale is now reflected by a text improvement in the "Access Restrictions" section on page 11.

“..., scanning PDF encoded manuscripts, the use of sophisticated text mining methods and integration of commercial text mining software could improve the recall and precision of NDS citation in texts as well. However, as the spirit of the project in which this analysis took place, with a heavy emphasis on open access and ENA API …”

Comment 2.3:
The manuscript's quality needs to be improved (text, figures' resolutions, etc.).

Response 2.3:
As reported in response 1.1 to a similar feedback of reviewer 1, the figures’ resolution was increased to support a suitable PDF rendering in the GigaScience manuscript processing pipeline.

Comment 2.4:
In my opinion, the overall structure of the paper can be improved.

Response 2.4:
Thanks for this comment. The structure of the paper followed the GigaScience authors instruction for data notes. In order to meet this hint, we included before the first subsection “Context” at page 3 an overview of the paper content:
“With this in the background, this Data Note subsequently presents the context of the dataset for quantified NSD use. As such, the method for extracting NSD citations from the scientific literature is described, as well as the technical details of constructing the data warehouse. The Data Validation and Quality Control section discusses the refinement process of the data extraction pipeline and potential shortcomings arising from the available data quality, the provided APIs and the suggested potential improvements. Finally, the potential for reuse of the dataset through the WiLDSI web app is presented, as well as further potential for tracking genetic resource use in the scientific literature and aspects of quantifying DSI use in the context of benefit-sharing discussions under the CBD.”

Comment 2.5:
There is no need to explain the FAIR data principle

Response 2.5:
In the context of the CBD negotiations, the role of NSD/DSI garnered immense interest and raised concern across the international scientific community. This was the background for this data note. The

intention of the mentioned sentence with reference to FAIR was meant to be a transition to the global benefit of free and open use of DSI as demonstrated particularly in science. We refrained from introducing the FAIR criteria. Rather we intended to illustrate the emerging tension field of open science and FAIR as synonym and benefit sharing discussion.

Comment 2.6:
Using Microsoft Academic dataset can assist in mapping between author to a unique id

Response 2.6:
Thanks for the important suggestion. The ambiguous identification of authors and their affiliation is of importance. Thus, we included this aspect in the quality discussion of the manuscript. As follow-up to response 2.2 and extension to response 1.1 for reviewer 1, we agree that Microsoft Academic is a powerful service, that could assist in mapping between author to a unique ID, like ORCID. Nevertheless, we would have needed to purchase a commercial license to use the service in a programmatic way in the data extraction pipeline and to publish the results. As noted above, the aim of the manuscript was to apply the open public tools available from ePMC and ENA to ensure a broad reproducibility of the presented DSI quantification pipeline in context of the policy discussions.

Comment 2.7:
Mapping between an institute or location to a country can be more accurately done by utilizing geolocation code packages, such as geopy

Response 2.7:
Thanks to refeer by this comment to the importance of having consistently geolocated authors and NSD material orign. In fact 15% of all ENA sequences comprise a country tag as provenance in terms of international law and in context of the data notes objective. In respect to the potential envoronmental context of the "pick-up" location of bio material, 23% of them annotated by specific geo-coordinates, which could be used to validate the material provenance of each ENA deposited sequence data. But in respect to the background of this paper it seamed to be sufficient to considered the country tag only.
In case of scientific literature, the use of geolocation to validate or curate authors affiliation, if unique institutes identifiers, for example by ROR identifier, would be broadly available. An indirect geo location of authors affiliation by ROR use in authors ORCID record would be a further source for the near future. But currently, a minor fraction of all authors/paper pairs are annotated by an ORCID. Furthermore, how much of these ORCIDs are maintained consistently with authors affiliation history is unknown.

Answers to comment of reviewer #3
I have reviewed this manuscript with integrity, but I'm a little confused about it because I usually use NCBI PubMed/GenBank data. If my points are off the mark, please point them out.

Comment 3.1:
In NCBI PubMed, the nucleotide sequence entries referenced in the article are listed in PubMed data as external DB links (although not perfect), and by extracting these, the relationship between the PubMed and Nucleotide entries can be extracted. The NCBI website also provides these links from Nucleotide in the Related information section (e.g. https://pubmed.ncbi.nlm.nih.gov/19193256/). I found that the ePMC website also has a link in the Data section for nucleotide sequence entries referenced in the paper (e.g., https://europepmc.org/article/MED/19193256). Do you use any of these external links in ePMC data in this work? I think it is very difficult to extract nucleotide IDs by text mining, especially since Nucleotide sequence IDs are not in a fixed format. I think these links will be a great help in doing text mining.

Response 3.1:
Thanks for the comment. Indeed, we also use the one extended version of the pipeline used in ePMC to identify ENA accessions, rather than the pipeline from NCBI Genbank. However, the goal of the study was not so primary as to extract all links. Rather, the data integration pipeline presented was intended to achieve a good balance between recall and precision with respect to the data integration scenario at hand. This is because the underlying literature as well as the sequence data are subject to dynamics and the citation quality of the NSD are very heterogeneous, as discussed in the "Data Validation" section.
Therefore, the initial focus for this dataset was on a quantitative assessment of DSI use under manual quality review.
This resulted in this first integrated quality-checked dataset, which is intended to be published in conjunction with a web application for its exploratory evaluation.

However, we agree with the reviewer, have the potential for further work already in the manuscript, and will take up these points in a follow-up project that has already been approved.

Comment 3.2:
2. In NCBI PubMed, MeSH keywords are assigned to each article for indexing the literature. MeSH keywords also include country keywords (e.g. https://pubmed.ncbi.nlm.nih.gov/19193256/). In ePMC Is it possible to use keywords like MeSH in ePMC? Do you have any opinions about using such country keywords?

Response 3.2:
Thanks for pointing this out. Indeed, some primary publications on NSD annotate the material or origin as a keyword in the corresponding publication in addition to the Country tag in ENA for the deposited sequences. In this particular case, Kenya is also consistently annotated to the 92 ENA sequences linked to this primary publication. However, a consistency analysis between keywords in primary publications, and the country tag in the original ENA was not the objective of this work.

Comment 3.3:
I found some great statistics and visualizations of this data on the site the authors provide about it. I would be happy to show these in this manuscript as a result of this work, but please follow the journal's policies and precedents.

Response 3.3:
Thanks for this. Nevertheless, we announced to the editorial board a companion paper that will more deeply elaborate and interpret the charts. This will be linked to this data note. Furthermore, the charts in the Web application have been updated and include more detail to the visualised quantification in respect to the DSI discussions under the Convention on Biological Diversity. In concrete the charts were consolidated and a scatter plot in panel 6 - "Network Graph" was added that visualise details of the global relationship between providing and using of DSI. The charts in panel 2 - "country use of DSI" and panel 3 - "World Maps" are updated and count all publications instead of only secondary publications. We updated the charts in both panels because it's the same data but visualised in different types of graphs. Moreover, a new graph was added in panel 5 - "coalition collaboration" which displays the DSI use in all publications from the different economic groups. This is now the first graph on this page. We also moved chart 5.6 - "BRICS-G77-OECD consumption/contribution" to the fourth position. In panel 4 - "hemisphere collaboration" graph 4.4 - "Distribution of publications using DSI with authors of n-different countries" was deleted.

Comment 3.4:
Do the authors think that users should reuse the created data for this product? Or is it recommended that users create their own data using the creation program? If the former, what is your plan for the frequency of updating the data?

Response 3.4:
We hope that readers will do both: some will explore the data using the web app and others will re-run the analysis on future or otherwise expanded datasets. Regarding data updates, please refer to response 3.1. The aforementioned follow-up project will work on a data update pipeline, with the goal of updating the data every six months.

Comment 3.5:
In Figure 1, I felt that it would be easier for the reader to understand if I emphasized (by changing the line or fill of the box) whether the data in each step is Nucleotide data, literature data, or ID pairs extracted from those data.

Response 3.5:
Indeed, the layout of Figure 1 aimed to illustrate the complex data extraction and integration workflow in a sketchy manner. The current highlighting and sectioning into a, b and c intended to reflect and logically group the processing steps. The suggestion to emphasize the data domain would surely improve illustrative quality. Thanks for this hint, which we followed and reshaped the Figure 1 towards a more visually clear tagging of nucleotide and literature data.

Close
