# Supplementary material for: Quantitative monitoring of nucleotide sequence data from genetic resources in context of their citation in the scientific literature
Source: Gigascience. 2021 Dec 29;10(12):giab084. doi: 10.1093/gigascience/giab084 (PMC8716361; doi:10.1093/gigascience/giab084)
Supplement: giab084_Response_to_Reviewer_Comments_Revision_1 [file giab084_response_to_reviewer_comments_revision_1.pdf]

Author's Response To Reviewer Comments

Close

Dear editors, dear reviewers,

We are very appreciative of the review and further comments on our article. In the following letter, we answer all questions and provide comments on the intended revisions. All changes in the manuscript for this second revision are highlighted in green. The changes of first revision are highlighted still in yellow. Please note that the co-author "Florian Zunder" provided his ORCID (0000-0001-6495-8611).
The point-by-point response to the reviewers' comments can be found hereafter.

Answers to comment of reviewer #1

Comment 1.1:
The authors comprehensively answered to this reviewer comments. The quality of the paper is improved and the modifications are in line with what was expected.
I have no further observations.

Response 1.1:
Thank you very much for the review.

Answers to comment of reviewer #2

Comment 2.1:
After reading the submitted "policy paper," the goal and contributions of this study and dataset became clear.
I believe this dataset and data visualization interface can be beneficial for the academic community.

Response 2.1:
Thanks for the reviewer's support and comments to improve the manuscript.

Comment 2.2:
It is very challenging to understand Figure 1. I recommend adding additional figures that better explains how each part of the system work with more details.

Response 2.2:

Thanks for the hint to improve the Figure 1. Indeed, a visualization of a complex data flow process is challenging task. The image objective is to give reasonable abstraction to the core components of the data flow and the statistics of processed data. To visualize the data sets, data domains and processing step, we sub-divided logical connected sub-processes as boxes, color-coded the two data domains, numbered each ELT data processing step accordingly and described the step in the figure caption. Furthermore, we improved the layout of the data flow visualization.
To direct address the reviewer hint, we add an ELT process flow diagram as sub-figure that shows the sequence and dependencies within the ELT process.

Comment 2.3:
Even though the quality of the figures was improved, they are still of low quality, and it is hard to read the figures, especially Figure 4.

Response 2.3:
The resolution of the Figures is 1200 dpi. We will crosscheck the rendering quality of the proofread within the journal production team carefully.

Comment 2.3:
The paper needs to be carefully proofread for punctuation mistakes.

Response 2.3:
Thanks for the hint. The manuscript undergoes an additional proof read to correct the punctuation and further grammatical and spelling mistakes. The changes where highlighted accordingly.

Answers to comment of reviewer #3

Comment 3.1:
I was convinced by the author's response to my previous comment.

Response 3.1:

Thank you very much for the review.

Close
