# Supplementary material for: Quantitative monitoring of nucleotide sequence data from genetic resources in context of their citation in the scientific literature
Source: Gigascience. 2021 Dec 29;10(12):giab084. doi: 10.1093/gigascience/giab084 (PMC8716361; doi:10.1093/gigascience/giab084)
Supplement: giab084_Reviewer_1_Report_Original_Submission — Gianmaria Silvello, Ph.D. -- 5/10/2021 Reviewed [file giab084_reviewer_1_report_original_submission.pdf]

Reviewer Report

Title: Quantitative monitoring of nucleotide sequence data from genetic resources in context of their citation in the scientific literature

Version: Original Submission **Date:** 5/10/2021

Reviewer name: Gianmaria Silvello, Ph.D.

Reviewer Comments to Author:

Figure 1 is not readable. The sampling process lowered the quality of the image and made the text not readable. Please, use vectorial images (e.g., PDF or EPS). Anyhow, I could understand the process from the descriptive text.
Figure 2 is readable, but the quality is relatively low. Nevertheless, I do not think this figure is instrumental; it is a simple logical schema of a relational database. Uploading the SQL dump or the SQL schema in an external repository and reference it in the paper would be enough.
The sentence "we imported an ORACLE SQL data warehouse that employs state-of-the-art database technologies" is not very clear. What do you mean by "imported a data warehouse"? Could you provide more details about the DBMS you used? To my understanding, you designed a relational model. You then implemented it in SQL using an Oracle DBMS (MySQL? or the native Oracle DBMS?) to store and query the data. Check page 9 description and add some details to avoid confusion. This is not a key passage though, I am sure that you handled the data somehow, and the paper's focus is not on this.
"reference integrity between the tables was checked" -> This is a "weird" statement. Reference integrity is a constraint to guarantee the consistency of data. You "check the integrity" when you store the data in the DB, and if it is not validated, the data cannot be stored in the DB. So, I do not understand this sentence that is not explained anymore. Indeed, the paragraph continues by talking about the SQL queries to count the paper identifiers (this is not directly linked to reference integrity, or at least you should explain what you mean).
Recent analysis about issues related to ORCID ids and duplication of ids can be found here: http://ceur-ws.org/Vol-2816/paper10.pdf
Table 1 is not that useful; it can be described in the text that you did the experiment and verified the discrepancies between open access publications and paywalled papers. It is a well-known problem, and it is not analyzed in-depth here. I think you can get rid of it without affecting the quality of the paper.
Figure 4, like all the other images, is not readable. I directly accessed the Webapp, which works fine.
The paper is well-written, and the data collection is fine. Nevertheless, the article is a bit anti-climatic because there, not many provided insights. You discuss what we can do with the data, but little analysis of the data themselves. We could use some more in-depth analysis and a few insights about the achievable outcomes we can get using the collected data. Also, more about the best practices that should be defined in the field would be a nice addition.

Level of Interest

Please indicate how interesting you found the manuscript: Choose an item.

Quality of Written English

Please indicate the quality of language in the manuscript: Choose an item.

Declaration of Competing Interests

Please complete a declaration of competing interests, considering the following questions:

- Have you in the past five years received reimbursements, fees, funding, or salary from an organisation that may in any way gain or lose financially from the publication of this manuscript, either now or in the future?
- Do you hold any stocks or shares in an organisation that may in any way gain or lose financially from the publication of this manuscript, either now or in the future?
- Do you hold or are you currently applying for any patents relating to the content of the manuscript?
- Have you received reimbursements, fees, funding, or salary from an organization that holds or has applied for patents relating to the content of the manuscript?
- Do you have any other financial competing interests?
- Do you have any non-financial competing interests in relation to this paper?

If you can answer no to all of the above, write 'I declare that I have no competing interests' below. If your reply is yes to any, please give details below.

I have no competing interests.

I agree to the open peer review policy of the journal. I understand that my name will be included on my report to the authors and, if the manuscript is accepted for publication, my named report including any attachments I upload will be posted on the website along with the authors' responses. I agree for my report to be made available under an Open Access Creative Commons CC-BY license (http://creativecommons.org/licenses/by/4.0/). I understand that any comments which I do not wish to be included in my named report can be included as confidential comments to the editors, which will not be published.

Choose an item.

To further support our reviewers, we have joined with Publons, where you can gain additional credit to further highlight your hard work (see: https://publons.com/journal/530/gigascience). On publication of this paper, your review will be automatically added to Publons, you can then choose whether or not to claim your Publons credit. I understand this statement.

Yes Choose an item.
