# Supplementary material for: Quantitative monitoring of nucleotide sequence data from genetic resources in context of their citation in the scientific literature
Source: Gigascience. 2021 Dec 29;10(12):giab084. doi: 10.1093/gigascience/giab084 (PMC8716361; doi:10.1093/gigascience/giab084)
Supplement: giab084_Reviewer_2_Report_Original_Submission — Michael Fire, Ph.D -- 5/25/2021 Reviewed [file giab084_reviewer_2_report_original_submission.pdf]

Reviewer Report

Title: Quantitative monitoring of nucleotide sequence data from genetic resources in context of their citation in the scientific literature

Version: Original Submission **Date:** 5/25/2021

Reviewer name: Michael Fire, Ph.D

Reviewer Comments to Author:

The idea of curating this dataset is both important, and can contribute to the scientific community. Additionally, in most parts, the paper is well written.
However, the manuscript has some major issue that needs to solve before it would be ready for publication.
The Good:
- The dataset presented in the paper can be very useful to the scientific community
- The authors invested many efforts in making the paper reproducible. Both the project's code and dataset are open
- The project has a friendly and helpful web interface.
Things that need to improve:
Major Issues:
- Although this paper is not a standard research paper, the article is missing more context to other works.
I believe the context of the manuscript will be more explicit by adding a Related Work section that provides an overview of other papers that generated similar datasets.
- Most of the analysis is based on the PubMed datasets, which is a relatively small dataset.
There are other open datasets that I think it is important to use to get a fuller picture, such as Microsoft Academic, AMiner, Semantic Scholar, bioXiv, and arXiv.
I understand that performing a full-text search on these datasets can be challenging.
However, the paper's results need to be validated by using some of these datasets.
- The manuscript's quality needs to be improved (text, figures' resolutions, etc.).
Minor Issues:
- In my opinion, the overall structure of the paper can be improved.
- There is no need to explain the FAIR data principle
- Using Microsoft Academic dataset can assist in mapping between author to a unique id
- Mapping between an institute or location to a country can be more accurately done by utilizing geolocation code packages, such as geopy

Level of Interest

Please indicate how interesting you found the manuscript: Choose an item.

Quality of Written English

Please indicate the quality of language in the manuscript: Choose an item.

Declaration of Competing Interests

Please complete a declaration of competing interests, considering the following questions:

- Have you in the past five years received reimbursements, fees, funding, or salary from an organisation that may in any way gain or lose financially from the publication of this manuscript, either now or in the future?
- Do you hold any stocks or shares in an organisation that may in any way gain or lose financially from the publication of this manuscript, either now or in the future?
- Do you hold or are you currently applying for any patents relating to the content of the manuscript?
- Have you received reimbursements, fees, funding, or salary from an organization that holds or has applied for patents relating to the content of the manuscript?
- Do you have any other financial competing interests?
- Do you have any non-financial competing interests in relation to this paper?

If you can answer no to all of the above, write 'I declare that I have no competing interests' below. If your reply is yes to any, please give details below.

I declare that I have no competing interests

I agree to the open peer review policy of the journal. I understand that my name will be included on my report to the authors and, if the manuscript is accepted for publication, my named report including any attachments I upload will be posted on the website along with the authors' responses. I agree for my report to be made available under an Open Access Creative Commons CC-BY license (http://creativecommons.org/licenses/by/4.0/). I understand that any comments which I do not wish to be included in my named report can be included as confidential comments to the editors, which will not be published.

Choose an item.

To further support our reviewers, we have joined with Publons, where you can gain additional credit to further highlight your hard work (see: https://publons.com/journal/530/gigascience). On publication of this paper, your review will be automatically added to Publons, you can then choose whether or not to claim your Publons credit. I understand this statement.

Yes Choose an item.
