# Supplementary material for: Quantitative monitoring of nucleotide sequence data from genetic resources in context of their citation in the scientific literature
Source: Gigascience. 2021 Dec 29;10(12):giab084. doi: 10.1093/gigascience/giab084 (PMC8716361; doi:10.1093/gigascience/giab084)
Supplement: giab084_Reviewer_3_Report_Original_Submission — Takeru Nakazato, Ph.D. -- 5/31/2021 Reviewed [file giab084_reviewer_3_report_original_submission.pdf]

Reviewer Report

Title: Quantitative monitoring of nucleotide sequence data from genetic resources in context of their citation in the scientific literature

Version: Original Submission **Date:** 5/31/2021

Reviewer name: Takeru Nakazato, Ph.D.

Reviewer Comments to Author:

I have reviewed this manuscript with integrity, but I'm a little confused about it because I usually use NCBI PubMed/GenBank data. If my points are off the mark, please point them out.
1. In NCBI PubMed, the nucleotide sequence entries referenced in the article are listed in PubMed data as external DB links (although not perfect), and by extracting these, the relationship between the PubMed and Nucleotide entries can be extracted. The NCBI website also provides these links from Nucleotide in the Related information section (e.g. https://pubmed.ncbi.nlm.nih.gov/19193256/). I found that the ePMC website also has a link in the Data section for nucleotide sequence entries referenced in the paper (e.g., https://europepmc.org/article/MED/19193256). Do you use any of these external links in ePMC data in this work? I think it is very difficult to extract nucleotide IDs by text mining, especially since Nucleotide sequence IDs are not in a fixed format. I think these links will be a great help in doing text mining.
2. In NCBI PubMed, MeSH keywords are assigned to each article for indexing the literature. MeSH keywords also include country keywords (e.g. https://pubmed.ncbi.nlm.nih.gov/19193256/). In ePMC Is it possible to use keywords like MeSH in ePMC? Do you have any opinions about using such country keywords?
3. I found some great statistics and visualizations of this data on the site the authors provide about it. I would be happy to show these in this manuscript as a result of this work, but please follow the journal's policies and precedents.
4. Do the authors think that users should reuse the created data for this product? Or is it recommended that users create their own data using the creation program? If the former, what is your plan for the frequency of updating the data?
5. In Figure 1, I felt that it would be easier for the reader to understand if I emphasized (by changing the line or fill of the box) whether the data in each step is Nucleotide data, literature data, or ID pairs extracted from those data.

Level of Interest

Please indicate how interesting you found the manuscript: Choose an item.

Quality of Written English

Please indicate the quality of language in the manuscript: Choose an item.

Declaration of Competing Interests

Please complete a declaration of competing interests, considering the following questions:

- Have you in the past five years received reimbursements, fees, funding, or salary from an organisation that may in any way gain or lose financially from the publication of this manuscript, either now or in the future?
- Do you hold any stocks or shares in an organisation that may in any way gain or lose financially from the publication of this manuscript, either now or in the future?
- Do you hold or are you currently applying for any patents relating to the content of the manuscript?
- Have you received reimbursements, fees, funding, or salary from an organization that holds or has applied for patents relating to the content of the manuscript?
- Do you have any other financial competing interests?
- Do you have any non-financial competing interests in relation to this paper?

If you can answer no to all of the above, write 'I declare that I have no competing interests' below. If your reply is yes to any, please give details below.

I declare that I have no competing interests

I agree to the open peer review policy of the journal. I understand that my name will be included on my report to the authors and, if the manuscript is accepted for publication, my named report including any attachments I upload will be posted on the website along with the authors' responses. I agree for my report to be made available under an Open Access Creative Commons CC-BY license (http://creativecommons.org/licenses/by/4.0/). I understand that any comments which I do not wish to be included in my named report can be included as confidential comments to the editors, which will not be published.

Choose an item.

To further support our reviewers, we have joined with Publons, where you can gain additional credit to further highlight your hard work (see: https://publons.com/journal/530/gigascience). On publication of this paper, your review will be automatically added to Publons, you can then choose whether or not to claim your Publons credit. I understand this statement.

Yes Choose an item.
